# Global, regional, and country seroprevalence of *Toxoplasma gondii* in pregnant women: a systematic review, modelling and meta-analysis

**DOI:** 10.1038/s41598-020-69078-9

**Published:** 2020-07-21

**Authors:** Jean Joel Bigna, Joel Noutakdie Tochie, Dahlia Noelle Tounouga, Anne Olive Bekolo, Nadia S. Ymele, Emilie Lettitia Youda, Paule Sandra Sime, Jobert Richie Nansseu

**Affiliations:** 1 Department of Epidemiology and Public Health, Centre Pasteur of Cameroon, PO Box 1274, Yaoundé, Cameroon; 2 Faculty of Medicine and Biomedical Sciences, University of Yaoundé I, Yaoundé, Cameroon; 3 Higher Institute of Health Sciences, University of Mountains, Bangangté, Cameroon; 4 EHESP French School of Public Health, University of Sorbonne Paris Cité, Paris, France; 5 Department of Disease, Epidemics and Pandemics Control, Ministry of Public Health, Yaoundé, Cameroon

**Keywords:** Infectious diseases, Epidemiology

## Abstract

Efficient health-care for pregnant women require accurate data on the prevalence of toxoplasmosis in pregnancy at global, regional, and country levels. In this systematic review with meta- and modelling-analysis, we searched PubMed, EMBASE, Web of Knowledge, Global Index Medicus, and Africa Journal Online to identify studies that reported enough data to compute the immunoglobulins (Ig) M or G seroprevalence estimates of *Toxoplasma gondii* in pregnant women up to December 31st, 2018, without any language restriction. The global and regional estimates were done using a random-effects meta-analysis. We included 250 studies with 723,655 pregnant women. The global IgM seroprevalence was 1.9% (95%CI: 1.7–2.3). At the regional level, Eastern Mediterranean had the highest IgM seroprevalence (4.1%, 95%CI: 2.8–5.5) and The Americas, the lowest (1.1%, 0.8–1.4), with a statistically significant difference between WHO regions (*p* < 0.0001). The global IgG seroprevalence was 32.9% (95%CI: 29.4–36.4). Among WHO regions, The Americas had the highest prevalence (45.2%, 95%CI: 33.4–53.4) and Western Pacific the lowest (11.2%, 7.8–15.1), with a statistically significant difference between regions (*p* < 0.0001). This study presents a high toxoplasma seropositivity in pregnant women at global, regional and country levels, with a consequential high risk of maternal and congenital toxoplasmosis.

## Introduction

Globally, more than 211 million pregnancies occur each year. Without adequate follow-up and appropriate preventive care, each of these pregnancies is prone to complications including miscarriage, stillbirth, foetal death, neurologic and neurocognitive deficits, chorio-retinitis, and child disability^1,2^. These poor outcomes can be due to some infections during pregnancy including *Toxoplasma gondii* infection^2^. This infection among pregnant women requires early diagnosis and treatment to improve mother and child health^3^. Pregnant women can be infected through zoonotic transmission or foodborne transmission^4^. In humans, infection is usually acquired by consumption and manipulation of raw or undercooked meat. Infection can also be acquired through eating unwashed vegetables and fruits, drinking water containing oocytes excreted in the faeces of infected cats, or contact with cat litter or soil. When this parasite is acquired during pregnancy, the parasite can be transmitted across the placenta to the foetus, resulting in congenital toxoplasmosis^2,5^. In the context of vertical transmission from mother to child during pregnancy, it is estimated an average of 190,100 incident cases of congenital toxoplasmosis yearly, with 1.5 neonatal cases occurring per 1,000 live births globally^6^.

One third of the general population is infected with toxoplasma with high heterogeneity between countries and regions^4^. This heterogeneity can also be found in the population of pregnant women, however, to the best of our knowledge, there is no study exploring the global, regional, and country distribution of *Toxoplasma gondii* infection among pregnant women. To set priorities for public health policy, funding for public health interventions, and health-care planning for curbing the burden of toxoplasmosis on pregnancy outcomes and neonatal health, it is necessary to have accurate data on the prevalence of toxoplasmosis in pregnancy in all countries and territories. To date, Rostami and colleagues provided evidence on acute and latent toxoplasmosis in pregnancy at global, regional, and national levels where data are available^7,8^. We herein present the first systematic review with meta-analysis to provide prevalence of toxoplasmosis in pregnancy in all countries and territories where data are available; and using Bayesian analysis, we estimated the prevalence where data are not yet available. This study provides an accurate understanding of the scope of this public health concern and aims to inform and draw the attention of researchers, health-care practitioners, public health authorities, policymakers, and governments towards the pending burden of toxoplasmosis in pregnancy.

## Methods

### Search strategy and selection criteria

The protocol of this study was registered with PROSPERO, CRD42019125572 and published in a peer-review journal^9^. In brief, we considered cross-sectional, case–control, and baseline data of cohort studies reporting the prevalence (or enough data to compute this estimate i.e. number of cases and sample size) of *Toxoplasma gondii* infection measured by the presence of immunoglobulins (Ig) G or M in the sera of pregnant women regardless of their geographical location.

We searched Medline through PubMed, Excerpta Medica Database (EMBASE), Web of Knowledge (including Web of Science Core collection, Current Contents Connect, KCI-Korean Journal Database, Russian Science Citation Index, Scientific Electronic Library Online (SciELO) Citation index), Africa Journal Online, Global Index Medicus (including Literatura Latino Americana em Ciências da Saúde (LILACS), Index Medicus for South-East Asia Region (IMSEAR), Western Pacific Region Medicus (WPRIM), Index Medicus for the Eastern Mediterranean Region (IMERMR), Africa Index Medicus (AIM)) up to December 31st 2018. We searched records regardless of language of publication and geographic situation. The search strategy in databases is presented in the published protocol^9^. Key search terms included: “pregnant women”, “pregnancy”, “toxoplasmosis”, and “toxoplasma”. We manually searched the reference list of eligible articles and relevant reviews to identify additional studies.

### Data extraction and management

Two investigators (JJB and JRN) independently performed the selection of records based on titles and abstracts; followed by selection based on the full text through the Rayyan application^10^. Disagreements were solved by discussion and consensus. Three pairs of investigators (JNT, DNT, AOB, NSY, PSS, JJB) performed the methodological quality assessment of finally included studies with the Joanna Briggs Institute tool for prevalence studies^11^*.* Disagreements in each pair of investigators were solved by discussion.

Three pairs of investigators (JNT, DNT, AOB, NSY, PSS, JJB) independently extracted data. Disagreements in each pair of investigators were solved by discussion. Using a pretested form, we extracted bibliometric information, country of recruitment, period of participants’ inclusion, site of recruitment (antenatal care unit, delivery unit, hospital-based, and population-based), representativeness of the sample (national, regional/multisite, one site), number of pregnant women tested for *Toxoplasma gondii*, method of sampling (probabilistic sampling versus non probabilistic sampling), number of pregnant women infected with *Toxoplasma gondii*. Countries of recruitment of participants were grouped in regions according to the World Health Organization (WHO) regional classification: Africa, The Americas, South-East Asia, Europe, Eastern Mediterranean, and Western Pacific.

### Data synthesis and analysis

We performed analyses with the statistical software R, version 3.6.1 (R core Team, The R Foundation for statistical computing, Vienna, Austria). For each country, we estimated the prevalence based on empirical studies if there was (1) at least one nationally representative study or at least (2) two non-nationally representative studies. Regional and global estimates were based only on empirical data. Prevalence estimates were reported with both their 95% confidence interval (95%CI) and 95% prediction interval. We conducted a sensitivity analysis including only studies with a low risk of bias to assess the robustness of crude findings. We fitted a three-level meta-analysis with the following hierarchy: each study nested in the country of recruitment, and data from countries nested in WHO regions. Then, we estimated an adjusted global picture considering between- and within-cluster heterogeneity at country and WHO region levels in a hierarchical model. Prevalence pooling was done with the Freeman-Tukey double arcsine transformation using a random-effects meta-analysis model^12^. Funnel plots and the Egger’s test served for detecting presence of publication bias^13^. A *p* value < 0.10 on Egger test was considered indicative of statistically significant publication bias. Heterogeneity was evaluated by the χ^2^ test on Cochrane’s Q statistic^14^, which was quantified by I^2^ values. The I^2^ statistic estimates the percentage of total variation across studies due to true between-study differences rather than chance. In general, I^2^ values greater than 60–70% indicate the presence of substantial heterogeneity^15^. Estimates based on empirical data were done with ‘*meta*’ and ‘*metafor*’ packages. In addition, a meta-regression analysis was performed to identify and quantify sources of heterogeneity. The final multivariable model included variables if *p* < 0.20 in the univariable analysis.

For countries with one or no empirical studies, we predicted the country's prevalence using a Bayesian generalized non-linear multilevel modelling with a binomial family and a logit link. We used the Markov chain Monte Carlo (MCMC) algorithm with objective priors. Convergence was monitored and 5,000 post-burn-in samples were obtained from the posterior distribution of model parameters, which were then used to obtain the posterior distributions of *Toxoplasmosis gondii* seroprevalence. The reported credible intervals (CrI) represent the 2.5–97.5 percentiles of the posterior distributions. The model included WHO region and country-specific gross domestic per capita corresponding to the year in which the study was conducted with a smoothing term^16^. With regards to WHO regions, we split WHO EUR and WHO AMR into high-income and low-income regions (i.e., European Union [EU] member states vs. non-EU-member states, and Canada & USA vs. all remaining countries in the Americas, respectively)^17^. We estimated the pseudo R-squared to have the explained variance in the fitted model for prediction. The Bayesian modelling was done with the ‘*brms*’ package.

## Results

### Study selection and characteristics

We initially identified 4,117 records and finally retained 248 studies (250 prevalence data) (Supplementary Fig. 1). Agreement between investigators on selection based on title and abstract was κ = 0.78 and κ = 0.93 for final inclusion. The reference list of the 250 data points is available in the Appendix.

**Figure 1:**
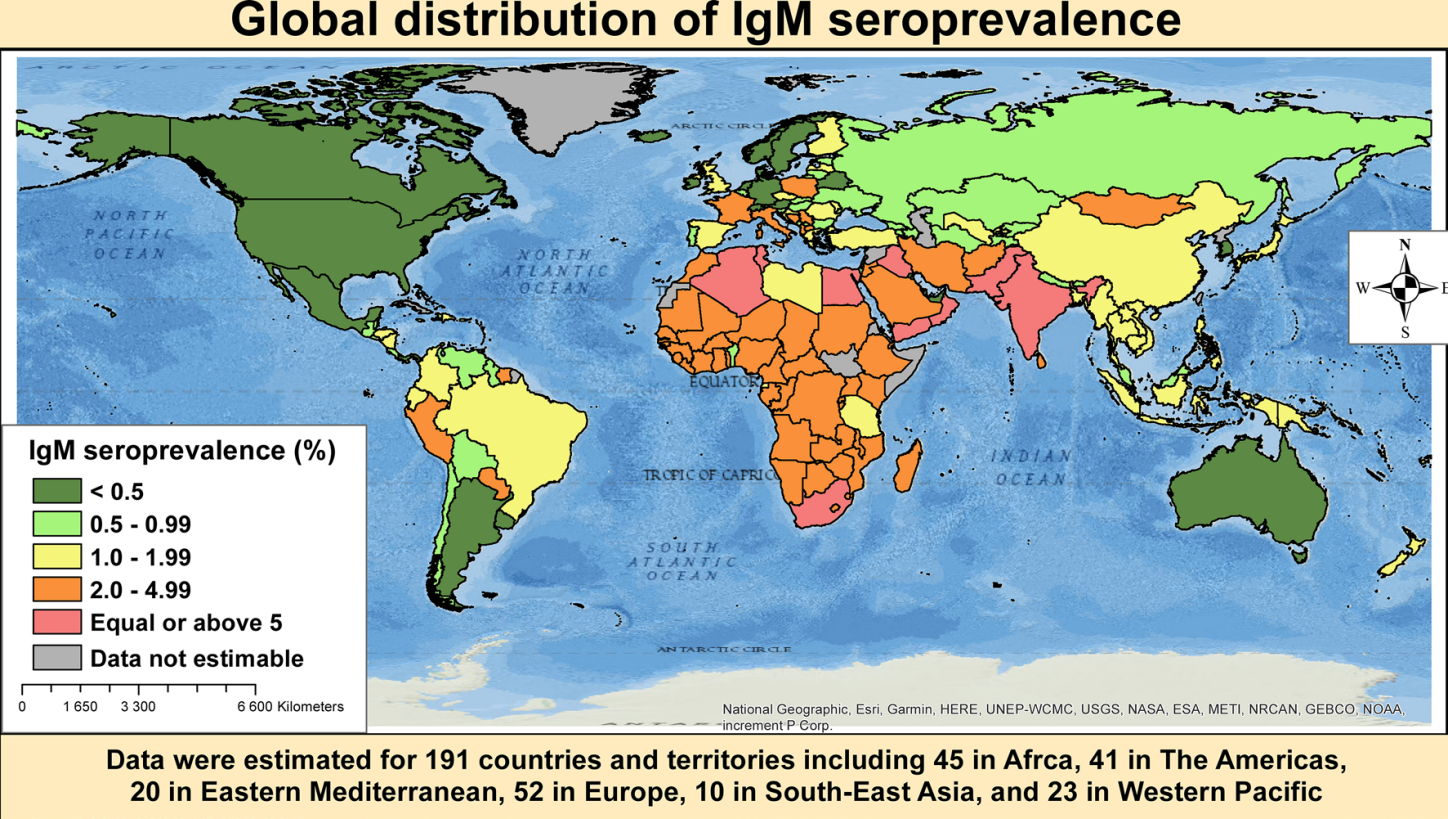
Global distribution of *Toxoplasma gondii* immunoglobulins M seroprevalence in pregnant women.

In total, 723,655 pregnant women were included. Data were collected in pregnant women from 1976 to 2017 and published between 1979 and 2018. Sixty-five studies were from Europe, 51 from The Americas, 47 from Eastern Mediterranean, 39 from Africa, 29 from Western Pacific, and 19 from South-East Asia. Five studies were nationally-representative, 53 were multisite, 180 were one-site, and 12 did not specify the number of sites. One hundred and forty-five studies (58.0%) presented a low risk of bias, 100 (40.0%) a moderate risk of bias, and 5 studies (2.0%) presented a high risk of bias. Characteristics of included studies are depicted in Supplementary Table 1.

**Table 1:** Meta-analysis seroprevalence of *Toxoplasma gondii* in the global and regional population of pregnant women.

	Prevalence	95% confidence interval	95% prediction interval	N studies	Sample	Heterogeneity		Egger test, *p* value	Difference, *p* value
						I^2^	*p* value		
**Immunoglobulins M**									
Global	1.9	1.7–2.3	0.0–7.2	173	464,162	97.7	< 0.0001	< 0.0001	
Low risk of bias	1.8	1.4–2.2	0.0–6.9	95	328,855	98.1	< 0.0001	< 0.0001	
WHO Regions									
Eastern Mediterranean	4.1	2.8–5.5	0.0–15.7	36	14,080	93.6	< 0.0001	0.356	< 0.0001
Africa	2.9	1.7–4.3	0.0–12.0	22	8,128	91.1	< 0.0001	0.547	
South-East Asia	2.1	0.9–3.8	0.0–12.8	17	12,432	95.7	< 0.0001	0.003	
Western Pacific	1.6	1.0–2.3	0.0–6.0	22	32,912	94.6	< 0.0001	0.589	
Europe	1.2	0.8–1.8	0.0–6.5	39	148,050	98.6	< 0.0001	0.306	
Americas	1.1	0.8–1.4	0.0–3.4	37	248,560	96.8	< 0.0001	< 0.0001	
Country level of income									
Low	2.4	1.1–4.3	0.0–13.1	14	4,890	92.3	< 0.0001	0.703	0.004
Lower-middle	4.5	2.6–6.8	0.0–20.3	25	9,430	95.3	< 0.0001	0.154	
Upper-middle	1.7	1.4–2.0	0.0–5.5	96	192,305	95.6	< 0.0001	< 0.0001	
High	1.4	0.9–2.0	0.0–6.2	38	257,537	98.9	< 0.0001	0.012	
Year of study									
≤ 2000	1.1	0.8–1.5	0.0–3.8	32	244,296	97.7	< 0.0001	0.0007	< 0.0001
> 2000	2.2	1.8–2.5	0.0–8.1	141	219,866	96.7	< 0.0001	< 0.0001	
**Immunoglobulins G**									
Global	32.9	29.4–36.4	0.1–86.8	234	520,360	99.9	< 0.0001	< 0.0001	
Low risk of bias	35.8	31.5–40.2	1.2–84.8	133	350,927	99.9	< 0.0001	< 0.0001	
WHO regions									
Americas	45.2	33.4–57.3	0.0–100	47	181,421	100.0	< 0.0001	< 0.0001	< 0.0001
Eastern Mediterranean	39.7	34.5–45.0	9.2–75.6	46	26,494	98.7	< 0.0001	0.033	
Africa	36.5	27.5–46.0	0.1–90.0	35	11,658	99.1	< 0.0001	0.627	
Europe	30.0	26.2–33.9	5.6–63.4	63	248,065	99.8	< 0.0001	0.010	
South-East Asia	24.6	19.4–30.2	5.4–51.7	17	8,751	96.9	< 0.0001	0.551	
Western Pacific	11.2	7.8–15.1	0.0–37.0	26	43,917	99.3	< 0.0001	0.286	
Country level of income									
Low	38.3	26.2–51.1	0.0–93.4	21	7,124	99.2	< 0.0001	0.057	0.017
Lower-middle	30.9	25.4–36.7	4.7–67.1	34	15,896	98.2	< 0.0001	0.080	
Upper-middle	36.6	31.1–42.3	0.0–92.5	120	242,132	99.9	< 0.0001	0.0008	
High	25.0	19.9–30.3	0.2–70.7	59	255,208	99.9	< 0.0001	0.0004	
Year of study									
≤ 2000	30.0	22.4–38.1	0.0–85.9	46	194,874	99.9	< 0.0001	0.0006	0.415
> 2000	33.6	29.9–37.5	0.3–85.1	188	325,486	99.8	< 0.0001	< 0.0001	

### IgM seroprevalence of *Toxoplasma gondii* among pregnant women

We were able to estimate 26 country prevalence rates based on empirical data. Accordingly, the three countries with the highest prevalence of IgM seroprevalence were Yemen (6.0%, 95%CI: 1.6–12.8), Egypt (4.4%, 1.0–10.0), and Saudi Arabia (4.1%, 2.2–6.5). On the contrary, the three countries with the lowest IgM seroprevalence were New Zealand (0.2%, 0.1–0.4), South Korea (0.1%, 0.0–0.4), and USA (0.01%, 0.001–0.02) (Supplementary Table 2). When considering the predicted prevalence, the first 17 countries with the highest prevalence were from WHO Eastern Mediterranean and Africa regions. The five highest predicted prevalence were from Lebanon (10.8%, 95%CrI: 10.5–11.2), Tunisia (6.9%, 6.6–7.2), South Africa (6.2%, 5.8–6.5), Egypt (6.2%, 5.9–6.5), and Algeria (5.7%, 5.3–6.0) (Supplementary Table 3). The model used to predict the seroprevalence explained 57.8% of the variance. Figure 1 presents the global distribution of IgM seroprevalence in each country.

**Table 2:** Meta-regressions to identify associations and sources of between-study heterogeneity of *Toxoplasma gondii* seroprevalence in pregnant women.

	Studies (n)	Univariable analysis			Variance explained R^2^ (%)	Multivariable analysis		
		Prevalence OR (95%CI)	*p* value	*p* value , moderator		Adjusted prevalence OR (95%CI)	*p* value	*p* value , LR test
**IgM seroprevalence**								
Country level of income				0.0006	12.5			< 0.0001
High	38	1.00 (ref)				1.00 (ref)		
Upper-middle	96	1.01 (0.98–1.04)	0.523			1.01 (0.98–1.05)	0.503	
Lower-middle	25	1.10 (1.05–1.15)	0.0001			1.10 (1.04–1.16)	0.002	
Low	14	1.04 (0.98–1.10)	0.194			1.02 (0.95–1.10)	0.550	
WHO regions				< 0.0001	21.8			< 0.0001
Africa	22	1.00 (ref)				1.00 (ref)		
Americas	37	0.94 (0.90–0.98)	0.009			0.98 (0.91–1.04)	0.486	
Eastern Mediterranean	36	1.03 (0.99–1.08)	0.163			1.06 (1.00–1.12)	0.067	
Europe	39	0.94 (0.90–0.99)	0.010			0.98 (0.92–1.05)	0.583	
South-East Asia	17	0.97 (0.92–1.03)	0.339			0.96 (0.90–1.02)	0.201	
Western Pacific	22	0.96 (0.91–1.01)	0.087			0.99 (0.92–1.06)	0.727	
Year of study (increased by 10 years)	173	1.02 (1.0008–1.03)	0.041	0.041	21.9	1.01 (0.99–1.02)	0.479	0.479
**IgG seroprevalence**								
Country level of income				0.004	6.5			< 0.0001
High	59	1.00 (ref)				1.00 (ref)		
Upper-middle	120	1.14 (1.06–1.22)	0.0006			1.12 (1.05–1.21)	0.002	
Lower-middle	34	1.07 (0.97–1.18)	0.177			1.10 (0.98–1.24)	0.102	
Low	21	1.15 (1.03–1.29)	0.015			1.14 (0.97–1.32)	0.098	
WHO regions				< 0.0001	7.1			< 0.0001
Africa	35	1.00 (ref)				1.00 (ref)		
Americas	47	1.09 (0.997–1.20)	0.058			1.10 (0.96–1.26)	0.195	
Eastern Mediterranean	46	1.03 (0.94–1.13)	0.491			1.06 (0.94–1.20)	0.326	
Europe	63	0.93 (0.86–1.02)	0.114			1.00 (0.87–1.14)	0.930	
South-East Asia	17	0.88 (0.78–0.99)	0.037			0.89 (0.78–1.02)	0.088	
Western Pacific	26	0.74 (0.66–0.82)	< 0.0001			0.76 (0.66–0.87)	0.0001	
Year of study (increased by 10 years)	234	1.02 (0.98–1.06)	0.407	0.407	17.8			

**Figure 2:**
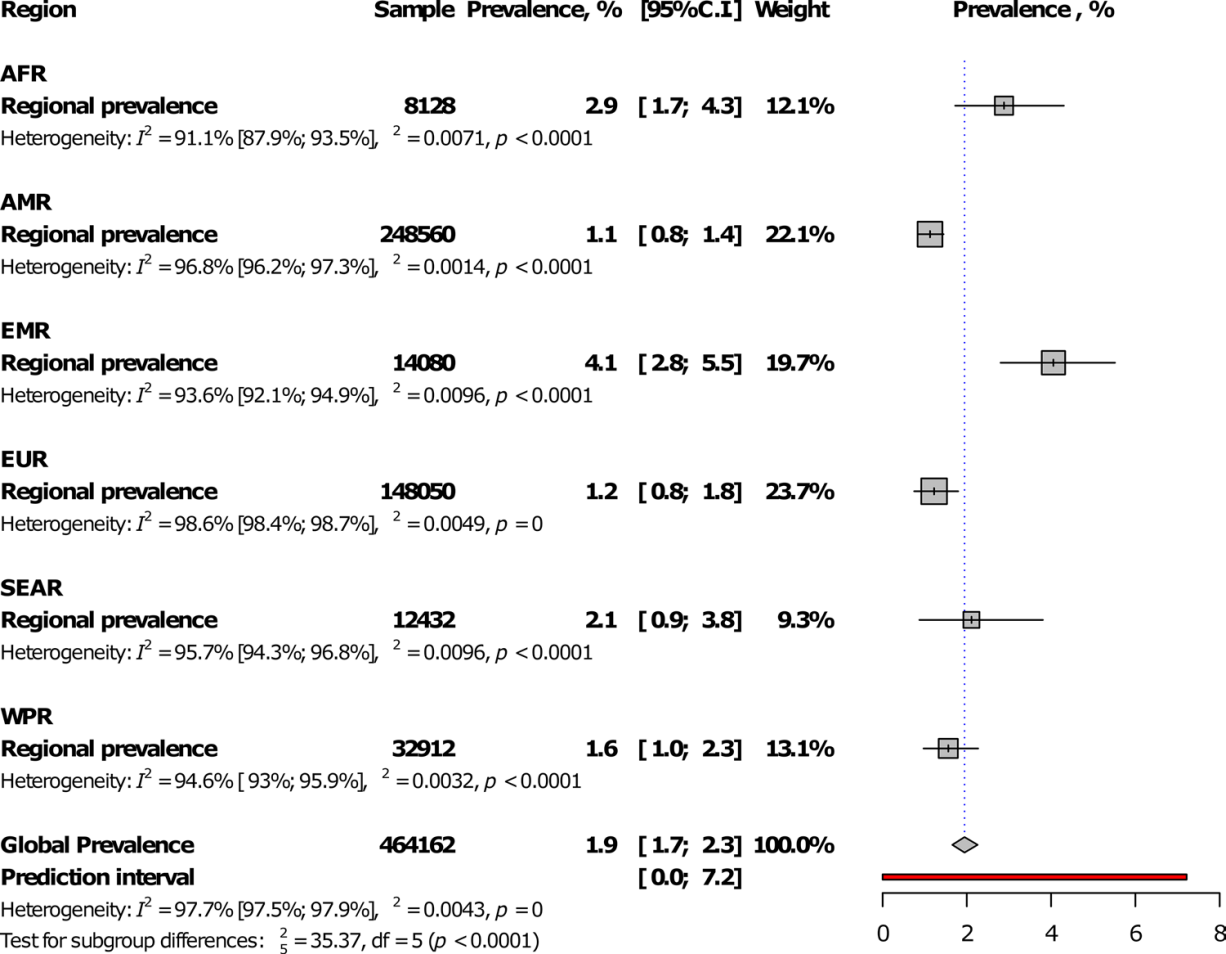
Meta-analysis prevalence of *Toxoplasma gondii* immunoglobulins M seroprevalence in pregnant women by WHO regions.

**Figure 3:**
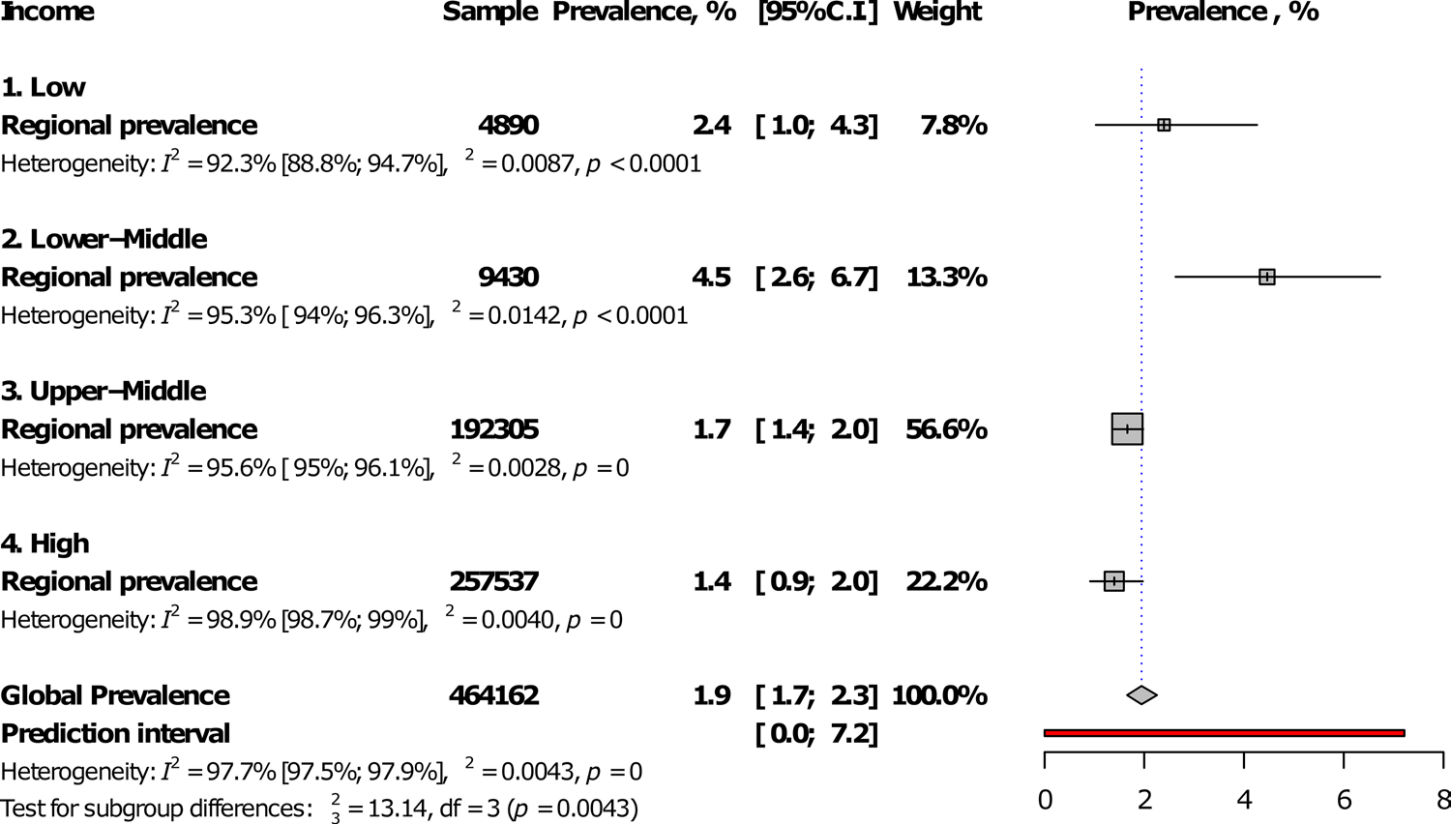
Meta-analysis prevalence of *Toxoplasma gondii* immunoglobulins M seroprevalence in pregnant women by country level of income.

At the regional level, Eastern Mediterranean had the highest IgM seroprevalence (4.1%, 95%CI: 2.8–5.5) and The Americas, the lowest (1.1%, 0.8–1.4), with a statistically significant difference between WHO regions, *p* < 0.0001 (Fig. 2; Table 1; Supplementary Figs. 2–7). The prevalence was higher in low- and low-middle income countries compared to upper-middle and high-income countries, *p* = 0.004 (Fig. 3; Table 1). In addition, the prevalence was higher in studies conducted after 2000 in comparison to studies conducted before, *p* < 0.0001 (Table 1). The global IgM seroprevalence was 1.9% (95%CI: 1.7–2.3) with substantial heterogeneity (Table 1). The funnel plot (Supplementary Fig. 8) suggested a publication bias confirmed by the formal Egger test (Table 1). The prevalence including only low risk of bias studies was close to that of crude analysis (Table 1). In the three-level hierarchical (WHO/Country/Study) meta-analysis, the global IgM seroprevalence was 2.1% (95%CI: 0.6–4.3) with the hierarchical variable (WHO/Country) explaining 61.6% of the variance not attributable to sampling error.

In the univariable meta-regression analysis, IgM seroprevalence was associated with country-level of income (*p* = 0.0006, R^2^: 12.5%), WHO regions (*p* < 0.0001, R^2^; 21.8%), and year of study (*p* = 0.041, R^2^: 21.9%) (Table 2). In the multivariable model, IgM seroprevalence was higher in lower-middle countries compared to others. Variables in the multivariable model explained 27.5% of the total variance of the IgM seroprevalence.

### IgG seroprevalence of *Toxoplasma gondii* among pregnant women

**Figure 4:**
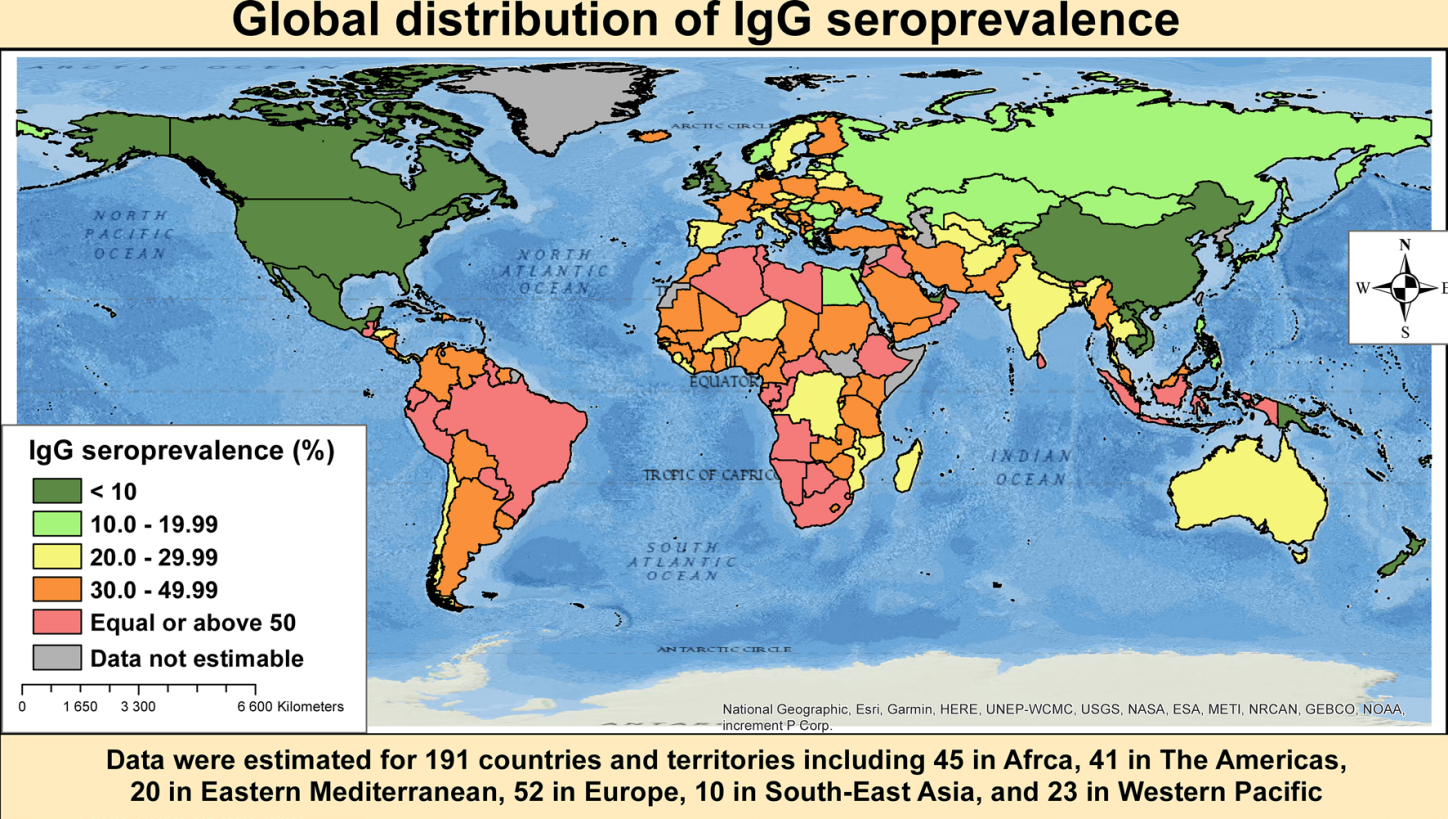
Global distribution of *Toxoplasma gondii* immunoglobulins G seroprevalence in pregnant women.

We were able to estimate 26 country prevalence estimates based on empirical data. Based on empirical data, the three countries with the highest IgG seroprevalence were Ethiopia (64.2%, 95%CI: 34.3–89.1), Gabon (56.7%, 54.4–59.0), and Brazil (53.8%, 39.3–68.0) whereas the three lowest were Mexico (7.2%, 5.3–9.4), South Korea (2.1%, 0.6–4.3), and Canada (0.2%, 0.2–0.3) (Supplementary Table 4). When considering predicted prevalence, the first 15 countries with the highest prevalence were from WHO Africa and Eastern Mediterranean. The five highest predicted prevalence were from Namibia (74.3%, 95%CrI: 73.9–74.7), Eswatini (72.2, 71.7–72.6), Bahrain (71.5, 70.6–72.5), South Africa (70.1, 69.6–70.6), and Algeria (67.5, 67.0–68.1) (Supplementary Table 3). The model used to predict seroprevalence explained 69.7% of the variance. Figure 4 presents the global distribution of IgG seroprevalence.

**Figure 5:**
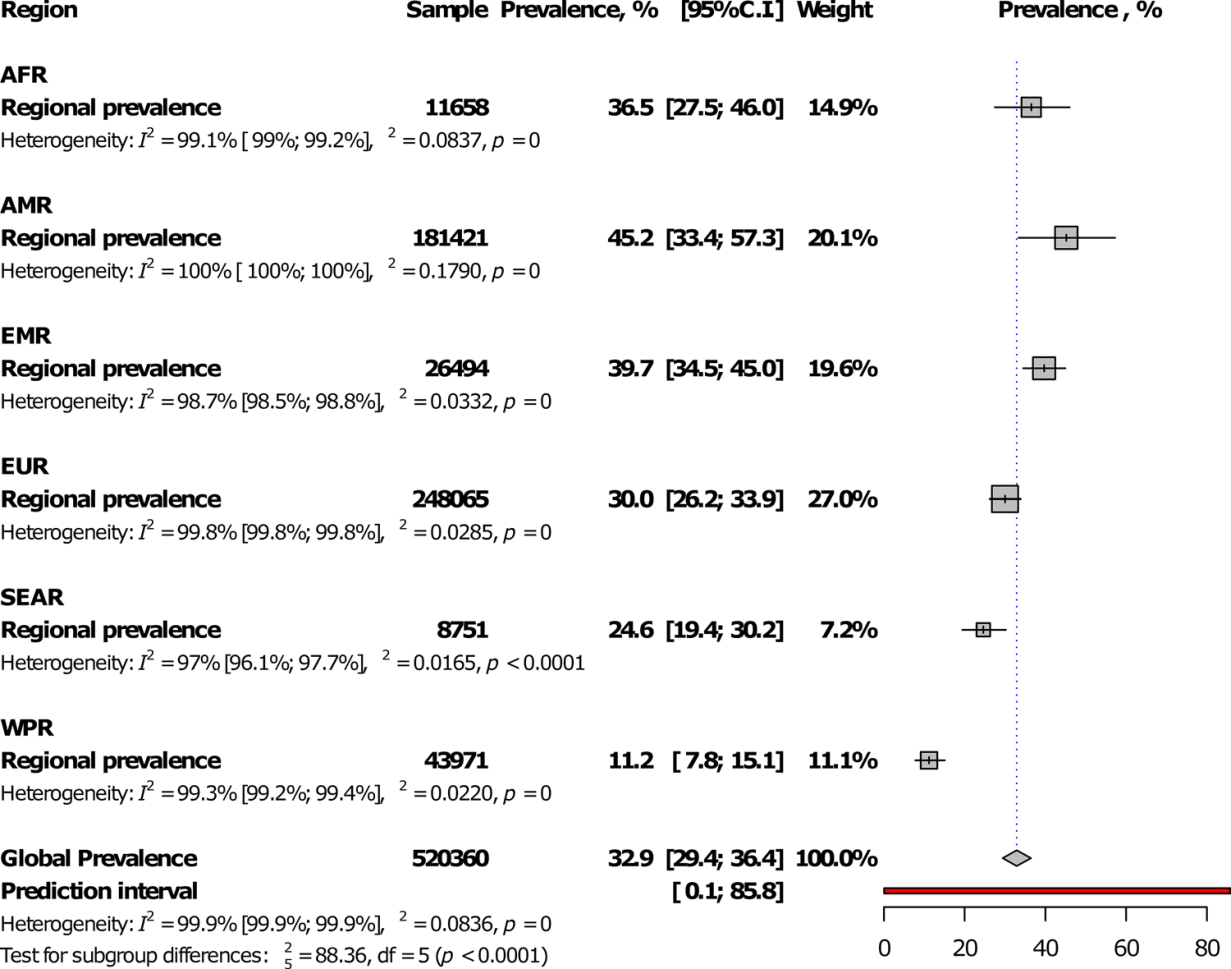
Meta-analysis prevalence of *Toxoplasma gondii* immunoglobulins G seroprevalence in pregnant women by WHO regions.

**Figure 6:**
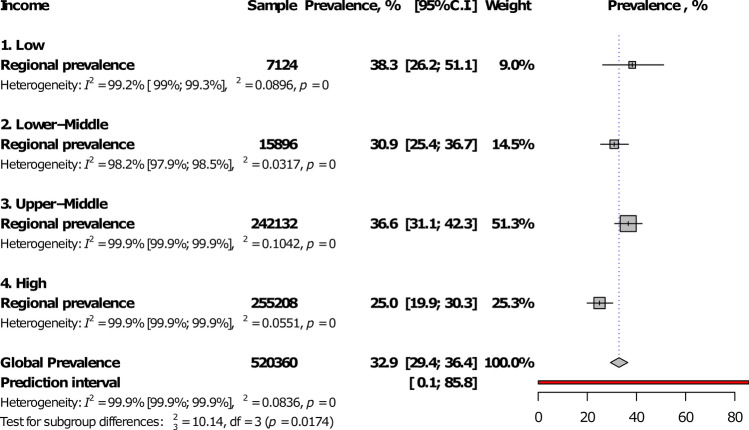
Meta-analysis prevalence of *Toxoplasma gondii* immunoglobulins G seroprevalence in pregnant women by country level of income.

Among WHO regions, The Americas had the highest prevalence (45.2%, 95%CI: 33.4–53.4) and Western Pacific the lowest (11.2%, 7.8–15.1), with a statistically significant difference between regions, *p* < 0.0001 (Fig. 5; Table 1; Supplementary Figs. 9–14). The prevalence was lower in high-income countries compared to others, *p* = 0.017 (Fig. 6; Table 1). There was no difference between studies conducted before and after 2000, p (Table 1). The global IgG seroprevalence was 32.9% (95%CI: 29.4–36.4) with substantial heterogeneity (Table 1). The funnel plot (Supplementary Fig. 15) suggested the publication bias confirmed by the formal Egger test (Table 1). The prevalence including only low risk of bias studies was close to that of crude analysis (Table 1). In the three-level hierarchical (WHO/Country/Study) meta-analysis, the global IgG seroprevalence was 27.7% (95%CI: 20.7–35.4) with the hierarchical variable (WHO/Country) explaining 83.0% of the variance not attributable to sampling error.

In the univariable meta-regression analysis, IgG seroprevalence was associated with country-level of income (*p* = 0.004, R^2^: 6.5%), WHO regions (*p* < 0.0001, R^2^; 7.1%), but not with year of study (*p* = 0.407, R^2^: 17.8%) (Table 2). In the multivariable model, IgG seroprevalence was higher in upper-middle countries and lower in WHO Western Pacific compared to others. Variables in the multivariable model explained 21.7% of the total variance of the IgG seroprevalence (Table 2).

## Discussion

This systematic review with meta- and modelling analysis of 250 prevalence data including 723,655 pregnant women revealed a relative lower global IgM seroprevalence of *Toxoplasma gondii*, at 1.9% (95%CI: 1.7–2.3), but a much higher IgG seroprevalence, at 32.9% (95%CI: 29.4–36.4). However, there was substantial statistical heterogeneity between included studies, along with publication bias. When considering the predicted prevalence estimates, the first 17/15 countries with the highest IgM/IgG seroprevalence of *Toxoplasma gondii* originated from the WHO Eastern Mediterranean and Africa regions. Moreover, the IgM seroprevalence was highest in Eastern Mediterranean followed by Africa, and lowest in the Americas. The IgG seroprevalence was highest in the Americas followed by Eastern Mediterranean and lowest in Western Pacific. Further, there was a substantial statistical difference in seroprevalence estimates between WHO regions, for both immunoglobulins. In addition, the seroprevalence was the lowest in high-income countries compared to others, with a statistical difference between different levels of income.

The global and regional IgG seroprevalence estimates of *Toxoplasma gondii* derived from this study tend to mirror trends in the general population as in the study published by Rostami and colleagues^7^. In fact, seroprevalence estimates in the adult population were estimated at 20–40% in the UK and USA, 50–80% in Central Europe, South and Central America, and in West Africa, 4–39% in South East Asia, China, and Korea, 11–28% in Scandinavia, and 30% in Australia^18^. Furthermore, results from a systematic review on the global status of *Toxoplasma gondii* seroprevalence showed that global seropositivity rates ranged from less than 10% to over 90%, with foci of high prevalence being recorded in Latin America, parts of Eastern & Central Europe, the Middle East, parts of South East Asia and Africa^19^.

Trends in *Toxoplasma gondii* seroprevalence over time, both in the general population and in pregnant women seemed to have broadly decreased over time in western countries^19^, casts doubting with an upward trend observed in some other countries like China^20^. Contrasting with both observations, results from the present review tend to show that globally, IgM or IgG seroprevalence estimates have not evolved over time, as time of publication did not have any influence on these seroprevalence rates. Further research is warranted to deeply address this issue.

The distribution of IgM and IgG seroprevalence estimates yielded huge discrepancies between various countries and regions of the world, which was already pointed out in previous reports showing even variations within the same country^4,6–8,19,20^. Indeed, variations in seroprevalence of *Toxoplasma gondii* tend to be correlated with the dietary and hygiene habits of a population, particularly related to some major risk factors for *Toxoplasma gondii* infection which include eating raw or undercooked meat, unwashed raw vegetables or fruits, and contact with cats. Furthermore, it has been demonstrated that *Toxoplasma gondii* seroprevalence is lower in cold regions, hot and arid areas, or at high altitudes^4,20^.

Moreover, living in rural areas and low educational level have also been incriminated as increasing *Toxoplasma gondii* seropositivity. Previous reports depicted higher levels of *Toxoplasma gondii* seroprevalence in rural or suburban regions than in urban ones. In general, rural residence is associated with poorer sanitary conditions, more frequent contact with soil or animals and drinking unpotable water^19,20^. Additionally and because of its lower socioeconomic status (in general or compared to the urban population of a given country), a rural population may have worse access to healthcare and may further exhibit a lower level of health literacy^4^. This can clearly explain why women of low educational levels in China were more likely to acquire *Toxoplasma gondii* infection during their pregnancy or showed higher seroprevalence than those with higher education, suggesting, therefore, health education as an effective intervention towards curbing *Toxoplasma gondii* infection in pregnancy^20^. Unsurprisingly, IgM and IgG seroprevalence rates were highest in low-income countries, with the 17/15 highest predicted rates found in developing countries of East Mediterranean and Africa regions, where the incidence and burden of congenital toxoplasmosis are also the highest^6^.

Actually, the dangerousness of *Toxoplasma gondii* infection during pregnancy is related to the transplacental transmission which can cause miscarriage, stillborn foetuses, neonatal death, or foetal/neonatal abnormalities. It has been claimed that this vertical transmission occurs mainly when the infection is acquired for the first time during pregnancy, with the risk of transmission rising steeply with gestational age: around 15% in the first trimester, 25% in the second one and 65% or more in the third trimester. The situation worsens in case of immunodeficiency including HIV/AIDS^21,22^.

For clinical diagnosis, the seropositivity of IgM is interpreted as a recent or acute infection^4,20^. Therefore, the present results indicate that almost 2% of women might present an acute *Toxoplasma gondii* infection during their pregnancy with possible subsequent complications including congenital toxoplasmosis. However and considering that IgM can sometimes persist for years hence giving rise to false-positive cases of acute infection, a combination of two of the following criteria should be indicative of an acute *Toxoplasma gondii* infection: (i) specific IgG positivity and a significant increase in its titres (≥ fourfold) in 2 weeks; (ii) specific IgM positivity, and (iii) detection of circulating antigens^20^. Although this paper did not focus specifically on the prevalence of acute *Toxoplasma gondii* infection during pregnancy, it was observed different ways of diagnosing acute *Toxoplasma gondii* infection during pregnancy, thus highlighting the lack of current consensus on this point which needs to be addressed shortly soon.

The lack of consensus extends to preventive public health interventions to reduce the risk of *Toxoplasma gondii* infection during pregnancy and potentially consequential congenital toxoplasmosis. The best strategy to prevent and control maternal and subsequent neonatal toxoplasmosis remains unclear: many interventions have been tested in different contexts. For instance, some authors have recommended health education alone, while in certain countries prenatal screening and treatment are carried out^4,18,20,21^.

However, it was shown that in areas with low seroprevalence rates there was a low risk of maternofoetal transmission in women with untreated *Toxoplasma gondii* infection (comparable with that from treated women) as well as a low risk of substantial clinical signs in children with congenital toxoplasmosis born from untreated mothers. Hence in such areas, prenatal screening and treatment may not be justified^22^. Furthermore, uncertainty about the overall benefits of this strategy to be balanced against adverse treatment effects and the infrastructure and costs needed for its implementation have led to the design of other policies including neonatal screening^4,22,23^. Accordingly, it has been claimed that a neonatal screening programme based on detection of toxoplasma-specific IgM antibodies alone could identify around 70–80% of cases of congenital toxoplasmosis^4,22^. Definitely, there is need for large randomized controlled trials and cost–benefit analyses to clearly point out the best strategy to prevent maternal and congenital toxoplasmosis.

This study should however be interpreted in the context of some drawbacks. First and very common to most meta-analyses of prevalence studies, substantial heterogeneity was found between studies. The meta-regression permitted to identify potential sources of heterogeneity, even if the variance explained was < 30%, including the country’s WHO region and level of income, and the time of publication at a certain extent. Additionally, and even though not ascertained, the method of laboratory investigations/diagnostic tests used in various studies might have contributed to this heterogeneity. However, and bearing in mind that avoiding statistical heterogeneity in meta-analyses of prevalence data is practically impossible^24^, we applied rigorous selection criteria to make sure that all studies were similar enough to be pooled together. Second, various regions of the world were disproportionally represented; very few studies were national representatives, had a high number of participants and used a random sampling method. Consequently, the translatability of results at a global scale might be jeopardized, perhaps reinforced by existence of publication bias. To reduce these risks to their lesser extent, the study inclusion process was independently conducted by independent investigators on the basis of a consensual and published protocol^9^. Besides, 14 major databases were extensively searched, complemented by manually searching the reference list of pertinent publications. Third, all studies were hospital-based; hence, this may not reflect the true burden of *Toxoplasma gondii* infection in the general population of pregnant women, especially when considering that antenatal care coverage and skilled birth attendance for pregnant women are sub-optimal^25,26^. Lastly, some countries did not have empirical data, for which a Bayesian generalized non-linear multilevel model was used to predict their respective prevalence estimates. But this model explained less than 75% of the variance observed.

Notwithstanding and to the best of our knowledge, this is the first contemporary and comprehensive summary of the global, regional and countries’ IgM and IgG seroprevalence estimates of *Toxoplasma gondii* infection during pregnancy. A protocol was published before the study was initiated^9^. In the end, 250 studies compiling over 730,000 participants were included as the result of a methodological process rigorously conducted in line with the published protocol. Furthermore, robust statistical procedures were applied including sub-group and modelling analyses, which permitted to generate prevalence estimates for 191 countries and WHO regions. More still, sensitivity analyses including only studies with a low risk of bias and the three-level meta-analysis taking in account variance between countries and WHO regions in the hierarchy yielded similar results than that for crude analyses, indicative of the truthfulness to be accorded to these crude findings.

This systematic review with meta- and modelling analyses has depicted the global, regional and countries’ IgM and IgG seroprevalence estimates of *Toxoplasma gondii* infection in pregnant women, putting in light very high trends at different levels and huge disparities between countries in accordance with their geographical location and level of income. Countries of high seroprevalence rates have been identified, where the risk of maternal and congenital toxoplasmosis is the highest, hence, deserving more attention for preventive interventions. In the absence of current consensual recommendations, the strategy to be adopted should be tailored to each country’s data, level of income and robustness of health system. For instance, educating pregnant women and their health care providers on manifestations, risks and consequences, preventive and treatment measures against *Toxoplasma gondii* infection appears as the most accessible and cost-effective strategy especially in low-resource environments where large population screening programmes would be direly expensive and unaffordable. However, once prevention fails and acute infection occurs during pregnancy, treatment should be discussed to reduce the risk of vertical transmission and/or clinical severity of foetal infection. Notwithstanding, further research in this regard is needed to guide consensual attitudes towards the prevention, detection and management of *Toxoplasma gondii* infection in pregnancy.

## Supplementary information

Supplementary Information.

## Data Availability

All data generated or analysed during this study are included in this published article and its Supplementary Information files.
