# Supplementary material for: Global, regional, and country seroprevalence of Toxoplasma gondii in pregnant women: a systematic review, modelling and meta-analysis
Source: Sci Rep. 2020 Jul 21;10:12102. doi: 10.1038/s41598-020-69078-9 (PMC7374101; doi:10.1038/s41598-020-69078-9)
Supplement: Supplementary file 1 — Supplementary Information. [file 41598_2020_69078_MOESM1_ESM.pdf]

Global, regional, and country seroprevalence of *Toxoplasma gondii* in pregnant women: a systematic review, modelling and meta-analysis

Jean Joel **Bigna**, Joel Noutakdie **Tochie**, Dahlia Noelle **Tounouga**, Anne Olive **Bekolo**, Nadia S. **Ymele**, Emilie Laettitia **Youda**, Paule Sandra **Sime**, Jobert Richie **Nansseu**

APPENDIX

Supplementary Tables

Supplementary Table 1. Characteristics of included studies 3
Supplementary Table 2. Meta-analysis IgM seroprevalence of Toxoplasma gondii in the population of pregnant women, by country 12
Supplementary Table 3. Predicted seroprevalence of Toxoplasma gondii in pregnant women in each county in different WHO regions 13
Supplementary Table 3. Meta-analysis IgG seroprevalence of Toxoplasma gondii in the population of pregnant women, by country 17

Supplementary Figures

Supplementary Figure 1. Process of identification and selection of studies for inclusion in the review (PRISMA flow diagram) 18
Supplementary Figure 2. Meta-analysis of IgM seroprevalence of Toxoplasma gondii among pregnant women in WHO Africa 19
Supplementary Figure 3. Meta-analysis of IgM seroprevalence of Toxoplasma gondii among pregnant women in WHO Americas 20
Supplementary Figure 4. Meta-analysis of IgM seroprevalence of Toxoplasma gondii among pregnant women in WHO Eastern Mediterranean 21
Supplementary Figure 5. Meta-analysis of IgM seroprevalence of Toxoplasma gondii among pregnant women in WHO Europe 22
Supplementary Figure 6. Meta-analysis of IgM seroprevalence of Toxoplasma gondii among pregnant women in WHO South-East Asia 23
Supplementary Figure 7. Meta-analysis of IgM seroprevalence of Toxoplasma gondii among pregnant women in Western Pacific 24
Supplementary Figure 8. Funnel plot for meta-analysis of IgM seroprevalence of Toxoplasma gondii in the global population of pregnant women 25
Supplementary Figure 9. Meta-analysis of IgG seroprevalence of Toxoplasma gondii among pregnant women in WHO Africa 26
Supplementary Figure 10. Meta-analysis of IgG seroprevalence of Toxoplasma gondii among pregnant women in WHO Americas 27
Supplementary Figure 11. Meta-analysis of IgG seroprevalence of Toxoplasma gondii among pregnant women in WHO Eastern Mediterranean 28

Supplementary Figure 12. Meta-analysis of IgG seroprevalence of Toxoplasma gondii among pregnant women in WHO Europe 29
Supplementary Figure 13. Meta-analysis of IgG seroprevalence of Toxoplasma gondii among pregnant women in WHO South-East Asia 30
Supplementary Figure 14. Meta-analysis of IgG seroprevalence of Toxoplasma gondii among pregnant women in Western Pacific 31
Supplementary Figure 15. Funnel plot for meta-analysis of IgG seroprevalence of Toxoplasma gondii in the global population of pregnant women 32

Reference Lists of Included Studies……….33

Supplementary Table 1. Characteristics of included studies

Author	Year	Bias	Study year(s)	Country	Setting	Sites	Sample
**Abamecha**	2016	Low	2014-2015	Ethiopia	Antenatal Care Unit	One site	232
**Abazaj**	2016	Moderate	NR	Albania	Antenatal Care Unit	One site	152
**Abdallah**	2013	Moderate	2007-2010	Tunisia	Hospital-based (but not ANC)	One site	2070
**Abdi**	2008	Low	2008	Iran	Hospital-based (but not ANC)	One site	533
**Abolghasem**	2011	Moderate	NR	Iran	Hospital-based (but not ANC)	One site	225
**Ahlfors**	1989	Low	1982-1983	Sweden	Population-based	Multi-site	4351
**Ahmadpour**	2015	Moderate	2012-2013	Iran	Antenatal Care Unit	One site	264
**Akhlaghi**	2016	Low	2014	Iran	Antenatal Care Unit	One site	468
**Akinbami**	2010	Moderate	NR	Nigeria	Hospital-based (but not ANC)	One site	179
**Al-Eryani**	2016	Moderate	2010-2011	Yemen	Antenatal Care Unit	One site	593
**Ali**	2017	Low	2016-2017	Iraq	Unclear/No description	One site	161
**Allain**	1998	Low	1992	UK	Antenatal Care Unit	Multi-site	13328
**Almogren**	2011	Low	2009-2010	Saudi Arabia	Antenatal Care Unit	One site	2176
**Almushait**	2014	Moderate	2008-2010	Saudi Arabia	Antenatal Care Unit	Multi-site	487
**Alvarado**	2006	Low	2005-2006	Mexico	Antenatal Care Unit	One site	343
**Alvarado-Esquivel**	2009	Low	2007-2008	Mexico	Antenatal Care Unit	Multi-site	439
**Amar**	2015	High	2012-2013	India	Antenatal Care Unit	One site	103
**Andiappan**	2014	Low	2012-2013	Thailand	Antenatal Care Unit	One site	760
**Angel-Nueller**	2011	Low	2009-2010	Colombia	Hospital-based (but not ANC)	Multi-site	2258
**Aqueely**	2014	Moderate	2013	Saudi Arabia	Antenatal Care Unit	One site	195
**Ashrafunnessa**	1998	Moderate	NR	Bangladesh	Antenatal Care Unit	One site	286
**Asthana**	2006	Low	1995	Grenada	Antenatal Care Unit	One site	534
**Avelar**	2017	Low	2014	Brazil	Antenatal Care Unit	One site	712
**Awake**	2015	Low	2013-2014	Ethiopia	Antenatal Care Unit	One site	384
**Ayi**	2016	Moderate	NR	Ghana	Antenatal Care Unit	One site	125
**Aynioglu**	2015	Low	2012-2014	Turkey	Hospital-based (but not ANC)	One site	910
**Aysun**	2011	Low	2008-2009	Turkey	Hospital-based (but not ANC)	One site	1102

**Babaie**	2013	Moderate	2010-2011	Iran	Antenatal Care Unit	One site	419
**Bamba**	2017	Low	2013-2014	Burkina Faso	Antenatal Care Unit	One site	316
**Bari**	1990	Moderate	NR	Pakistan	Hospital-based (but not ANC)	Multi-site	324
**Barrera**	2002	Moderate	NR	Colombia	Antenatal Care Unit	One site	301
**Beach**	1979	Low	NR	USA	Hospital-based (but not ANC)	Multi-site	95929
**Beredjem**	2017	Low	2017	Algeria	Hospital-based (but not ANC)	One site	143
**Beytur**	2010	Moderate	2008-2009	Turkey	Hospital-based (but not ANC)	One site	205
**Bin**	2012	Low	2009-2010	Saudi Arabia	Hospital-based (but not ANC)	Multi-site	137
**Bittencourt**	2012	Moderate	NR	Brazil	Unclear/No description	Multi-site	4021
**Boa-Sorte**	2014	Low	2009-2010	Brazil	Antenatal Care Unit	One site	692
**Borkakoty**	2007	Low	2003-2004	India	Antenatal Care Unit	One site	180
**Borkakoty**	2016	Low	2007-2009	India	Antenatal Care Unit	One site	1141
**Boronina**	2018	High	NR	Russia	Antenatal Care Unit	One site	65
**Buchy**	2003	Moderate	NR	Vietnam	Hospital-based (but not ANC)	One site	300
**Capretti**	2014	Low	2009-2011	Italy	Antenatal Care Unit	One site	10867
**Cardenas**	2011	Low	2004	Colombia	Antenatal Care Unit	One site	167
**Çekin**	2011	Moderate	2008-2011	Turkey	Antenatal Care Unit	One site	7520
**Cetin**	2017	Low	2009-2016	Turkey	Hospital-based (but not ANC)	One site	11564
**Chandrenasa**	2016	Low	2014	Sri Lanka	Hospital-based (but not ANC)	One site	293
**Chen**	2014	Moderate	NR	China	Unclear/No description	One site	2993
**Chintana**	1998	Moderate	NR	Thailand	Unclear/No description	NR	1181
**Chou**	2011	Moderate	NR	China	Antenatal Care Unit	Multi-site	200
**Cinara**	2011	Low	2005-2007	Brazil	Hospital-based (but not ANC)	One site	87
**Cong**	2015	Low	2011-2013	China	Antenatal Care Unit	National	965
**Contreras**	1995	Moderate	NR	Chile	Unclear/No description	One site	139
**Costa**	2018	Low	2009-2010	Brazil	Antenatal Care Unit	One site	726
**Da Rocha**	2015	Low	NR	Brazil	Hospital-based (but not ANC)	One site	338
**Dasilva**	2014	Low	2012-2013	Brazil	Antenatal Care Unit	One site	487
**De Moura**	2013	Low	2010-2011	Brazil	Hospital-based (but not ANC)	One site	400
**De Noya**	2010	Moderate	NR	Venezuela	Hospital-based (but not ANC)	One site	678

**De Paschale**	2014	Moderate	2011	Benin	Unclear/No description	One site	283
**De Quadros**	2015	Low	2008-2009	Brazil	Hospital-based (but not ANC)	Multi-site	148
**Dentico**	2011	Low	2005	Italy	Antenatal Care Unit	One site	334
**Depaschale**	2008	Low	2004-2005	Italy	Population-based	One site	3426
**Dias**	2011	Low	2007-2008	Brazil	Antenatal Care Unit	Multi-site	607
**Diaz-Suarez**	2009	Low	NR	Venezuela	Hospital-based (but not ANC)	One site	100
**Doehring**	1995	Low	1989-1991	Tanzania	Hospital-based (but not ANC)	One site	849
**Dogan**	2014	Low	2008-2013	Turkey	Hospital-based (but not ANC)	One site	1709
**Doudou**	2014	Low	2011	Congo	Antenatal Care Unit	Multi-site	781
**Durdu**	2017	Low	2006	Turkey	Antenatal Care Unit	One site	102
**Eldeeb**	2012	Low	2010-2011	Egypt	Antenatal Care Unit	One site	323
**Eliakimu**	2018	Moderate	2017	Tanzania	Antenatal Care Unit	One site	254
**Elnahas**	2003	Moderate	2000	Sudan	Antenatal Care Unit	Multi-site	487
**El-Nawawy**	1996	Moderate	1992-1993	Egypt	Hospital-based (but not ANC)	One site	150
**Elsafi**	2015	Low	2012-2013	Saudi Arabia	Antenatal Care Unit	One site	400
**Emelia**	2014	Moderate	2012-2013	Malaysia	Unclear/No description	One site	281
**Endris**	2014	Low	2010-2011	Ethiopia	Antenatal Care Unit	One site	385
**Ertug**	2005	Low	2004	Turkey	Antenatal Care Unit	One site	389
**Esboei**	2016	Low	2013-2014	Iran	Antenatal Care Unit	One site	786
**Evengard**	1999	Moderate	1992-1993	Sweden	Unclear/No description	NR	3094
**Ferezin**	2013	Low	2010	Brazil	Antenatal Care Unit	One site	1534
**Figueiró-Filho**	2007	Low	2002-2003	Brazil	Antenatal Care Unit	One site	32512
**Flatt**	2013	Low	2006-2008	UK	Antenatal Care Unit	One site	2610
**Fochi**	2015	Low	2005-2007	Brazil	Antenatal Care Unit	One site	213
**Fonseca**	2012	Low	2007	Brazil	Hospital-based (but not ANC)	Multi-site	2136
**Franck**	2008	Moderate	NR	France	Hospital-based (but not ANC)	One site	200
**Franklin**	1993	Moderate	NR	Israel	Hospital-based (but not ANC)	One site	213
**Freise**	1993	Low	NR	Germany	Hospital-based (but not ANC)	One site	512

**Freitas**	2017	Low	2009-2014	Brazil	Antenatal Care Unit	One site	356
**Frimpong**	2017	Low	2015	Zambia	Antenatal Care Unit	One site	411
**Fusun**	2011	Moderate	2000-2009	Turkey	Antenatal Care Unit	One site	1646
**Gamba**	2013	Low	2011-2012	Central African Republic	Antenatal Care Unit	National	434
**Gao**	2017	Low	NR	China	Unclear/No description	One site	404
**Garedaghi**	2017	Moderate	2014	Iran	Population-based	One site	200
**Gebremedhin**	2013	Low	2011	Ethiopia	Hospital-based (but not ANC)	Multi-site	213
**Gencer**	2014	Low	2012_2013	Turkey	Antenatal Care Unit	One site	196
**Gilbert**	1992	Low	1980-1986	UK	Antenatal Care Unit	Multi-site	6749
**Gonçalves**	2010	Moderate	2006-2007	Brazil	Antenatal Care Unit	One site	574
**Guerra**	2014	Low	2007	Brazil	Antenatal Care Unit	One site	2673
**Guerra**	1995	Moderate	1993	Spain	Antenatal Care Unit	One site	191
**Gundem**	2014	Low	2013	Turkey	Antenatal Care Unit	One site	419
**Gye-Cheol**	2011	Moderate	NR	South Korea	Unclear/No description	Multi-site	787
**Hajsoleimani**	2012	Low	NR	Iran	Population-based	One site	500
**Han**	2008	Low	2005-2006	South Korea	Antenatal Care Unit	Multi-site	351
**Harma**	2004	Low	NR	Turkey	Antenatal Care Unit	One site	1149
**Hasan**	2017	Low	1994-2015	Lebanon	Antenatal Care Unit	One site	2456
**Hershey**	1987	Moderate	NR	USA	Antenatal Care Unit	One site	120
**Hoseini**	2014	Moderate	NR	Iran	Antenatal Care Unit	Multi-site	289
**Hua**	2013	Moderate	NR	China	Unclear/No description	Multi-site	1081
**Hung**	2015	Moderate	2009-2011	China	Antenatal Care Unit	One site	104
**Iddawela**	2017	Low	2010-2013	Iceland	Antenatal Care Unit	One site	536
**Imam**	2016	Moderate	NR	Saudi Arabia	Antenatal Care Unit	One site	150
**Inagaki**	2009	Moderate	2007	Brazil	Unclear/No description	Multi-site	9550
**Iqbal**	2010	Moderate	2003-2005	India	Antenatal Care Unit	One site	800
**Iqbal**	2007	Moderate	2002-2005	Kuwait	Antenatal Care Unit	One site	224

**Isabelle Ribeiro**	2009	Moderate	2007	Brazil	Hospital-based (but not ANC)	One site	190
**Ismail**	2014	Moderate	2012-2013	Saudi Arabia	Unclear/No description	NR	96
**Jacquier**	1995	Low	1990-1991	Switzerland	Population-based	National	9059
**Jaqueti**	1991	Moderate	NR	Spain	Unclear/No description	NR	1221
**Jawahir**	2016	Moderate	2011	Saudi Arabia	Antenatal Care Unit	Multi-site	250
**Jemal**	2018	Low	2015	Ethiopia	Antenatal Care Unit	One site	401
**Jenum**	1998	Low	1992-1994	Norway	Antenatal Care Unit	Multi-site	35940
**Jiang**	2018	Low	2016-2017	China	Antenatal Care Unit	One site	313
**Josheghani**	2015	Moderate	2010-2012	Iran	Antenatal Care Unit	One site	80
**Jumaian**	2005	Low	2000-2001	Jordan	Antenatal Care Unit	One site	280
**Kamal**	2015	Moderate	2013-2014	Egypt	Antenatal Care Unit	One site	240
**Karacan**	2014	Low	2009-2013	Turkey	Antenatal Care Unit	One site	1258
**Karunajeewa**	2001	Moderate	1996-1999	Australia	Antenatal Care Unit	One site	308
**Kaur**	1999	Moderate	NR	India	Antenatal Care Unit	One site	120
**Khurana**	2010	Low	2005-2006	India	Antenatal Care Unit (ANC), Population-based	Multi-site	300
**Klufio**	1993	Moderate	NR	Papua New Guinea	Antenatal Care Unit	One site	197
**Laboud**	2017	Low	2015-2016	Morocco	Antenatal Care Unit	One site	128
**Lebech**	1993	Moderate	NR	Denmark	Unclear/No description	Multi-site	5402
**Leone**	1996	Moderate	1993-1994	Italy	Hospital-based (but not ANC)	One site	1668
**Lin**	2008	Low	NR	China	Antenatal Care Unit (ANC), Hospital-based (but not ANC)	Multi-site	426
**Linguissi**	2012	Low	2006-2009	Burkina Faso	Antenatal Care Unit	One site	182
**Lito**	2013	Moderate	2004-2009	Portugal	Antenatal Care Unit	One site	3162
**Liu**	2009	High	NR	China	Unclear/No description	NR	235
**Lobo**	2017	Moderate	2011	Angola	Antenatal Care Unit	One site	300
**Lobo**	2017	Moderate	2010-2011	Portugal	Hospital-based (but not ANC)	One site	155
**Lopes**	2013	Low	2007-2010	Brazil	Antenatal Care Unit	Multi-site	2226

**Lopez**	2009	Low	2006	Brazil	Antenatal Care Unit	One site	492
**Lopez-Fabal**	2013	Low	2007-2010	Spain	Hospital-based (but not ANC)	One site	8012
**Lui**	2014	Moderate	NR	China	Unclear/No description	Multi-site	9139
**Luyasu**	1993	Low	NR	Belgium	Hospital-based (but not ANC)	One site	830
**Maggi**	2008	Low	2004-2005	Italy	Hospital-based (but not ANC)	Multi-site	496
**Maha**	2012	Low	2011	Sudan	Antenatal Care Unit	One site	188
**Mahdy**	2017	Moderate	2012-2014	Yemen	Population-based	Multi-site	359
**Maija**	1992	Low	1988-1989	Finland	Unclear/No description	Multi-site	16733
**Majid**	2016	Low	2015	Pakistan	Antenatal Care Unit	One site	733
**Mansouri**	2007	Moderate	NR	Morocco	Unclear/No description	NR	2456
**Marcinek**	2008	Low	2000-2007	Poland	Antenatal Care Unit	One site	1800
**Marios**	2016	Low	2009-2011	Cyprus	Population-based	National	17631
**Mendez**	2009	Low	2008	Brazil	Antenatal Care Unit	One site	268
**Mioranza**	2008	Moderate	2005-2006	Brazil	Hospital-based (but not ANC)	One site	334
**Mohamed**	2016	Low	2014	Saudi Arabia	Hospital-based (but not ANC)	One site	326
**Mohammad**	2011	Low	2008-2009	Iran	Hospital-based (but not ANC)	Multi-site	721
**Mohammad**	2013	Low	2009	Saudi Arabia	Antenatal Care Unit	One site	554
**Morris**	2004	Low	2000	New Zealand	Hospital-based (but not ANC)	One site	5000
**Moukandja**	2017	Low	2007-2010	Gabon	Antenatal Care Unit	Multi-site	973
**Mousavi**	2018	Moderate	2016-2017	Iran	Hospital-based (but not ANC)	One site	110
**Mpiga**	2010	Low	2007	Gabon	Hospital-based (but not ANC)	Multi-site	839
**Mumcuoglu**	2014	Moderate	2010-2013	Turkey	Hospital-based (but not ANC)	One site	6140
**Mumtaz**	2017	Low	2014-2016	Turkey	Antenatal Care Unit	One site	7513
**Murebwayire**	2017	Moderate	2014	Rwanda	Antenatal Care Unit	One site	384
**Mwambe**	2013	Low	2012-2013	Tanzania	Antenatal Care Unit	One site	350
**Nabia**	1998	Low	1995-1997	Gabon	Antenatal Care Unit	One site	767
**Naghili**	2017	Low	2010-2013	Iran	Hospital-based (but not ANC)	One site	391
**Nash**	2005	Moderate	1999-2001	UK	Antenatal Care Unit	Multi-site	1897
**Nasir**	2015	Moderate	2014	Nigeria	Antenatal Care Unit	One site	360

**Ndiaye**	2011	Low	2002-2006	Senegal	Hospital-based (but not ANC)	One site	941
**Ndumbe**	1992	Low	1989_1990	Cameroon	Antenatal Care Unit	One site	192
**Niemiec**	2002	Low	2000	Poland	Hospital-based (but not ANC)	One site	2016
**Nigem**	2009	Moderate	2005	Palestine	Antenatal Care Unit	One site	204
**Nissapatom**	2011	Low	2009-2010	Thailand	Antenatal Care Unit	One site	640
**Nissapatorn**	2003	Moderate	2002	Malaysia	Antenatal Care Unit	One site	200
**Njunda**	2011	Moderate	2008	Cameroon	Antenatal Care Unit	One site	110
**Njunda**	2011	Low	2009	Cameroon	Antenatal Care Unit	One site	110
**Nourollahpour**	2016	Low	2014-2015	Iran	Antenatal Care Unit	One site	360
**Nowakowska**	2013	Low	2004-2012	Poland	Antenatal Care Unit	One site	8281
**Nowakowska**	2001	Moderate	1998	Poland	Unclear/No description	NR	1920
**Ocak**	2007	Moderate	2004-2006	Turkey	Unclear/No description	NR	1652
**Okyay**	2013	Low	2007-2012	Turkey	Hospital-based (but not ANC)	One site	3340
**Ouermi**	2009	Low	2009	Burkina Faso	Hospital-based (but not ANC)	One site	276
**Ozekinci**	2005	Low	2002-2003	Turkey	Hospital-based (but not ANC)	One site	879
**Parlak**	2015	Moderate	2012-2013	Turkey	Antenatal Care Unit	One site	9809
**Pucio**	2014	Low	2012	Italy	Hospital-based (but not ANC)	One site	846
**Punda-Polic**	2000	Moderate	1994-1995	Croatia	Hospital-based (but not ANC)	One site	398
**Qin**	2011	Moderate	NR	China	Unclear/No description	NR	1307
**Radoń-Pokracka**	2017	Low	2015-2016	Poland	Hospital-based (but not ANC)	One site	440
**Rajaii**	2013	Low	2009-2010	Iran	Antenatal Care Unit	Multi-site	1659
**Ramois**	2011	Low	2006-2007	Spain	Hospital-based (but not ANC)	Multi-site	1627
**Ramsewak**	2008	Moderate	NR	Trinidad & Tobago	Antenatal Care Unit	Multi-site	232
**Ravindanath**	2012	High	NR	India	Hospital-based (but not ANC)	One site	94
**Rebouças**	2011	Moderate	NR	Brazil	Unclear/No description	NR	2229
**Reiche**	2000	Moderate	NR	Brazil	Hospital-based (but not ANC)	One site	1559
**Reis**	2006	Low	2000	Brazil	Antenatal Care Unit	One site	10408
**Ribeirro**	2008	Low	NR	Brazil	Hospital-based (but not ANC)	One site	832
**Ribes**	1996	Low	1991-1993	Spain	Hospital-based (but not ANC)	Multi-site	299
**Rodier**	1995	Low	1993	Benin	Hospital-based (but not ANC)	One site	211

**Rosso**	2008	Low	2005	Colombia	Antenatal Care Unit	Multi-site	955
**Ruffini**	2014	Low	2011-2012	Italy	Hospital-based (but not ANC)	Multi-site	10085
**Sagel**	2010	Low	2000-2005	Austria	Hospital-based (but not ANC)	One site	51754
**Saki**	2016	High	NR	Iran	Antenatal Care Unit	Multi-site	220
**Sakikawa**	2011	Low	1997-2004	Japan	Antenatal Care Unit	One site	4466
**Samara**	2015	Moderate	NR	Brazil	Hospital-based (but not ANC)	Multi-site	551
**Santoz**	1995	Low	1990-1991	Brazil	Hospital-based (but not ANC)	One site	698
**Sartori**	2011	Low	2008	Brazil	Antenatal Care Unit	One site	10316
**Sekla**	1981	Moderate	1976-1978	Canada	Antenatal Care Unit	Multi-site	55527
**Selek**	2015	Moderate	2012-2014	Turkey	Unclear/No description	NR	1737
**Selvaraj**	2017	Moderate	NR	India	Antenatal Care Unit	Multi-site	193
**Shamakhteh**	2016	Moderate	2016	Iran	Population-based	One site	150
**Sharbatkhori**	2014	Low	2012	Iran	Antenatal Care Unit	One site	555
**Shieh**	2017	Low	2015	Iran	Hospital-based (but not ANC)	One site	261
**Shirdel**	2017	Moderate	2014-2015	Iran	Unclear/No description	NR	440
**Simpore**	2006	Moderate	2004-2005	Burkina Faso	Antenatal Care Unit	One site	336
**Singh**	2016	Low	2015	India	Antenatal Care Unit	One site	260
**Singh**	2004	Low	2002-2003	India	Hospital-based (but not ANC)	One site	300
**Sitoe**	2010	Moderate	NR	Mozambique	Hospital-based (but not ANC)	One site	150
**Song**	2005	Moderate	NR	South Korea	Hospital-based (but not ANC)	One site	5925
**Spaldy**	2005	Low	1997-1998	Brazil	Hospital-based (but not ANC)	Multi-site	2126
**Sroka**	2010	Low	2005	Brazil	Antenatal Care Unit	One site	963
**Sukthana**	1999	Moderate	NR	Australia	Antenatal Care Unit	One site	300
**Sukthana**	1999	Moderate	NR	Thailand	Antenatal Care Unit	One site	300
**Tabatabaie**	2015	Low	2013	Iran	Hospital-based (but not ANC)	One site	200
**Tamer**	2009	Low	2005-2007	Turkey	Hospital-based (but not ANC)	National	1972
**Tlamcani**	2017	Low	2010-2015	Morocco	Hospital-based (but not ANC)	One site	3440
**Toklu**	2013	Moderate	2010-2011	Turkey	Hospital-based (but not ANC)	One site	1465
**Tové**	2018	Low	2016	Benin	Hospital-based (but not ANC)	Multi-site	399

**Ustaçelebi**	1986	Moderate	NR	Turkey	Hospital-based (but not ANC)	One site	301
**Uysal**	2013	Low	2003-2008	Turkey	Hospital-based (but not ANC)	One site	4651
**Valcavi**	1995	Low	1987-1991	Italy	Hospital-based (but not ANC)	One site	3602
**Van**	2017	Low	2014-2015	Thailand	Antenatal Care Unit	Multi-site	5883
**Varaghchi**	2015	Moderate	2011-2013	Iran	Antenatal Care Unit	One site	519
**Varella**	2000	Low	2000	Brazil	Antenatal Care Unit	One site	1261
**Varella**	2009	Low	1998-2005	Brazil	Antenatal Care Unit	One site	41112
**Vaz**	1990	Moderate	1988	Brazil	Hospital-based (but not ANC)	One site	481
**Vaz**	2010	Low	2003-2004	Brazil	Hospital-based (but not ANC)	One site	20389
**Vial**	1985	Low	1985	Chile	Antenatal Care Unit	One site	1304
**Vilibic-Cavlek**	2011	Moderate	2005-2009	Croatia	Antenatal Care Unit	One site	502
**Völker**	2017	Low	2011-2012	Ghana	Antenatal Care Unit	One site	180
**Wam**	2016	Low	2014	Cameroon	Antenatal Care Unit	One site	90
**Wanachiwanawi**	2001	Low	1997-1999	Thailand	Antenatal Care Unit	One site	1669
**Wang**	2016	Moderate	NR	China	Antenatal Care Unit	One site	276
**Xia**	2011	Low	NR	China	Hospital-based (but not ANC)	Multi-site	6849
**Yad**	2014	Moderate	2011	Iran	Antenatal Care Unit	One site	501
**Yang**	2016	Moderate	NR	China	Unclear/No description	Multi-site	276
**Yanping**	2014	Low	2010-2013	China	Antenatal Care Unit	One site	2740
**Yasmeen**	2017	Moderate	2014-2016	India	Hospital-based (but not ANC)	One site	251
**Zemene**	2012	Moderate	2011	Ethiopia	Population-based	Multi-site	201
**Zhang**	2013	Low	2013	China	Unclear/No description	One site	900
**Zhang**	1996	Moderate	NR	China	Unclear/No description	One site	4126
**Zhou**	2015	Moderate	NR	China	Unclear/No description	Multi-site	3014

NR: Not reported

Supplementary Table 2. Meta-analysis IgM seroprevalence of Toxoplasma gondii *in the population of pregnant women, by country*

Countries	Prevalence	95% confidence interval	95% prediction interval	N studies	Sample	Heterogeneity		Egger test, P value
						I^2, %	P value	
**Benin**	0.7	0.1-1.7	0.0-22.4	3	893	37.8	0.201	0.427
**Brazil**	1.3	0.9-1.8	0.0-4.3	26	91 582	95.6	< 0.0001	0.002
**Burkina Faso**	2.0	0.0-7.5	0.0-100	3	794	92.7	< 0.0001	0.527
**Canada**	1.0	0.1-0.2	NA	1	55 527	NA	NA	NA
**China**	1.9	1.3-2.6	0.1-5.6	16	25 974	92.2	< 0.0001	0.787
**Columbia**	1.7	0.8-2.9	0.0-29.6	3	3 380	71.7	0.029	0.986
**Cyprus**	0.6	0.5-0.7	NA	1	17 631	NA	NA	NA
**Denmark**	0.5	0.4-0.8	NA	1	5 402	NA	NA	NA
**Egypt**	4.4	1.0-10.0	0.0-100.0	3	713	87.7	0.0003	0.957
**Ethiopia**	3.3	1.8-5.3	NA	2	414	0.0	0.343	NA
**Gabon**	2.0	1.0-3.3	NA	2	1 606	64.4	0.094	NA
**India**	3.7	1.4-6.9	0.0-19.6	10	2 601	91.8	< 0.0001	0.149
**Iran**	2.5	1.5-3.8	0.0-9.7	18	6 489	87.7	< 0.0001	0.182
**Italy**	0.6	0.1-1.3	0.0-4.4	6	10 372	92.3	< 0.0001	0.651
**Morocco**	1.8	1.3-2.4	NA	1	2 456	NA	NA	NA
**New Zealand**	0.2	0.1-0.4	NA	1	5 000	NA	NA	NA
**Norway**	0.5	0.4-0.6	NA	1	35 940	NA	NA	NA
**Poland**	2.4	0.0-11.7	0.0-100	3	10 521	99.5	< 0.0001	0.374
**Saudi Arabia**	4.1	2.2-6.5	0.0-14.7	9	2 595	86.1	< 0.0001	0.326
**South Korea**	0.1	0.0-0.4	NA	2	1 138	0.0	0.610	NA
**Sudan**	2.9	0.0-9.4	NA	2	675	91.0	0.0009	NA
**Tanzania**	1.9	0.0-7.6	0.0-100	3	1 453	96.0	< 0.0001	0.663
**Thailand**	1.0	0.0-3.2	0.0-15.5	5	9 252	97.4	< 0.0001	0.192
**Turkey**	1.4	0.9-1.9	0.0-4.5	18	51 500	95.2	< 0.0001	0.832
**USA**	0.01	0.001-0.02	NA	2	96 049	0.0	0.846	NA
**Yemen**	6.0	1.6-12.8	NA	2	952	92.3	0.0003	NA

NA: not applicable

Supplementary Table 3. Predicted seroprevalence of Toxoplasma gondii in pregnant women in each county in different WHO regions

Countries	Immunoglobulins M			Immunoglobulins G		
	Prevalence, %	Lower interval	Upper Interval	Prevalence, %	Lower Interval	Upper interval
**WHO Africa**						
**Algeria**	5.7	5.3	6.0	67.5	67.0	68.1
**Angola**	3.1	2.9	3.3	61.5	61.0	62.0
**Benin**	2.3	2.1	2.4	31.3	30.9	31.8
**Botswana**	2.8	2.6	3.0	65.1	64.5	65.6
**Burkina Faso**	2.4	2.3	2.6	30.2	29.8	30.7
**Burundi**	3.4	3.2	3.5	28.2	27.9	28.6
**Cameroon**	2.3	2.1	2.5	37.8	37.3	38.3
**Cape Verde**	3.0	2.8	3.2	63.4	62.9	63.9
**Central African Republic**	3.4	3.2	3.5	28.2	27.8	28.6
**Chad**	2.4	2.2	2.5	30.4	30.0	30.9
**Comoros**	2.2	2.0	2.3	32.9	32.5	33.4
**Congo**	3.0	2.8	3.2	51.3	50.7	51.9
**Congo, Dem. Rep.**	3.2	3.0	3.3	28.5	28.1	28.8
**Cote d'Ivoire**	2.4	2.2	2.5	39.2	38.7	39.7
**Equatorial Guinea**	1.3	1.1	1.4	45.2	44.6	45.8
**Eswatini**	2.3	2.1	2.4	72.2	71.7	72.6
**Ethiopia**	2.4	2.3	2.6	30.3	29.9	30.7
**Gabon**	1.6	1.5	1.8	63.0	62.4	63.6
**Gambia**	2.5	2.4	2.7	29.9	29.5	30.3
**Ghana**	2.6	2.4	2.8	43.2	42.7	43.8
**Guinea**	2.3	2.1	2.4	31.2	30.8	31.7
**Guinea-Bissau**	2.5	2.4	2.7	29.9	29.5	30.3
**Kenya**	2.2	2.0	2.4	35.4	34.9	35.8
**Lesotho**	2.2	2.0	2.3	33.7	33.2	34.1
**Liberia**	2.8	2.6	3.0	29.1	28.7	29.5
**Madagascar**	2.6	2.5	2.8	29.6	29.2	30.0
**Malawi**	2.9	2.7	3.0	29.0	28.6	29.4
**Mali**	2.3	2.1	2.4	31.2	30.7	31.6
**Mauritania**	2.4	2.2	2.5	39.3	38.7	39.8
**Mauritius**	0.7	0.6	0.8	50.2	49.6	50.8
**Mozambique**	2.8	2.7	3.0	29.0	28.6	29.4
**Namibia**	2.5	2.4	2.7	74.3	73.9	74.7
**Niger**	3.1	2.9	3.2	28.7	28.3	29.1
**Nigeria**	3.1	2.9	3.3	55.1	54.6	55.7
**Rwanda**	2.3	2.2	2.5	30.7	30.3	31.1
**Sao Tome and Principe**	2.2	2.0	2.4	35.7	35.2	36.2
**Senegal**	2.2	2.1	2.4	36.2	35.7	36.8
**Seychelles**	1.1	1.0	1.2	51.4	50.8	52.0
**Sierra Leone**	2.6	2.5	2.8	29.5	29.1	29.9
**South Africa**	6.2	5.8	6.5	70.1	69.6	70.6
**Tanzania**	2.2	2.0	2.3	33.8	33.3	34.2
**Togo**	2.5	2.4	2.7	29.8	29.4	30.2
**Uganda**	2.4	2.3	2.6	30.2	29.8	30.7
**Zambia**	2.4	2.2	2.6	39.8	39.3	40.3
**Zimbabwe**	2.2	2.1	2.4	31.8	31.4	32.3
**WHO Americas**						
**Antigua and Barbuda**	0.3	0.3	0.4	29.4	29.0	29.7

Countries	Immunoglobulins M			Immunoglobulins G		
	Prevalence, %	Lower interval	Upper Interval	Prevalence, %	Lower Interval	Upper interval
**Argentina**	0.2	0.2	0.3	36.7	36.3	37.1
**Aruba**	1.1	1.0	1.3	45.7	45.2	46.1
**Bahamas, The**	0.3	0.3	0.3	36.1	35.7	36.5
**Barbados**	0.5	0.4	0.5	43.4	42.9	43.8
**Belize**	0.8	0.8	0.9	54.4	54.1	54.8
**Bolivia**	1.0	0.9	1.1	49.5	49.1	49.8
**Brazil**	2.0	1.9	2.1	49.1	48.7	49.5
**Canada**	0.02	0.01	0.03	0.3	0.2	0.3
**Cayman Islands**	0.0	0.0	0.1	20.5	19.7	21.4
**Chile**	0.5	0.5	0.6	27.4	27.0	27.8
**Colombia**	2.4	2.3	2.5	50.7	50.3	51.1
**Costa Rica**	1.0	0.9	1.1	46.6	46.1	47.0
**Curacao**	0.3	0.3	0.3	36.6	36.2	37.0
**Dominica**	0.9	0.8	0.9	57.6	57.3	58.0
**Dominican Republic**	1.6	1.5	1.7	48.3	47.9	48.7
**Ecuador**	1.3	1.2	1.4	56.3	56.0	56.7
**El Salvador**	0.9	0.8	1.0	52.1	51.7	52.4
**Grenada**	2.2	2.0	2.3	49.7	49.3	50.0
**Guatemala**	0.9	0.8	1.0	52.8	52.5	53.2
**Guyana**	0.9	0.8	1.0	52.9	52.5	53.2
**Haiti**	0.9	0.8	1.0	16.9	16.6	17.1
**Honduras**	1.0	1.0	1.1	29.7	29.4	30.0
**Jamaica**	0.8	0.7	0.9	56.2	55.9	56.6
**Marshall Islands**	0.9	0.8	1.0	24.9	24.6	25.1
**Mexico**	0.4	0.3	0.4	42.0	41.6	42.5
**Nicaragua**	1.1	1.1	1.2	36.3	36.0	36.7
**Panama**	0.5	0.4	0.5	27.8	27.5	28.2
**Paraguay**	2.1	2.0	2.2	53.3	52.9	53.7
**Peru**	2.3	2.2	2.4	52.6	52.2	52.9
**Puerto Rico**	1.1	0.9	1.2	44.7	44.3	45.1
**St. Kitts and Nevis**	0.5	0.5	0.6	31.5	31.1	31.9
**St. Lucia**	2.4	2.3	2.6	51.6	51.2	52.0
**St. Vincent and the Grenadines**	1.4	1.3	1.5	56.0	55.7	56.4
**Suriname**	2.2	2.0	2.3	49.6	49.2	50.0
**Tonga**	1.2	1.1	1.2	37.3	37.0	37.6
**Trinidad & Tobago**	0.2	0.2	0.3	38.4	38.1	38.8
**Uruguay**	0.3	0.2	0.3	31.4	31.0	31.8
**USA**	0.03	0.01	0.07	0.03	0.02	0.05
**Vanuatu**	0.8	0.7	0.9	20.2	19.9	20.4
**Venezuela**	0.5	0.5	0.6	43.7	43.3	44.1
**WHO Eastern Mediterranean**						
**Afghanistan**	4.1	3.9	4.3	24.2	23.9	24.5
**Bahrain**	0.8	0.5	1.2	71.5	70.6	72.5
**Djibouti**	3.8	3.6	4.0	27.1	26.8	27.5
**Egypt**	6.2	5.9	6.5	66.6	66.2	67.0
**Iran**	1.1	1.1	1.2	47.1	46.7	47.5
**Iraq**	5.1	4.8	5.4	57.7	57.3	58.1
**Jordan**	3.8	3.6	4.0	66.7	66.4	67.1
**Kuwait**	0.03	0.01	0.07	1.7	1.4	2.0
**Lebanon**	10.8	10.5	11.2	61.4	60.9	61.8

Countries	Immunoglobulins M			Immunoglobulins G		
	Prevalence, %	Lower interval	Upper Interval	Prevalence, %	Lower Interval	Upper interval
**Libya**	1.5	1.4	1.6	51.0	50.6	51.5
**Morocco**	4.2	4.0	4.4	63.7	63.3	64.0
**Oman**	5.1	4.3	6.1	61.8	61.2	62.4
**Pakistan**	5.3	5.1	5.5	44.1	43.7	44.5
**Palestine**	4.8	4.6	5.0	38.7	38.3	39.0
**Qatar**	0.03	0.01	0.07	0.00001	0.00	0.00001
**Saudi Arabia**	0.0	0.0	0.1	52.2	51.4	52.9
**Sudan**	4.8	4.7	5.1	38.8	38.4	39.2
**Tunisia**	6.9	6.6	7.2	66.0	65.7	66.4
**United Arab Emirates**	0.03	0.01	0.07	1.1	0.9	1.3
**Yemen**	3.8	3.6	4.0	25.8	25.5	26.2
**WHO Europe**						
**Albania**	2.1	2.0	2.2	36.1	35.8	36.5
**Armenia**	0.8	0.8	0.9	39.7	39.4	40.1
**Austria**	0.1	0.1	0.2	36.4	35.5	37.4
**Azerbaijan**	0.8	0.8	0.9	29.4	29.1	29.8
**Belarus**	0.4	0.4	0.5	26.7	26.3	27.0
**Belgium**	0.5	0.3	0.8	39.5	38.4	40.5
**Bosnia and Herzegovina**	2.0	1.9	2.1	36.3	36.0	36.6
**Bulgaria**	0.6	0.6	0.7	19.7	19.4	20.0
**Croatia**	1.4	1.2	1.5	13.2	13.0	13.5
**Cyprus**	1.0	0.9	1.0	22.5	22.2	22.8
**Czech Republic**	1.0	0.9	1.1	22.6	22.2	22.8
**Denmark**	0.1	0.0	0.1	32.1	31.3	32.8
**Estonia**	0.6	0.5	0.6	20.9	20.7	21.2
**Finland**	1.3	0.9	1.7	36.3	35.4	37.1
**France**	2.4	1.9	2.9	31.4	30.8	31.9
**Georgia**	1.0	0.9	1.1	39.9	39.5	40.2
**Germany**	0.2	0.1	0.3	37.0	36.0	38.1
**Greece**	1.6	1.5	1.8	13.7	13.5	14.0
**Hungary**	1.0	0.9	1.1	16.9	16.7	17.2
**Iceland**	0.0	0.0	0.1	37.3	36.4	38.2
**Ireland**	0.03	0.01	0.07	0.3	0.2	0.4
**Israel**	0.5	0.5	0.6	26.4	26.0	26.7
**Italy**	2.5	2.2	2.8	24.4	24.1	24.7
**Kazakhstan**	0.7	0.6	0.8	15.8	15.5	16.1
**Kosovo**	1.0	0.9	1.1	39.9	39.5	40.2
**Kyrgyz Republic**	0.8	0.8	0.9	12.3	12.1	12.5
**Latvia**	1.6	1.4	1.7	14.3	14.0	14.5
**Lithuania**	0.6	0.5	0.6	21.0	20.7	21.3
**Luxembourg**	0.03	0.01	0.07	0.0003	0.00	0.002
**Macedonia, FYR**	2.4	2.3	2.5	33.6	33.3	33.9
**Malta**	2.9	2.5	3.4	26.4	26.1	26.8
**Moldova**	1.1	1.1	1.2	21.0	20.7	21.3
**Montenegro**	0.6	0.5	0.6	28.2	27.8	28.6
**Netherlands**	0.0	0.0	0.1	24.4	23.8	24.9
**Norway**	0.03	0.01	0.07	0.6	0.5	0.8
**Poland**	0.8	0.8	0.9	17.9	17.7	18.2
**Portugal**	0.7	0.7	0.8	19.0	18.7	19.2
**Romania**	1.6	1.4	1.7	13.2	12.9	13.4
**Russian**	0.7	0.6	0.7	16.4	16.2	16.7

Countries	Immunoglobulins M			Immunoglobulins G		
	Prevalence, %	Lower interval	Upper Interval	Prevalence, %	Lower Interval	Upper interval
**San Marino**	0.0	0.0	0.1	3.7	3.3	4.1
**Serbia**	2.0	1.9	2.1	32.2	31.9	32.5
**Slovak Republic**	0.6	0.5	0.6	21.5	21.2	21.8
**Slovenia**	0.7	0.6	0.8	22.2	21.9	22.4
**Spain**	1.9	1.7	2.2	23.4	23.1	23.7
**Sweden**	0.1	0.0	0.1	31.0	30.2	31.8
**Switzerland**	0.0	0.0	0.1	4.0	3.6	4.4
**Tajikistan**	0.8	0.7	0.9	11.0	10.8	11.2
**Turkey**	0.6	0.6	0.7	16.9	16.6	17.2
**Turkmenistan**	0.6	0.6	0.7	28.4	28.0	28.8
**UK**	1.8	1.3	2.2	34.3	33.6	35.0
**Ukraine**	0.8	0.8	0.9	37.5	37.2	37.9
**Uzbekistan**	1.1	1.0	1.2	28.3	28.0	28.6
**WHO South East Asia**						
**Bangladesh**	1.0	0.9	1.1	26.1	25.7	26.5
**Bhutan**	1.0	0.9	1.0	61.1	60.6	61.7
**India**	1.3	1.2	1.4	50.4	49.9	50.9
**Indonesia**	2.0	1.8	2.2	59.2	58.7	59.7
**Maldives**	1.4	1.3	1.6	51.6	51.1	52.2
**Myanmar**	1.4	1.3	1.5	43.2	42.7	43.7
**Nepal**	0.9	0.9	1.0	21.4	21.1	21.8
**Sri Lanka**	2.4	2.2	2.5	58.1	57.6	58.6
**Thailand**	0.8	0.7	0.9	49.3	48.7	49.8
**Timor-Leste**	1.2	1.2	1.3	51.5	51.0	52.1
**WHO Western Pacific**						
**Australia**	0.1	0.1	0.2	16.6	16.0	17.3
**Brunei Darussalam**	0.03	0.01	0.07	0.03	0.02	0.04
**Cambodia**	1.2	1.1	1.3	3.8	3.7	4.0
**China**	1.6	1.5	1.7	10.3	10.1	10.5
**Fiji**	1.1	1.0	1.2	14.8	14.5	15.0
**Hong Kong SAR, China**	0.03	0.01	0.07	1.5	1.4	1.7
**Japan**	1.2	1.0	1.5	13.3	12.9	13.7
**Kiribati**	1.2	1.1	1.2	2.7	2.6	2.8
**Lao PDR**	1.5	1.4	1.6	9.8	9.6	10.0
**Malaysia**	0.5	0.5	0.6	6.2	6.0	6.3
**Micronesia, Fed. Sts.**	1.1	1.1	1.2	3.5	3.4	3.7
**Mongolia**	2.8	2.7	2.9	13.0	12.8	13.3
**Nauru**	3.4	3.2	3.5	12.0	11.7	12.2
**New Zealand**	1.6	1.4	1.9	10.0	9.7	10.2
**Palau**	3.2	3.0	3.3	11.5	11.3	11.7
**Papua New Guinea**	1.2	1.2	1.3	4.1	3.9	4.2
**Philippines**	1.2	1.1	1.3	13.1	12.8	13.3
**Samoa**	1.6	1.5	1.6	8.8	8.6	9.0
**Singapore**	0.03	0.01	0.07	0.001	0.000	0.003
**Solomon Islands**	1.1	1.0	1.2	2.8	2.7	2.9
**South Korea**	1.6	1.4	1.8	9.8	9.6	10.1
**Tuvalu**	1.2	1.1	1.3	3.8	3.6	3.9
**Vietnam**	1.5	1.4	1.6	9.2	9.0	9.4

Supplementary Table 4. Meta-analysis IgG seroprevalence of Toxoplasma gondii *in the population of pregnant women, by country*

Countries	Prevalence	95% confidence interval	95% prediction interval	N studies	Sample	Heterogeneity		Egger test, P value
						I^2, %	P value	
**Australia**	26.6	20.4-33.3	NA	2	608	70.6	0.065	NA
**Benin**	39.6	27.5-52.3	0.0-100	3	893	93.2	< 0.0001	0.559
**Brazil**	53.8	39.3-68.0	0.0-100	33	118 324	100	< 0.0001	0.002
**Burkina Faso**	21.6	12.4-32.5	0.0-77.1	4	1 110	94.1	< 0.0001	0.851
**Cameroon**	45.6	11.3-82.5	0.0-100	4	502	98.7	< 0.0001	0.449
**Canada**	0.2	0.2-0.3	NA	1	55 527	NA	NA	NA
**Chile**	35.2	29.7-40.9	NA	2	1 443	52.6	< 0.0001	NA
**China**	8.7	5.8-12.2	0.1-27.7	16	30 322	98.9	< 0.0001	0.762
**Columbia**	37.8	26.9-49.4	0.9-88.6	4	3 681	97.3	< 0.0001	0.490
**Croatia**	30.1	27.1-33.1	NA	2	900	0.0	< 0.0001	NA
**Denmark**	27.4	26.2-28.3	NA	1	5 402	NA	NA	NA
**Egypt**	22.9	0.0-71.8	0.0-100	3	713	99.5	< 0.0001	0.128
**Ethiopia**	64.2	34.3-89.1	0.0-100	6	1 816	99.4	< 0.0001	0.232
**Finland**	20.3	19.7-20.9	NA	1	16 733	NA	NA	NA
**Gabon**	56.7	54.4-59.0	NA	2	1 812	0.0	0.599	NA
**India**	26.6	18.6-35.5	2.9-62.1	10	3 622	96.8	< 0.0001	0.117
**Iran**	41.2	33.7-48.8	8.8-78.8	23	9 851	98.3	< 0.0001	0.407
**Italy**	27.9	21.4-34.9	7.3-55.3	8	31 324	99.4	< 0.0001	0.678
**Malaysia**	35.9	30.6-41.4	NA	2	481	35.9	0.212	NA
**Mexico**	7.2	5.3-9.4	NA	2	782	16.8	0.273	NA
**Morocco**	44.3	35.7-53.1	0.0-100	3	6 024	97.1	< 0.0001	0.939
**New Zealand**	21.3	20.1-22.4	NA	1	5 000	NA	NA	NA
**Nigeria**	45.1	34.6-55.7	NA	2	539	82.4	< 0.0001	NA
**Norway**	10.9	10.6-11.2	NA	1	35 940	NA	NA	NA
**Pakistan**	31.3	8.6-60.3	NA	2	1 057	98.8	< 0.0001	NA
**Poland**	32.7	23.2-43.0	3.4-73.5	5	14 457	99.2	< 0.0001	0.419
**Portugal**	18.0	6.3-33.9	NA	2	3 317	95.2	< 0.0001	NA
**Saudi Arabia**	31.5	25.6-37.9	11.6-55.9	10	4 771	94.3	< 0.0001	0.167
**South Korea**	2.1	0.6-4.3	0.0-65.2	3	7 063	91.9	< 0.0001	0.010
**Spain**	27.4	19.1-36.6	2.6-64.9	5	11 350	98.5	< 0.0001	0.638
**Sudan**	34.9	31.4-38.6	NA	2	675	0.0	0.439	NA
**Sweden**	26.3	6.1-54.0	NA	2	7 445	99.8	< 0.0001	NA
**Switzerland**	46.1	45.1-47.1	NA	1	9 059	NA	NA	NA
**Tanzania**	35.3	26.5-44.6	NA	2	604	82.0	0.018	NA
**Thailand**	21.2	15.0-28.2	2.5-51.1	5	4 550	96.5	< 0.0001	0.684
**Turkey**	35.8	30.6-41.3	11.8-64.6	24	67 628	99.5	< 0.0001	0.972
**UK**	11.0	3.1-23.0	0.0-81.7	4	24 584	99.8	< 0.0001	0.416
**Venezuela**	37.4	34.0-40.8	NA	2	778	0.0	0.338	NA
**Yemen**	44.6	41.5-47.8	NA	2	952	0.0	0.441	NA

NA: not applicable

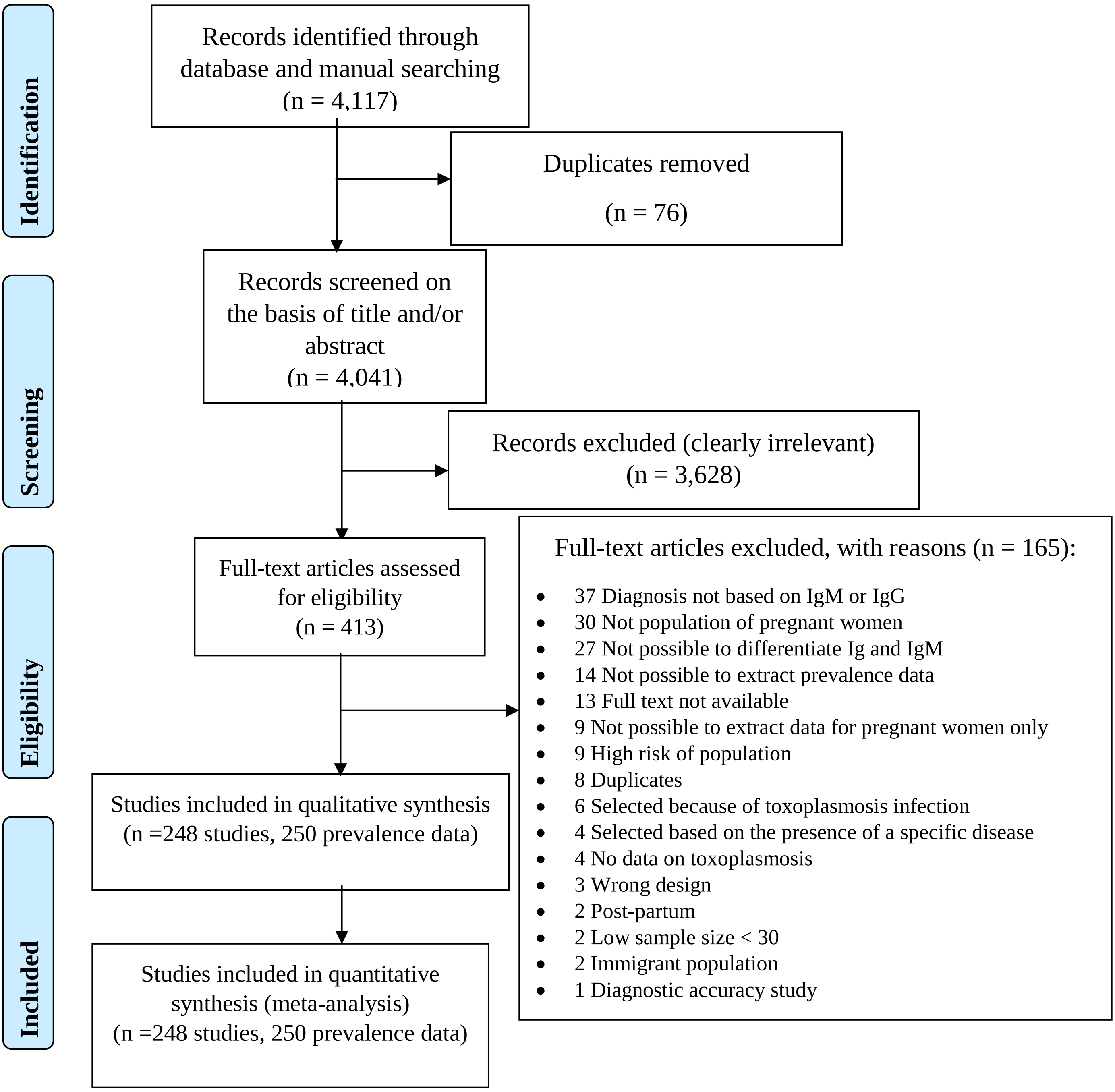

Supplementary Figure 1. Process of identification and selection of studies for inclusion in the review (PRISMA flow diagram)

Supplementary Figure 2. Meta-analysis of IgM seroprevalence of Toxoplasma gondii among pregnant women in WHO Africa

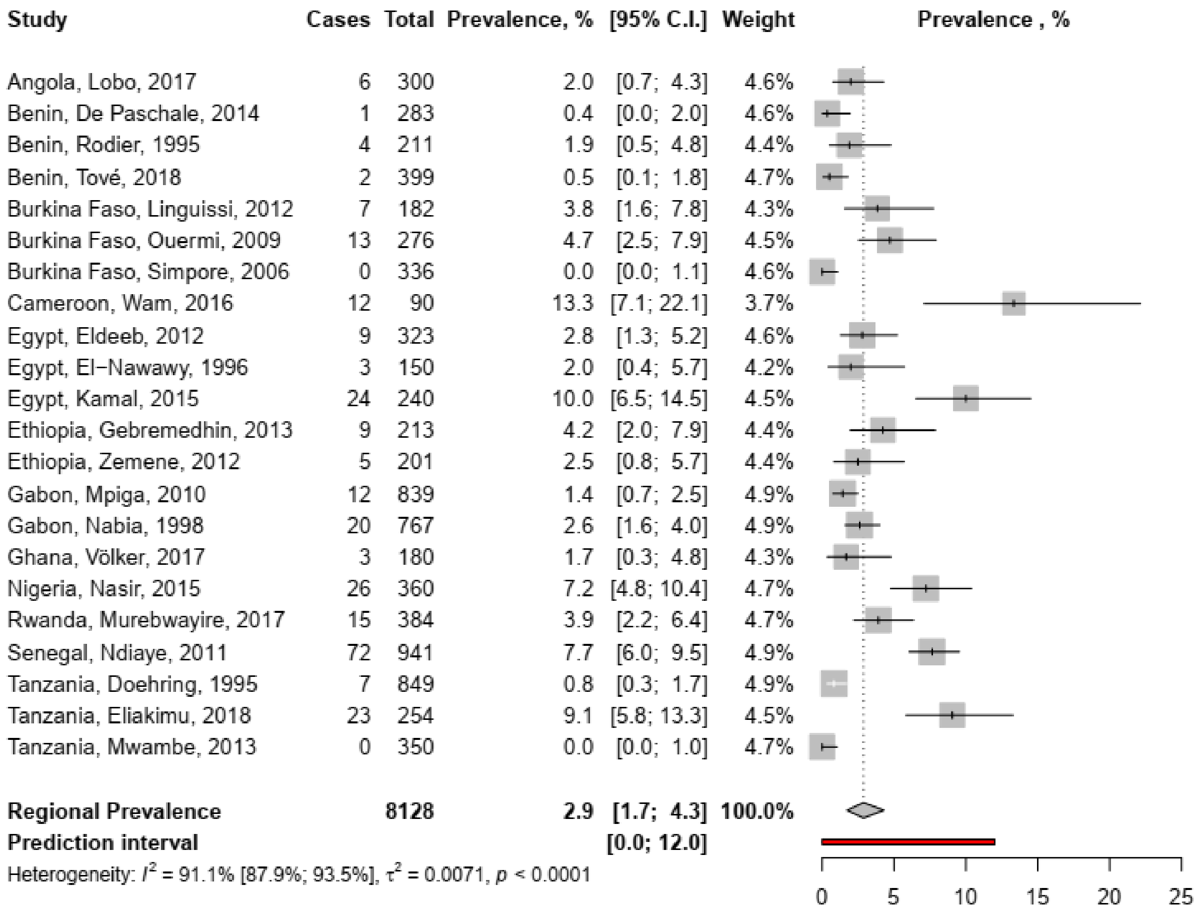

Supplementary Figure 3. Meta-analysis of IgM seroprevalence of Toxoplasma gondii among pregnant women in WHO Americas

Study	Cases	Total	Prevalence, %	[95% C.I.]	Weight
Brazil, Avelar, 2017	0	712	0.0	[0.0; 0.5]	2.8%
Brazil, Boa−Sorte, 2014	30	692	4.3	[2.9; 6.1]	2.8%
Brazil, Cinara, 2011	2	87	2.3	[0.3; 8.1]	1.2%
Brazil, Costa, 2018	27	726	3.7	[2.5; 5.4]	2.8%
Brazil, Da Rocha, 2015	0	338	0.0	[0.0; 1.1]	2.3%
Brazil, Dasilva, 2014	28	487	5.7	[3.9; 8.2]	2.6%
Brazil, De Moura, 2013	17	400	4.2	[2.5; 6.7]	2.4%
Brazil, De Quadros, 2015	2	148	1.4	[0.2; 4.8]	1.6%
Brazil, Dias, 2011	7	607	1.2	[0.5; 2.4]	2.7%
Brazil, Ferezin, 2013	16	1534	1.0	[0.6; 1.7]	3.2%
Brazil, Figueiró−Filho, 2007	137	32512	0.4	[0.4; 0.5]	3.5%
Brazil, Fonseca, 2012	77	2136	3.6	[2.9; 4.5]	3.3%
Brazil, Gonçalves, 2010	19	574	3.3	[2.0; 5.1]	2.7%
Brazil, Guerra, 2014	11	2673	0.4	[0.2; 0.7]	3.3%
Brazil, Inagaki, 2009	40	9550	0.4	[0.3; 0.6]	3.5%
Brazil, Isabelle Ribeiro, 2009	1	190	0.5	[0.0; 2.9]	1.8%
Brazil, Lopez, 2009	4	492	0.8	[0.2; 2.1]	2.6%
Brazil, Mendez, 2009	1	268	0.4	[0.0; 2.1]	2.1%
Brazil, Reiche, 2000	9	1559	0.6	[0.3; 1.1]	3.2%
Brazil, Reis, 2006	19	10408	0.2	[0.1; 0.3]	3.5%
Brazil, Ribeirro, 2008	17	832	2.0	[1.2; 3.3]	2.9%
Brazil, Santoz, 1995	20	698	2.9	[1.8; 4.4]	2.8%
Brazil, Spaldy, 2005	77	2126	3.6	[2.9; 4.5]	3.3%
Brazil, Sroka, 2010	5	963	0.5	[0.2; 1.2]	3.0%
Brazil, Vaz, 1990	6	481	1.2	[0.5; 2.7]	2.6%
Brazil, Vaz, 2010	115	20389	0.6	[0.5; 0.7]	3.5%
Canada, Sekla, 1981	70	55527	0.1	[0.1; 0.2]	3.5%
Chile, Contreras, 1995	0	139	0.0	[0.0; 2.6]	1.6%
Colombia, Angel−Nueller, 2011	34	2258	1.5	[1.0; 2.1]	3.3%
Colombia, Cardenas, 2011	1	167	0.6	[0.0; 3.3]	1.7%
Colombia, Rosso, 2008	27	955	2.8	[1.9; 4.1]	3.0%
Grenada, Asthana, 2006	0	534	0.0	[0.0; 0.7]	2.7%
Mexico, Alvarado−Esquivel, 2009	10	439	2.3	[1.1; 4.1]	2.5%
Trinidad & Tobago, Ramsewak, 2008	8	232	3.4	[1.5; 6.7]	2.0%
USA, Beach, 1979	129	95929	0.1	[0.1; 0.2]	3.5%
USA, Hershey, 1987	0	120	0.0	[0.0; 3.0]	1.4%
Venezuela, De Noya, 2010	8	678	1.2	[0.5; 2.3]	2.8%
**Regional Prevalence**		**248560**	**1.1**	**[0.8; 1.4]**	**100.0%**
**Prediction interval**				**[0.0; 3.4]**	

Heterogeneity: I^2 = 96.8% [96.2%; 97.3%], τ^2 = 0.0014, p < 0.0001

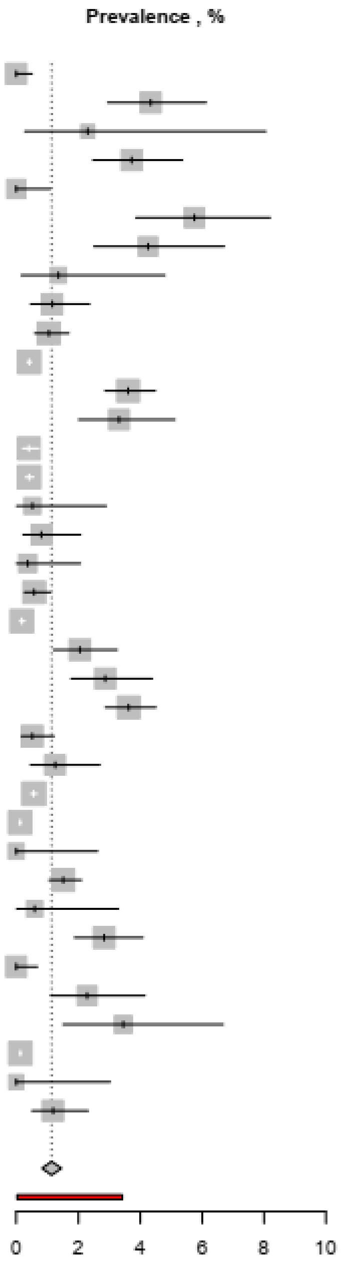

Supplementary Figure 4. Meta-analysis of IgM seroprevalence of Toxoplasma gondii among pregnant women in WHO Eastern Mediterranean

Study	Cases	Total	Prevalence, %	[95% C.I.]	Weight
Iran, Abolghasem, 2011	2	225	0.9	[0.1; 3.2]	2.7%
Iran, Ahmadpour, 2015	15	264	5.7	[3.2; 9.2]	2.8%
Iran, Akhlaghi, 2016	9	468	1.9	[0.9; 3.6]	2.9%
Iran, Esboei, 2016	43	786	5.5	[4.0; 7.3]	3.0%
Iran, Garedaghi, 2017	5	200	2.5	[0.8; 5.7]	2.7%
Iran, Hajsoleimani, 2012	7	500	1.4	[0.6; 2.9]	2.9%
Iran, Hoseini, 2014	7	289	2.4	[1.0; 4.9]	2.8%
Iran, Josheghani, 2015	3	80	3.8	[0.8; 10.6]	2.3%
Iran, Mohammad, 2011	65	721	9.0	[7.0; 11.3]	3.0%
Iran, Mousavi, 2018	0	110	0.0	[0.0; 3.3]	2.5%
Iran, Saki, 2016	3	220	1.4	[0.3; 3.9]	2.7%
Iran, Shamakhteh, 2016	7	150	4.7	[1.9; 9.4]	2.6%
Iran, Sharbatkhori, 2014	19	555	3.4	[2.1; 5.3]	2.9%
Iran, Shieh, 2017	1	261	0.4	[0.0; 2.1]	2.8%
Iran, Shirdel, 2017	25	440	5.7	[3.7; 8.3]	2.9%
Iran, Tabatabaie, 2015	4	200	2.0	[0.5; 5.0]	2.7%
Iran, Varaghchi, 2015	2	519	0.4	[0.0; 1.4]	2.9%
Iran, Yad, 2014	7	501	1.4	[0.6; 2.9]	2.9%
Iraq, Ali, 2017	13	161	8.1	[4.4; 13.4]	2.6%
Kuwait, Iqbal, 2007	31	224	13.8	[9.6; 19.1]	2.7%
Morocco, Mansouri, 2007	45	2456	1.8	[1.3; 2.4]	3.0%
Pakistan, Bari, 1990	90	324	27.8	[23.0; 33.0]	2.8%
Palestine, Nigem, 2009	36	204	17.6	[12.7; 23.6]	2.7%
Saudi Arabia, Almushait, 2014	30	487	6.2	[4.2; 8.7]	2.9%
Saudi Arabia, Aqueely, 2014	12	195	6.2	[3.2; 10.5]	2.7%
Saudi Arabia, Bin, 2012	9	137	6.6	[3.0; 12.1]	2.6%
Saudi Arabia, Elsafi, 2015	12	400	3.0	[1.6; 5.2]	2.9%
Saudi Arabia, Imam, 2016	0	150	0.0	[0.0; 2.4]	2.6%
Saudi Arabia, Ismail, 2014	3	96	3.1	[0.6; 8.9]	2.4%
Saudi Arabia, Jawahir, 2016	16	250	6.4	[3.7; 10.2]	2.8%
Saudi Arabia, Mohamed, 2016	4	326	1.2	[0.3; 3.1]	2.8%
Saudi Arabia, Mohammad, 2013	49	554	8.8	[6.6; 11.5]	2.9%
Sudan, Elnahas, 2003	5	487	1.0	[0.3; 2.4]	2.9%
Sudan, Maha, 2012	11	188	5.9	[3.0; 10.2]	2.7%
Yemen, Al−Eryani, 2016	54	593	9.1	[6.9; 11.7]	2.9%
Yemen, Mahdy, 2017	12	359	3.3	[1.7; 5.8]	2.9%
**Regional Prevalence**		**14080**	**4.1**	**[2.8; 5.5]**	**100.0%**
**Prediction interval**				**[0.0; 15.7]**	

Heterogeneity: $I^2 = 93.6\%$ [92.1%; 94.9%], $\tau^2 = 0.0096$, $p < 0.0001$

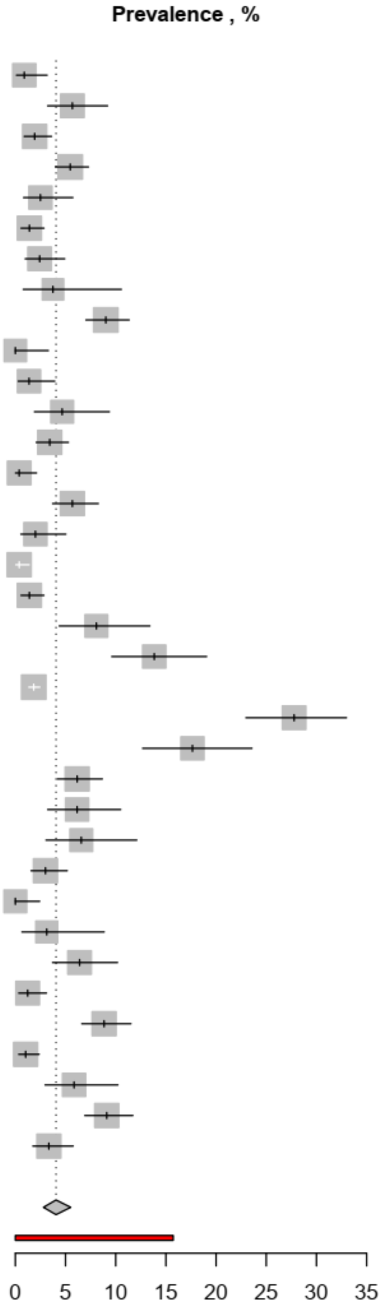

Supplementary Figure 5. Meta-analysis of IgM seroprevalence of Toxoplasma gondii among pregnant women in WHO Europe

Study	Cases	Total	Prevalence, %	[95% C.I.]	Weight
Albania, Abazaj, 2016	9	152	5.9	[2.7; 10.9]	2.1%
Croatia, Vilibic−Cavlek, 2011	1	502	0.2	[0.0; 1.1]	2.5%
Cyprus, Marios, 2016	107	17631	0.6	[0.5; 0.7]	2.8%
Denmark, Lebech, 1993	29	5402	0.5	[0.4; 0.8]	2.8%
Germany, Freise, 1993	29	512	5.7	[3.8; 8.0]	2.5%
Iceland, Iddawela, 2017	2	536	0.4	[0.0; 1.3]	2.5%
Israel, Franklin, 1993	3	213	1.4	[0.3; 4.1]	2.2%
Italy, Dentico, 2011	4	334	1.2	[0.3; 3.0]	2.4%
Italy, Depaschale, 2008	42	3426	1.2	[0.9; 1.7]	2.7%
Italy, Leone, 1996	12	1668	0.7	[0.4; 1.3]	2.7%
Italy, Maggi, 2008	3	496	0.6	[0.1; 1.8]	2.5%
Italy, Pucio, 2014	3	846	0.4	[0.1; 1.0]	2.6%
Italy, Valcavi, 1995	1	3602	0.0	[0.0; 0.2]	2.7%
Norway, Jenum, 1998	181	35940	0.5	[0.4; 0.6]	2.8%
Poland, Marcinek, 2008	9	1800	0.5	[0.2; 0.9]	2.7%
Poland, Nowakowska, 2013	803	8281	9.7	[9.1; 10.4]	2.8%
Poland, Radon−Pokracka, 2017	3	440	0.7	[0.1; 2.0]	2.5%
Portugal, Lobo, 2017	0	155	0.0	[0.0; 2.4]	2.1%
Russia, Boronina, 2018	6	65	9.2	[3.5; 19.0]	1.6%
Spain, Jaqueti, 1991	15	1221	1.2	[0.7; 2.0]	2.7%
Turkey, Aynioglu, 2015	23	910	2.5	[1.6; 3.8]	2.6%
Turkey, Aysun, 2011	15	1102	1.4	[0.8; 2.2]	2.7%
Turkey, Beytur, 2010	2	205	1.0	[0.1; 3.5]	2.2%
Turkey, Çekin, 2011	181	7520	2.4	[2.1; 2.8]	2.8%
Turkey, Dogan, 2014	13	1709	0.8	[0.4; 1.3]	2.7%
Turkey, Durdu, 2017	0	102	0.0	[0.0; 3.6]	1.9%
Turkey, Fusun, 2011	13	1646	0.8	[0.4; 1.3]	2.7%
Turkey, Gencer, 2014	5	196	2.6	[0.8; 5.9]	2.2%
Turkey, Gundem, 2014	1	419	0.2	[0.0; 1.3]	2.5%
Turkey, Harma, 2004	35	1149	3.0	[2.1; 4.2]	2.7%
Turkey, Mumcuoglu, 2014	11	6140	0.2	[0.1; 0.3]	2.8%
Turkey, Mumtaz, 2017	143	7513	1.9	[1.6; 2.2]	2.8%
Turkey, Ocak, 2007	9	1652	0.5	[0.2; 1.0]	2.7%
Turkey, Okyay, 2013	120	3340	3.6	[3.0; 4.3]	2.7%
Turkey, Parlak, 2015	108	9809	1.1	[0.9; 1.3]	2.8%
Turkey, Tamer, 2009	39	1972	2.0	[1.4; 2.7]	2.7%
Turkey, Toklu, 2013	44	1465	3.0	[2.2; 4.0]	2.7%
Turkey, Uysal, 2013	48	4651	1.0	[0.8; 1.4]	2.8%
UK, Allain, 1998	7	13328	0.1	[0.0; 0.1]	2.8%
**Regional Prevalence**		**148050**	**1.2**	**[0.8; 1.8]**	**100.0%**
**Prediction interval**				**[0.0; 6.5]**	

Heterogeneity: I^2 = 98.6% [98.4%; 98.7%], τ^2 = 0.0049, p = 0

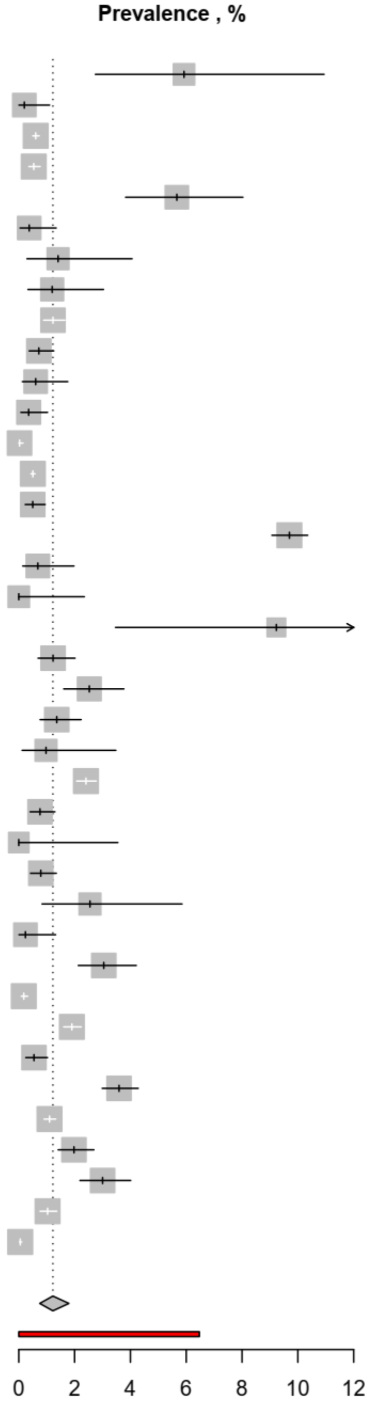

Supplementary Figure 6. Meta-analysis of IgM seroprevalence of Toxoplasma gondii among pregnant women in WHO South-East Asia

Study	Cases	Total	Prevalence, %	[95% C.I.]	Weight
Bangladesh, Ashrafunnessa, 1998	1	286	0.3	[0.0; 1.9]	5.9%
India, Amar, 2015	2	103	1.9	[0.2; 6.8]	5.2%
India, Borkakoty, 2007	14	180	7.8	[4.3; 12.7]	5.7%
India, Iqbal, 2010	9	800	1.1	[0.5; 2.1]	6.3%
India, Kaur, 1999	14	120	11.7	[6.5; 18.8]	5.3%
India, Khurana, 2010	15	300	5.0	[2.8; 8.1]	6.0%
India, Ravindanath, 2012	12	94	12.8	[6.8; 21.2]	5.1%
India, Selvaraj, 2017	0	193	0.0	[0.0; 1.9]	5.7%
India, Singh, 2016	23	260	8.8	[5.7; 13.0]	5.9%
India, Singh, 2004	7	300	2.3	[0.9; 4.7]	6.0%
India, Yasmeen, 2017	0	251	0.0	[0.0; 1.5]	5.9%
Sri Lanka, Chandrenasa, 2016	0	293	0.0	[0.0; 1.3]	5.9%
Thailand, Andiappan, 2014	19	760	2.5	[1.5; 3.9]	6.3%
Thailand, Nissapatom, 2011	39	640	6.1	[4.4; 8.2]	6.2%
Thailand, Sukthana, 1999	0	300	0.0	[0.0; 1.2]	6.0%
Thailand, Van, 2017	3	5883	0.1	[0.0; 0.1]	6.4%
Thailand, Wanachiwanawi, 2001	5	1669	0.3	[0.1; 0.7]	6.4%
**Regional Prevalence**		**12432**	**2.1**	**[0.9; 3.8]**	**100.0%**
**Prediction interval**				**[0.0; 12.8]**	

Heterogeneity: $I^2 = 95.7\%$ [94.3%; 96.8%], $\tau^2 = 0.0096$, $p < 0.0001$

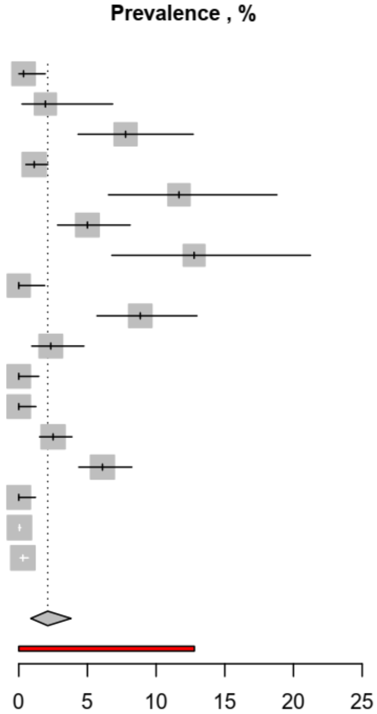

Supplementary Figure 7. Meta-analysis of IgM seroprevalence of Toxoplasma gondii among pregnant women in Western Pacific

Study	Cases	Total	Prevalence, %	[95% C.I.]	Weight
Australia, Sukthana, 1999	19	300	6.3	[3.9; 9.7]	4.2%
China, Gao, 2017	1	404	0.2	[0.0; 1.4]	4.4%
China, Jiang, 2018	4	313	1.3	[0.3; 3.2]	4.2%
China, Lin, 2008	11	426	2.6	[1.3; 4.6]	4.5%
China, Zhang, 2013	18	900	2.0	[1.2; 3.1]	4.9%
China, Cong, 2015	28	965	2.9	[1.9; 4.2]	4.9%
China, Yanping, 2014	44	2740	1.6	[1.2; 2.1]	5.1%
China, Xia, 2011	87	6849	1.3	[1.0; 1.6]	5.2%
China, Hua, 2013	0	1081	0.0	[0.0; 0.3]	4.9%
China, Hung, 2015	0	104	0.0	[0.0; 3.5]	3.0%
China, Chou, 2011	6	200	3.0	[1.1; 6.4]	3.8%
China, Yang, 2016	15	276	5.4	[3.1; 8.8]	4.1%
China, Wang, 2016	15	276	5.4	[3.1; 8.8]	4.1%
China, Zhou, 2015	35	3014	1.2	[0.8; 1.6]	5.1%
China, Qin, 2011	36	1307	2.8	[1.9; 3.8]	5.0%
China, Chen, 2014	87	2993	2.9	[2.3; 3.6]	5.1%
China, Zhang, 1996	139	4126	3.4	[2.8; 4.0]	5.2%
Malaysia, Nissapatorn, 2003	8	200	4.0	[1.7; 7.7]	3.8%
New Zealand, Morris, 2004	12	5000	0.2	[0.1; 0.4]	5.2%
South Korea, Han, 2008	0	351	0.0	[0.0; 1.0]	4.3%
South Korea, Gye−Cheol, 2011	1	787	0.1	[0.0; 0.7]	4.8%
Vietnam, Buchy, 2003	0	300	0.0	[0.0; 1.2]	4.2%
**Regional Prevalence**		**32912**	**1.6**	**[1.0; 2.3]**	**100.0%**
**Prediction interval**				**[0.0; 6.0]**	

Heterogeneity: I^2 = 94.6% [93.0%; 95.9%], τ^2 = 0.0032, p < 0.0001

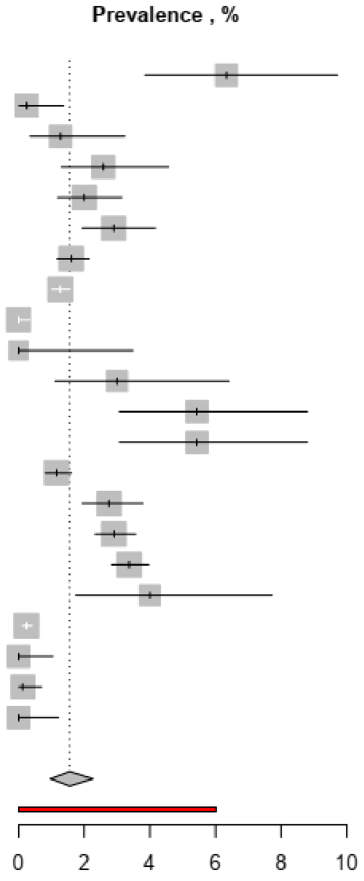

Supplementary Figure 8. Funnel plot for meta-analysis of IgM seroprevalence of Toxoplasma gondii in the global population of pregnant women

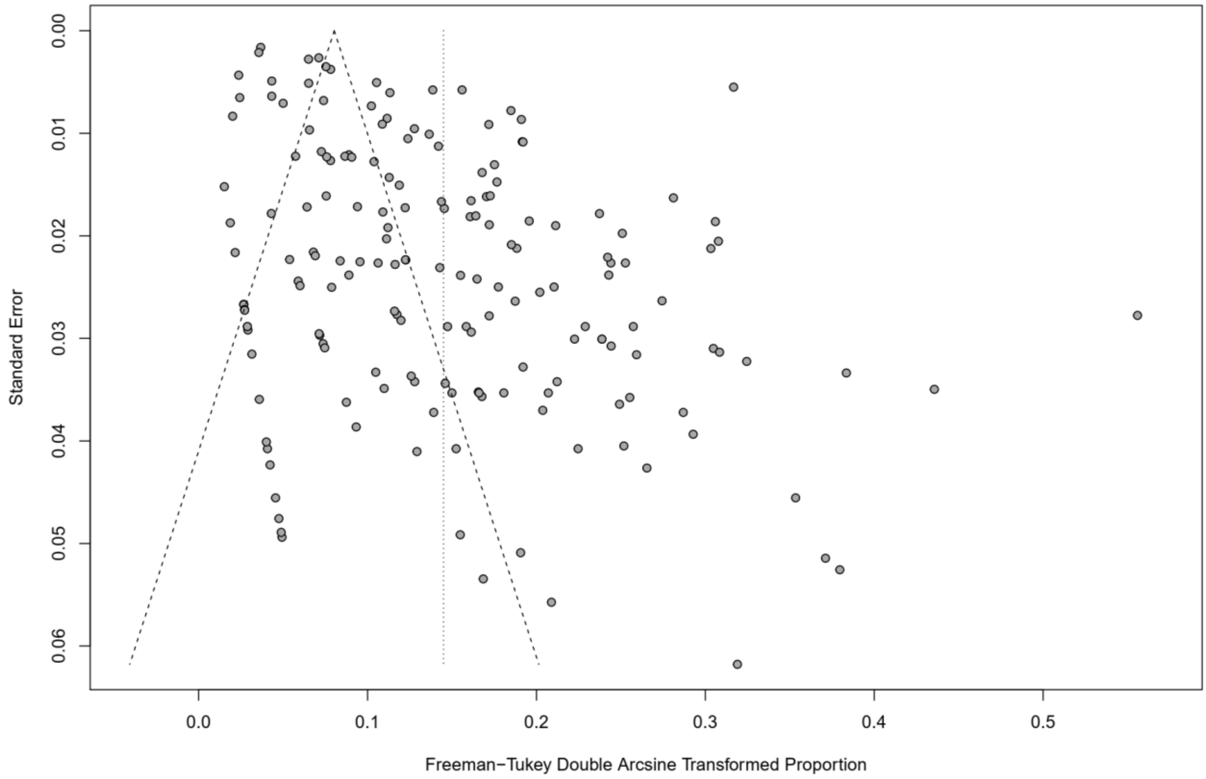

Supplementary Figure 9. Meta-analysis of IgG seroprevalence of Toxoplasma gondii among pregnant women in WHO Africa

Study	Cases	Total	Prevalence, %	[95% C.I.]	Weight
Algeria, Beredjem, 2017	30	143	21.0	[14.6; 28.6]	2.8%
Angola, Lobo, 2017	71	300	23.7	[19.0; 28.9]	2.9%
Benin, Tové, 2018	144	399	36.1	[31.4; 41.0]	2.9%
Benin, Rodier, 1995	113	211	53.6	[46.6; 60.4]	2.9%
Benin, De Paschale, 2014	85	283	30.0	[24.8; 35.7]	2.9%
Burkina Faso, Linguissi, 2012	37	182	20.3	[14.7; 26.9]	2.8%
Burkina Faso, Ouermi, 2009	75	276	27.2	[22.0; 32.8]	2.9%
Burkina Faso, Bamba, 2017	98	316	31.0	[26.0; 36.4]	2.9%
Burkina Faso, Simpore, 2006	35	336	10.4	[7.4; 14.2]	2.9%
Cameroon, Wam, 2016	44	90	48.9	[38.2; 59.7]	2.8%
Cameroon, Njunda, 2011	3	110	2.7	[0.6; 7.8]	2.8%
Cameroon, Ndumbe, 1992	148	192	77.1	[70.5; 82.8]	2.8%
Cameroon, Njunda, 2011	72	110	65.5	[55.8; 74.3]	2.8%
Central African Republic, Gamba, 2013	221	434	50.9	[46.1; 55.7]	2.9%
Congo, Doudou, 2014	17	781	2.2	[1.3; 3.5]	2.9%
Egypt, Eldeeb, 2012	218	323	67.5	[62.1; 72.6]	2.9%
Egypt, El−Nawawy, 1996	0	150	0.0	[0.0; 2.4]	2.8%
Egypt, Kamal, 2015	54	240	22.5	[17.4; 28.3]	2.9%
Ethiopia, Gebremedhin, 2013	176	213	82.6	[76.9; 87.5]	2.9%
Ethiopia, Jemal, 2018	96	401	23.9	[19.8; 28.4]	2.9%
Ethiopia, Endris, 2014	341	385	88.6	[85.0; 91.6]	2.9%
Ethiopia, Awake, 2015	71	384	18.5	[14.7; 22.7]	2.9%
Ethiopia, Abamecha, 2016	191	232	82.3	[76.8; 87.0]	2.9%
Ethiopia, Zemene, 2012	163	201	81.1	[75.0; 86.3]	2.9%
Gabon, Mpiga, 2010	470	839	56.0	[52.6; 59.4]	2.9%
Gabon, Moukandja, 2017	557	973	57.2	[54.1; 60.4]	2.9%
Ghana, Ayi, 2016	64	125	51.2	[42.1; 60.2]	2.8%
Mozambique, Sitoe, 2010	28	150	18.7	[12.8; 25.8]	2.8%
Nigeria, Nasir, 2015	144	360	40.0	[34.9; 45.3]	2.9%
Nigeria, Akinbami, 2010	91	179	50.8	[43.3; 58.4]	2.8%
Rwanda, Murebwayire, 2017	37	384	9.6	[6.9; 13.0]	2.9%
Senegal, Ndiaye, 2011	219	941	23.3	[20.6; 26.1]	2.9%
Tanzania, Mwambe, 2013	108	350	30.9	[26.1; 36.0]	2.9%
Tanzania, Eliakimu, 2018	102	254	40.2	[34.1; 46.5]	2.9%
Zambia, Frimpong, 2017	24	411	5.8	[3.8; 8.6]	2.9%
**Regional Prevalence**		**11658**	**36.5**	**[27.5; 46.0]**	**100.0%**
**Prediction interval**				**[0.1; 90.0]**	

Heterogeneity: I^2 = 99.1% [99.0%; 99.2%], τ^2 = 0.0837, p = 0

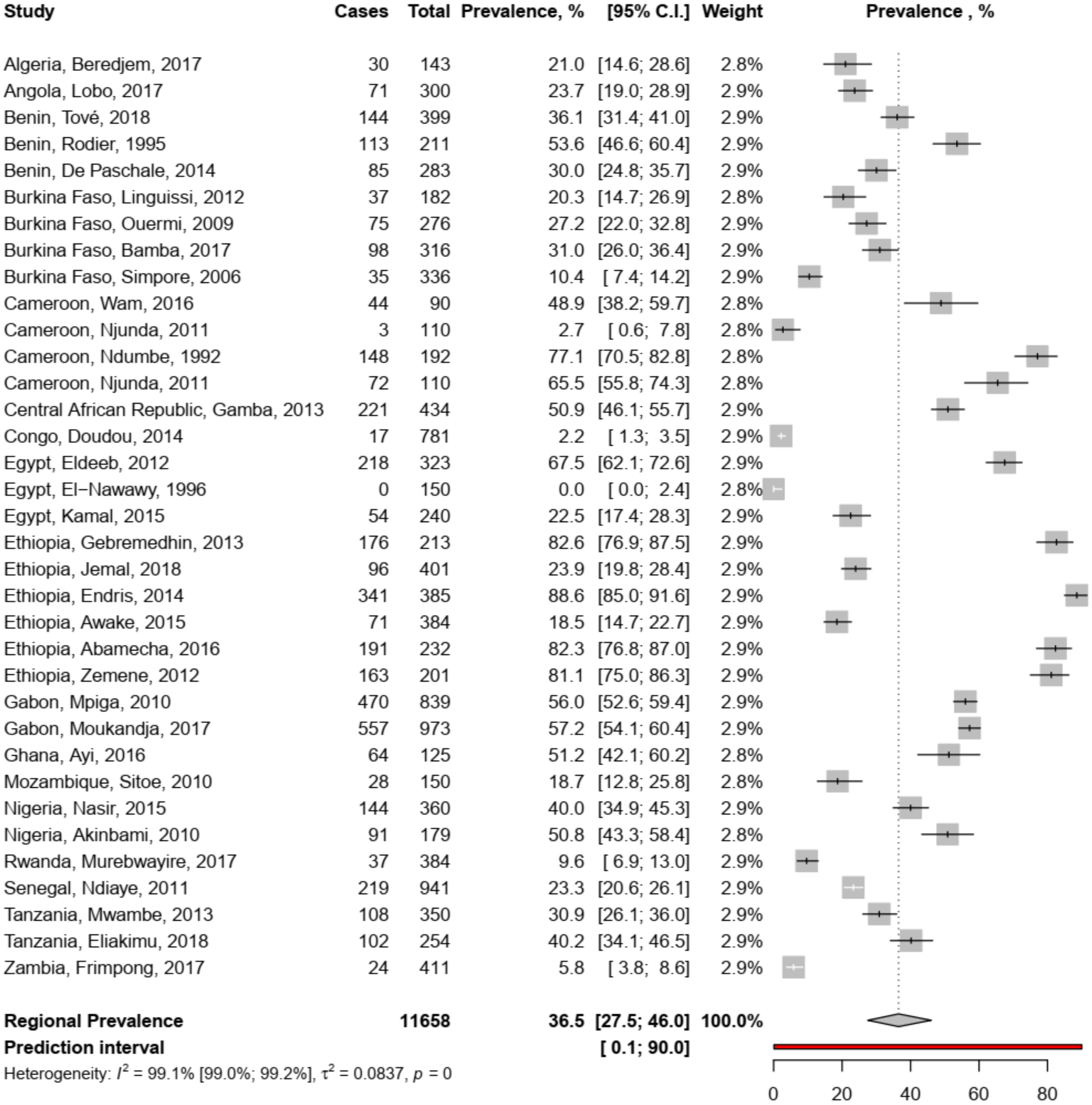

Supplementary Figure 10. Meta-analysis of IgG seroprevalence of Toxoplasma gondii among pregnant women in WHO Americas

Study	Cases	Total	Prevalence, %	[95% C.I.]	Weight
Brazil, Avelar, 2017	362	712	50.8	[47.1; 54.6]	2.1%
Brazil, Da Rocha, 2015	240	338	71.0	[65.9; 75.8]	2.1%
Brazil, Mendez, 2009	38	268	14.2	[10.2; 18.9]	2.1%
Brazil, De Quadros, 2015	24	148	16.2	[10.7; 23.2]	2.1%
Brazil, Cinara, 2011	56	87	64.4	[53.4; 74.4]	2.1%
Brazil, Lopez, 2009	242	492	49.2	[44.7; 53.7]	2.1%
Brazil, Sroka, 2010	661	963	68.6	[65.6; 71.6]	2.1%
Brazil, Dias, 2011	334	607	55.0	[51.0; 59.0]	2.1%
Brazil, Ferezin, 2013	883	1534	57.6	[55.0; 60.1]	2.1%
Brazil, De Moura, 2013	234	400	58.5	[53.5; 63.4]	2.1%
Brazil, Ribeirro, 2008	625	832	75.1	[72.0; 78.0]	2.1%
Brazil, Reis, 2006	6125	10408	58.8	[57.9; 59.8]	2.1%
Brazil, Santoz, 1995	484	698	69.3	[65.8; 72.7]	2.1%
Brazil, Costa, 2018	525	726	72.3	[68.9; 75.5]	2.1%
Brazil, Dasilva, 2014	335	487	68.8	[64.5; 72.9]	2.1%
Brazil, Fonseca, 2012	1057	2136	49.5	[47.3; 51.6]	2.1%
Brazil, Spaldy, 2005	1583	2126	74.5	[72.5; 76.3]	2.1%
Brazil, Vaz, 2010	2638	20389	12.9	[12.5; 13.4]	2.1%
Brazil, Lopes, 2013	1151	2226	51.7	[49.6; 53.8]	2.1%
Brazil, Varella, 2000	724	1261	57.4	[54.6; 60.2]	2.1%
Brazil, Sartori, 2011	6910	10316	67.0	[66.1; 67.9]	2.1%
Brazil, Varella, 2009	241	41112	0.6	[0.5; 0.7]	2.1%
Brazil, Fochi, 2015	120	213	56.3	[49.4; 63.1]	2.1%
Brazil, Freitas, 2017	210	356	59.0	[53.7; 64.1]	2.1%
Brazil, Isabelle Ribeiro, 2009	126	190	66.3	[59.1; 73.0]	2.1%
Brazil, Vaz, 1990	67	481	13.9	[11.0; 17.3]	2.1%
Brazil, Reiche, 2000	1044	1559	67.0	[64.6; 69.3]	2.1%
Brazil, Gonçalves, 2010	345	574	60.1	[56.0; 64.1]	2.1%
Brazil, Inagaki, 2009	6615	9550	69.3	[68.3; 70.2]	2.1%
Brazil, Bittencourt, 2012	2405	4021	59.8	[58.3; 61.3]	2.1%
Brazil, Samara, 2015	427	551	77.5	[73.8; 80.9]	2.1%
Brazil, Mioranza, 2008	183	334	54.8	[49.3; 60.2]	2.1%
Brazil, Rebouças, 2011	1297	2229	58.2	[56.1; 60.2]	2.1%
Canada, Sekla, 1981	129	55527	0.2	[0.2; 0.3]	2.1%
Chile, Vial, 1985	485	1304	37.2	[34.6; 39.9]	2.1%
Chile, Contreras, 1995	43	139	30.9	[23.4; 39.3]	2.1%
Colombia, Cardenas, 2011	52	167	31.1	[24.2; 38.8]	2.1%
Colombia, Rosso, 2008	437	955	45.8	[42.6; 49.0]	2.1%
Colombia, Angel−Nueller, 2011	637	2258	28.2	[26.4; 30.1]	2.1%
Colombia, Barrera, 2002	141	301	46.8	[41.1; 52.7]	2.1%
Grenada, Asthana, 2006	304	534	56.9	[52.6; 61.2]	2.1%
Mexico, Alvarado−Esquivel, 2009	36	439	8.2	[5.8; 11.2]	2.1%
Mexico, Alvarado, 2006	21	343	6.1	[3.8; 9.2]	2.1%
Trinidad & Tobago, Ramsewak, 2008	83	232	35.8	[29.6; 42.3]	2.1%
USA, Hershey, 1987	4	120	3.3	[0.9; 8.3]	2.1%
Venezuela, Diaz−Suarez, 2009	33	100	33.0	[23.9; 43.1]	2.1%
Venezuela, De Noya, 2010	258	678	38.1	[34.4; 41.8]	2.1%
**Regional Prevalence**		**181421**	**45.2**	**[33.4; 57.3]**	**100.0%**
**Prediction interval**				**[0.0; 100.0]**	

Heterogeneity: I^2 = 100.0% [100.0%; 100.0%], τ^2 = 0.1790, p = 0

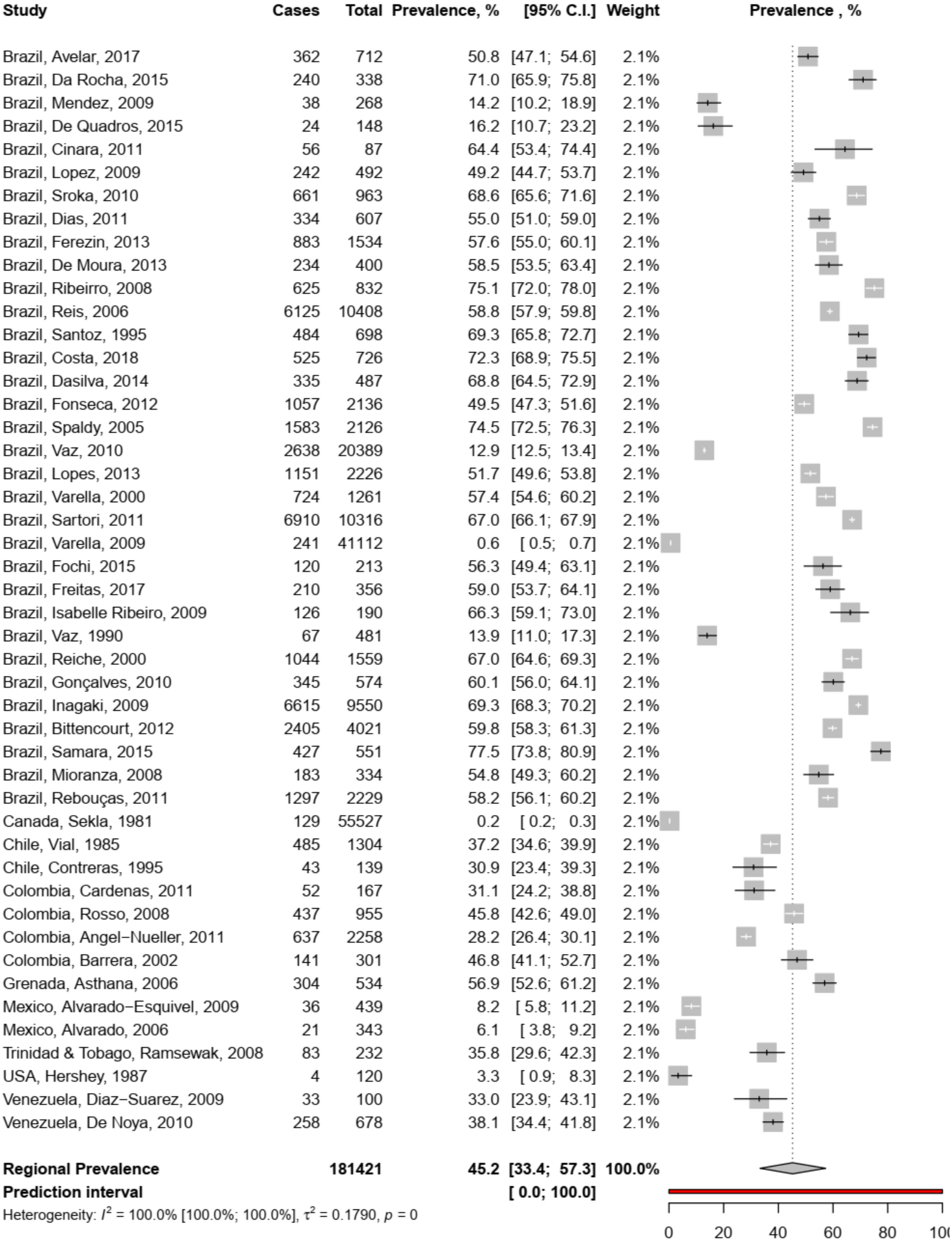

Supplementary Figure 11. Meta-analysis of IgG seroprevalence of Toxoplasma gondii among pregnant women in WHO Eastern Mediterranean

Study	Cases	Total	Prevalence, %	[95% C.I.]	Weight
Iran, Abdi, 2008	247	533	46.3	[42.0; 50.7]	2.2%
Iran, Abolghasem, 2011	99	225	44.0	[37.4; 50.8]	2.2%
Iran, Ahmadpour, 2015	66	264	25.0	[19.9; 30.7]	2.2%
Iran, Akhlaghi, 2016	86	468	18.4	[15.0; 22.2]	2.2%
Iran, Babaie, 2013	144	419	34.4	[29.8; 39.1]	2.2%
Iran, Esboei, 2016	249	786	31.7	[28.4; 35.1]	2.2%
Iran, Garedaghi, 2017	79	200	39.5	[32.7; 46.6]	2.2%
Iran, Hajsoleimani, 2012	186	500	37.2	[33.0; 41.6]	2.2%
Iran, Hoseini, 2014	170	289	58.8	[52.9; 64.6]	2.2%
Iran, Josheghani, 2015	30	80	37.5	[26.9; 49.0]	2.0%
Iran, Mohammad, 2011	250	721	34.7	[31.2; 38.3]	2.2%
Iran, Mousavi, 2018	28	110	25.5	[17.6; 34.6]	2.1%
Iran, Naghili, 2017	202	391	51.7	[46.6; 56.7]	2.2%
Iran, Nourollahpour, 2016	194	360	53.9	[48.6; 59.1]	2.2%
Iran, Rajaii, 2013	898	1659	54.1	[51.7; 56.5]	2.2%
Iran, Saki, 2016	71	220	32.3	[26.1; 38.9]	2.2%
Iran, Shamakhteh, 2016	93	150	62.0	[53.7; 69.8]	2.1%
Iran, Sharbatkhori, 2014	221	555	39.8	[35.7; 44.0]	2.2%
Iran, Shieh, 2017	85	261	32.6	[26.9; 38.6]	2.2%
Iran, Shirdel, 2017	155	440	35.2	[30.8; 39.9]	2.2%
Iran, Tabatabaie, 2015	58	200	29.0	[22.8; 35.8]	2.2%
Iran, Varaghchi, 2015	485	519	93.4	[91.0; 95.4]	2.2%
Iran, Yad, 2014	137	501	27.3	[23.5; 31.5]	2.2%
Jordan, Jumaian, 2005	133	280	47.5	[41.5; 53.5]	2.2%
Kuwait, Iqbal, 2007	119	224	53.1	[46.4; 59.8]	2.2%
Lebanon, Hasan, 2017	1965	2456	80.0	[78.4; 81.6]	2.2%
Morocco, Laboud, 2017	54	128	42.2	[33.5; 51.2]	2.1%
Morocco, Mansouri, 2007	1242	2456	50.6	[48.6; 52.6]	2.2%
Morocco, Tlamcani, 2017	1367	3440	39.7	[38.1; 41.4]	2.2%
Pakistan, Bari, 1990	149	324	46.0	[40.5; 51.6]	2.2%
Pakistan, Majid, 2016	135	733	18.4	[15.7; 21.4]	2.2%
Palestine, Nigem, 2009	57	204	27.9	[21.9; 34.6]	2.2%
Saudi Arabia, Almogren, 2011	825	2176	37.9	[35.9; 40.0]	2.2%
Saudi Arabia, Almushait, 2014	189	487	38.8	[34.5; 43.3]	2.2%
Saudi Arabia, Aqueely, 2014	39	195	20.0	[14.6; 26.3]	2.1%
Saudi Arabia, Bin, 2012	53	137	38.7	[30.5; 47.4]	2.1%
Saudi Arabia, Elsafi, 2015	104	400	26.0	[21.8; 30.6]	2.2%
Saudi Arabia, Imam, 2016	32	150	21.3	[15.1; 28.8]	2.1%
Saudi Arabia, Ismail, 2014	28	96	29.2	[20.3; 39.3]	2.1%
Saudi Arabia, Jawahir, 2016	81	250	32.4	[26.6; 38.6]	2.2%
Saudi Arabia, Mohamed, 2016	69	326	21.2	[16.9; 26.0]	2.2%
Saudi Arabia, Mohammad, 2013	285	554	51.4	[47.2; 55.7]	2.2%
Sudan, Elnahas, 2003	166	487	34.1	[29.9; 38.5]	2.2%
Sudan, Maha, 2012	70	188	37.2	[30.3; 44.6]	2.1%
Yemen, Al−Eryani, 2016	259	593	43.7	[39.6; 47.8]	2.2%
Yemen, Mahdy, 2017	166	359	46.2	[41.0; 51.5]	2.2%
**Regional Prevalence**		**26494**	**39.7**	**[34.5; 45.0]**	**100.0%**
**Prediction interval**				**[9.2; 75.6]**	

Heterogeneity: $I^2 = 98.7\%$ [98.5%; 98.8%], $\tau^2 = 0.0332$, $p = 0$

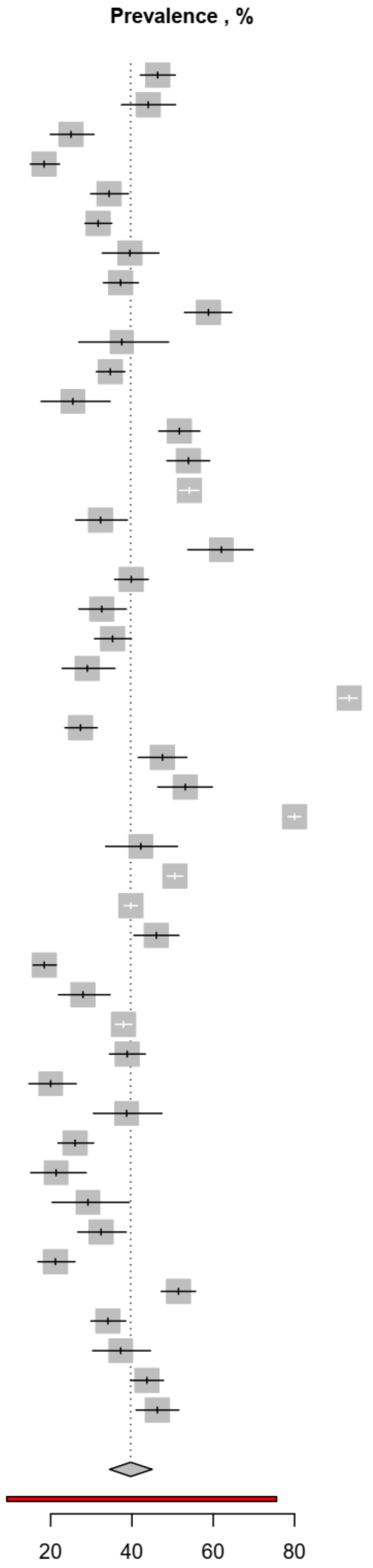

Supplementary Figure 12. Meta-analysis of IgG seroprevalence of Toxoplasma gondii among pregnant women in WHO Europe

Study	Cases	Total	Prevalence, %	[95% C.I.]	Weight
Albania, Abazaj, 2016	48	152	31.6	[24.3; 39.6]	1.5%
Belgium, Luyasu, 1993	389	830	46.9	[43.4; 50.3]	1.6%
Croatia, Vilibic−Cavlek, 2011	146	502	29.1	[25.1; 33.3]	1.6%
Croatia, Punda−Polic, 2000	125	398	31.4	[26.9; 36.2]	1.6%
Cyprus, Marios, 2016	3065	17631	17.4	[16.8; 18.0]	1.6%
Denmark, Lebech, 1993	1480	5402	27.4	[26.2; 28.6]	1.6%
Finland, Maija, 1992	3397	16733	20.3	[19.7; 20.9]	1.6%
France, Franck, 2008	97	200	48.5	[41.4; 55.7]	1.5%
Germany, Freise, 1993	185	512	36.1	[32.0; 40.5]	1.6%
Iceland, Iddawela, 2017	160	536	29.9	[26.0; 33.9]	1.6%
Italy, Valcavi, 1995	1687	3602	46.8	[45.2; 48.5]	1.6%
Italy, Maggi, 2008	241	496	48.6	[44.1; 53.1]	1.6%
Italy, Pucio, 2014	154	846	18.2	[15.7; 21.0]	1.6%
Italy, Dentico, 2011	97	334	29.0	[24.2; 34.2]	1.6%
Italy, Depaschale, 2008	737	3426	21.5	[20.1; 22.9]	1.6%
Italy, Ruffini, 2014	2723	10085	27.0	[26.1; 27.9]	1.6%
Italy, Capretti, 2014	2308	10867	21.2	[20.5; 22.0]	1.6%
Italy, Leone, 1996	270	1668	16.2	[14.5; 18.0]	1.6%
Norway, Jenum, 1998	3907	35940	10.9	[10.6; 11.2]	1.6%
Poland, Radon−Pokracka, 2017	83	440	18.9	[15.3; 22.8]	1.6%
Poland, Marcinek, 2008	910	1800	50.6	[48.2; 52.9]	1.6%
Poland, Nowakowska, 2013	3364	8281	40.6	[39.6; 41.7]	1.6%
Poland, Niemiec, 2002	722	2016	35.8	[33.7; 38.0]	1.6%
Poland, Nowakowska, 2001	387	1920	20.2	[18.4; 22.0]	1.6%
Portugal, Lobo, 2017	17	155	11.0	[6.5; 17.0]	1.5%
Portugal, Lito, 2013	804	3162	25.4	[23.9; 27.0]	1.6%
Russia, Boronina, 2018	17	65	26.2	[16.0; 38.5]	1.4%
Spain, Ribes, 1996	39	299	13.0	[9.4; 17.4]	1.6%
Spain, Ramois, 2011	619	1627	38.0	[35.7; 40.5]	1.6%
Spain, Lopez−Fabal, 2013	1874	8012	23.4	[22.5; 24.3]	1.6%
Spain, Jaqueti, 1991	475	1221	38.9	[36.2; 41.7]	1.6%
Spain, Guerra, 1995	49	191	25.7	[19.6; 32.5]	1.5%
Sweden, Ahlfors, 1989	1740	4351	40.0	[38.5; 41.5]	1.6%
Sweden, Evengard, 1999	450	3094	14.5	[13.3; 15.8]	1.6%
Switzerland, Jacquier, 1995	4176	9059	46.1	[45.1; 47.1]	1.6%
Turkey, Durdu, 2017	51	102	50.0	[39.9; 60.1]	1.5%
Turkey, Gundem, 2014	127	419	30.3	[25.9; 35.0]	1.6%
Turkey, Gencer, 2014	56	196	28.6	[22.4; 35.4]	1.5%
Turkey, Dogan, 2014	528	1709	30.9	[28.7; 33.1]	1.6%
Turkey, Aysun, 2011	408	1102	37.0	[34.2; 40.0]	1.6%
Turkey, Aynioglu, 2015	400	910	44.0	[40.7; 47.3]	1.6%
Turkey, Harma, 2004	694	1149	60.4	[57.5; 63.2]	1.6%
Turkey, Tamer, 2009	960	1972	48.7	[46.5; 50.9]	1.6%
Turkey, Uysal, 2013	1871	4651	40.2	[38.8; 41.7]	1.6%
Turkey, Okyay, 2013	1910	3340	57.2	[55.5; 58.9]	1.6%
Turkey, Mumtaz, 2017	2427	7513	32.3	[31.2; 33.4]	1.6%
Turkey, Ertug, 2005	117	389	30.1	[25.6; 34.9]	1.6%
Turkey, Ozekinci, 2005	305	879	34.7	[31.6; 37.9]	1.6%
Turkey, Karacan, 2014	291	1258	23.1	[20.8; 25.6]	1.6%
Turkey, Cetin, 2017	5631	11564	48.7	[47.8; 49.6]	1.6%
Turkey, Beytur, 2010	63	205	30.7	[24.5; 37.5]	1.5%
Turkey, Ocak, 2007	860	1652	52.1	[49.6; 54.5]	1.6%
Turkey, Mumcuoglu, 2014	1278	6140	20.8	[19.8; 21.9]	1.6%
Turkey, Fusun, 2011	426	1646	25.9	[23.8; 28.1]	1.6%
Turkey, Toklu, 2013	268	1465	18.3	[16.3; 20.4]	1.6%
Turkey, Parlak, 2015	3688	9809	37.6	[36.6; 38.6]	1.6%
Turkey, Çekin, 2011	1358	7520	18.1	[17.2; 18.9]	1.6%
Turkey, Ustaçelebi, 1986	144	301	47.8	[42.1; 53.6]	1.6%
Turkey, Selek, 2015	421	1737	24.2	[22.2; 26.3]	1.6%
UK, Allain, 1998	387	13328	2.9	[2.6; 3.2]	1.6%
UK, Gilbert, 1992	1267	6749	18.8	[17.8; 19.7]	1.6%
UK, Flatt, 2013	452	2610	17.3	[15.9; 18.8]	1.6%
UK, Nash, 2005	172	1897	9.1	[7.8; 10.4]	1.6%
**Regional Prevalence**		**248065**	**30.0**	**[26.2; 33.9]**	**100.0%**
**Prediction interval**				**[5.6; 63.4]**	

Heterogeneity: I^2 = 99.8% [99.8%; 99.8%], τ^2 = 0.0285, p = 0

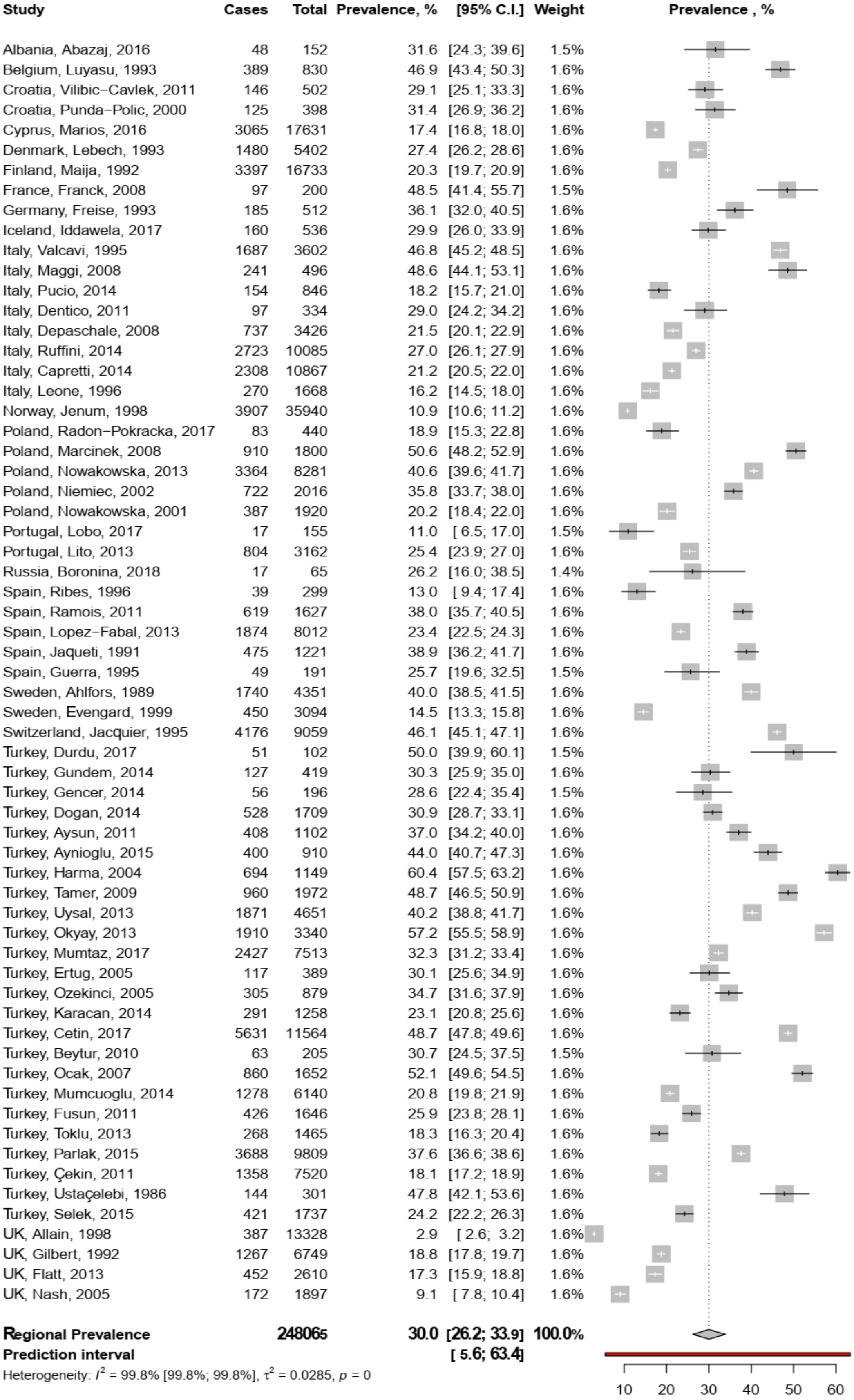

Supplementary Figure 13. Meta-analysis of IgG seroprevalence of Toxoplasma gondii among pregnant women in WHO South-East Asia

Study	Cases	Total	Prevalence, %	[95% C.I.]	Weight
Bangladesh, Ashrafunnessa, 1998	110	286	38.5	[32.8; 44.4]	5.9%
India, Amar, 2015	26	103	25.2	[17.2; 34.8]	5.4%
India, Borkakoty, 2007	75	180	41.7	[34.4; 49.2]	5.7%
India, Borkakoty, 2016	548	1141	48.0	[45.1; 51.0]	6.1%
India, Iqbal, 2010	216	800	27.0	[24.0; 30.2]	6.1%
India, Khurana, 2010	46	300	15.3	[11.4; 19.9]	5.9%
India, Ravindanath, 2012	35	94	37.2	[27.5; 47.8]	5.3%
India, Selvaraj, 2017	30	193	15.5	[10.7; 21.4]	5.7%
India, Singh, 2016	40	260	15.4	[11.2; 20.4]	5.9%
India, Singh, 2004	78	300	26.0	[21.1; 31.4]	5.9%
India, Yasmeen, 2017	53	251	21.1	[16.2; 26.7]	5.8%
Sri Lanka, Chandrenasa, 2016	36	293	12.3	[8.8; 16.6]	5.9%
Thailand, Andiappan, 2014	167	760	22.0	[19.1; 25.1]	6.1%
Thailand, Chintana, 1998	155	1181	13.1	[11.3; 15.2]	6.1%
Thailand, Nissapatom, 2011	138	640	21.6	[18.4; 25.0]	6.1%
Thailand, Sukthana, 1999	63	300	21.0	[16.5; 26.1]	5.9%
Thailand, Wanachiwanawi, 2001	494	1669	29.6	[27.4; 31.9]	6.1%
**Regional Prevalence**		**8751**	**24.6**	**[19.4; 30.2]**	**100.0%**
**Prediction interval**				**[5.4; 51.7]**	

Heterogeneity: $I^2 = 97.0\%$ [96.1%; 97.7%], $\tau^2 = 0.0165$, $p < 0.0001$

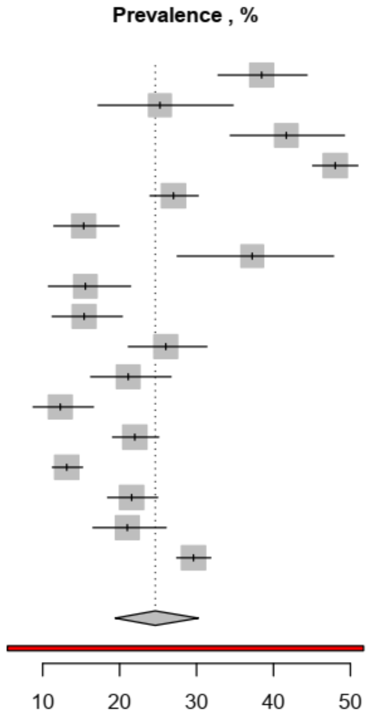

Supplementary Figure 14. Meta-analysis of IgG seroprevalence of Toxoplasma gondii among pregnant women in Western Pacific

Study	Cases	Total	Prevalence, %	[95% C.I.]	Weight
Australia, Karunajeewa, 2001	72	308	23.4	[18.8; 28.5]	4.0%
Australia, Sukthana, 1999	90	300	30.0	[24.9; 35.5]	4.0%
China, Chen, 2014	156	2993	5.2	[4.4; 6.1]	4.1%
China, Chou, 2011	20	200	10.0	[6.2; 15.0]	3.9%
China, Cong, 2015	147	965	15.2	[13.0; 17.7]	4.1%
China, Gao, 2017	37	404	9.2	[6.5; 12.4]	4.0%
China, Hua, 2013	43	1081	4.0	[2.9; 5.3]	4.1%
China, Hung, 2015	7	104	6.7	[2.7; 13.4]	3.7%
China, Jiang, 2018	43	313	13.7	[10.1; 18.1]	4.0%
China, Lin, 2008	132	426	31.0	[26.6; 35.6]	4.0%
China, Liu, 2009	25	235	10.6	[7.0; 15.3]	3.9%
China, Lui, 2014	1218	9139	13.3	[12.6; 14.0]	4.1%
China, Qin, 2011	52	1307	4.0	[3.0; 5.2]	4.1%
China, Xia, 2011	351	6849	5.1	[4.6; 5.7]	4.1%
China, Yang, 2016	32	276	11.6	[8.1; 16.0]	3.9%
China, Yanping, 2014	151	2740	5.5	[4.7; 6.4]	4.1%
China, Zhou, 2015	11	3014	0.4	[0.2; 0.7]	4.1%
Malaysia, Emelia, 2014	94	281	33.5	[28.0; 39.3]	4.0%
Malaysia, Nissapatorn, 2003	78	200	39.0	[32.2; 46.1]	3.9%
New Zealand, Morris, 2004	1063	5000	21.3	[20.1; 22.4]	4.1%
Papua New Guinea, Klufio, 1993	35	197	17.8	[12.7; 23.8]	3.9%
South Korea, Gye−Cheol, 2011	18	787	2.3	[1.4; 3.6]	4.1%
South Korea, Han, 2008	14	351	4.0	[2.2; 6.6]	4.0%
South Korea, Song, 2005	51	5925	0.9	[0.6; 1.1]	4.1%
Vietnam, Buchy, 2003	34	300	11.3	[8.0; 15.5]	4.0%
**Regional Prevalence**		**43695**	**11.2**	**[7.7; 15.2]**	**100.0%**
**Prediction interval**				**[0.0; 37.2]**	

Heterogeneity: I^2 = 99.3% [99.2%; 99.4%], τ^2 = 0.0222, p = 0

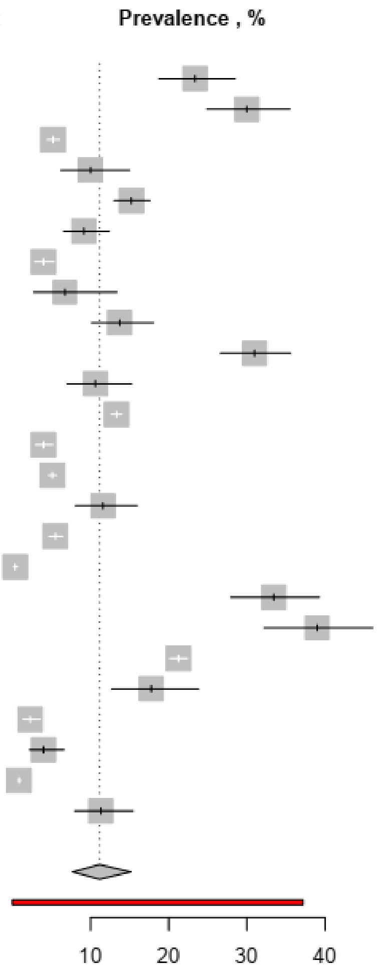

Supplementary Figure 15. Funnel plot for meta-analysis of IgG seroprevalence of Toxoplasma gondii in the global population of pregnant women

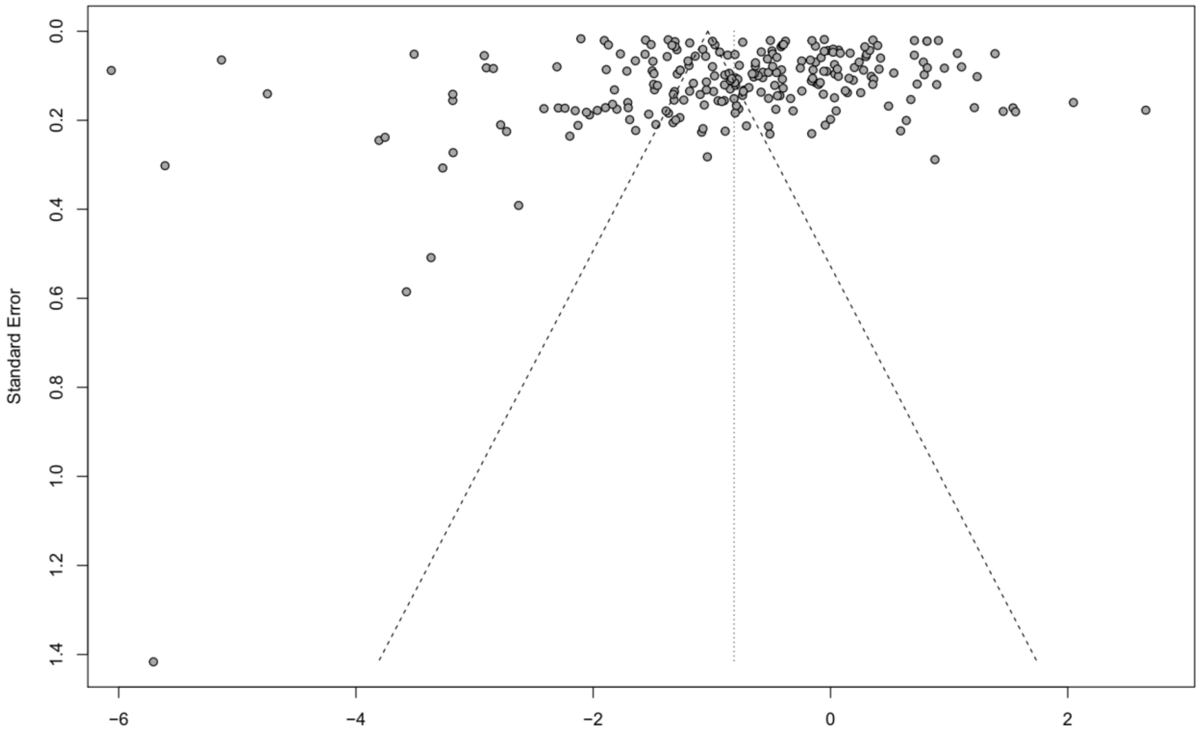

Reference list of included studies

1-25 26-50 51-75 76-100 101-125 126-150 151-175 176-200 201-225 226-233

1. Abamecha F, Awel H. Seroprevalence and risk factors of Toxoplasma gondii infection in pregnant women following antenatal care at Mizan Aman General Hospital, Bench Maji Zone (BMZ), Ethiopia. *BMC Infectious Diseases* 2016; **16**(1).

2. Abazaj E, Deliu EF, Petri O, et al. IgG AVIDITY TEST FOR DIAGNOSIS OF ACUTE Toxoplasma gondii IN PREGNANT WOMEN. *Journal of Environmental Protection and Ecology* 2016; **17**(2): 681-6.

3. Ben Abdallah R, Siala E, Bouafsoun A, et al. Toxoplasmosis mother-to-child screening: Study of cases followed in the Pasteur Institute of Tunis (2007-2010). *Bulletin de la Societe de Pathologie Exotique* 2013; **106**(2): 108-12.

4. Abdi J, Shojaee S, Mirzaee A, Keshavarz H. Seroprevalence of toxoplasmosis in pregnant women in Ilam Province, Iran. *Iranian Journal of Parasitology* 2008; **3**(2): 34-7.

5. Abolghasem SP, Bonyadi MR, Babaloo Z, et al. IgG avidity test for the diagnosis of acute toxoplasma gondii infection in early pregnancy. *Iranian Journal of Immunology* 2011; **8**(4): 251-5.

6. Ahlfors K, Borjeson M, Huldt G, Forsberg E. Incidence of toxoplasmosis in pregnant women in the city of Malmo, Sweden. *Scandinavian Journal of Infectious Diseases* 1989; **21**(3): 315-21.

7. Ahmadpour GR, Ezatpour B, Hadighi R, et al. Seroepidemiology of Toxoplasma gondii infection in pregnant women in west Iran: determined by ELISA and PCR analysis. *Journal of Parasitic Diseases* 2017; **41**(1): 237-42.

8. Akhlaghi L, Tabatabaie F, Hadighi R, et al. Diagnosis of acute toxoplasmosis in pregnant women referred to therapeutic centers of Alborz Province (Iran) using immunoglobulin G avidity ELISA technique. *Asian Pacific Journal of Tropical Disease* 2016; **6**(11): 864-7.

9. Akinbami AA, Adewunmi AA, Rabiu KA, et al. Seroprevalence of Toxoplasma gondii antibodies amongst pregnant women at the Lagos State University Teaching Hospital, Nigeria. *The Nigerian postgraduate medical journal* 2010; **17**(2): 164-7.

10. Al-Eryani SMA, Al-Mekhlafi AM, Al-Shibani LA, Mahdy MMK, Azazy AA. Toxoplasma gondii infection among pregnant women in Yemen: Factors associated with high seroprevalence. *Journal of Infection in Developing Countries* 2016; **10**(6): 667-72.

11. Ali LF, Mohanned S, Talib M. Seroprevalence of anti-toxplasma gondii, anti-rubella, anti-cytomegalovirus and anti-herpes simplex IgM antibodies in pregnant women in Baghdad. *Pakistan Journal of Biotechnology* 2017; **14**(4): 785-90.

12. Allain JP, Palmer CR, Pearson G. Epidemiological study of latent and recent infection by Toxoplasma gondii in pregnant women from a regional population in the U.K. *The Journal of infection* 1998; **36**(2): 189-96.

13. Almogren A. Antenatal screening for Toxoplasma gondii infection at a tertiary care hospital in Riyadh, Saudi Arabia. *Annals of Saudi Medicine* 2011; **31**(6): 569-72.

14. Almushait MA, Dajem SMB, Elsherbiny NM, Eskandar MA, Al Azraqi TA, Makhlouf LM. Seroprevalence and risk factors of Toxoplasma gondii infection among pregnant women in south western, Saudi Arabia. *Journal of Parasitic Diseases* 2014; **38**(1): 4-10.

15. Alvarado-Esquivel C, Sifuentes-Alvarez A, Narro-Duarte SG, et al. Seroepidemiology of Toxoplasma gondii infection in pregnant women in a public hospital in northern Mexico. *BMC infectious diseases* 2006; **6**.

16. Alvarado-Esquivel C, Torres-Castorena A, Liesenfeld O, et al. Seroepidemiology of Toxoplasma gondii infection in pregnant women in rural durango, Mexico. *Journal of Parasitology* 2009; **95**(2): 271-4.

17. Amar OAO, Bajaj HK, Peter JK, Masih H. Toxoplasmosis prevalence in pregnant women of plain gangetic region, Allahabad. *Journal of Pure and Applied Microbiology* 2015; **9**(2): 1611-9.

18. Andiappan H, Nissapatorn V, Sawangjaroen N, et al. Toxoplasma infection in pregnant women: A current status in Songklanagarind hospital, southern Thailand. *Parasites and Vectors* 2014; **7**(1).

19. Angel-Müller E, Hougton MP, Eslava C, Riaño J, Rey GE, Gómez-Marín JE. Gestational and congenital toxoplasmosis in two hospitals in Bogota, Colombia. *Rev Fac Med Univ Nac Colomb* 2014; **62**(2): 179-85.

20. Aqeely H, El-Gayar EK, Perveen Khan D, et al. Seroepidemiology of toxoplasma gondii amongst pregnant women in Jazan Province, Saudi Arabia. *Journal of Tropical Medicine* 2014; **2014**.

21. Ashrafunnessa, Khatun S, Nazrul Islam M, Huq T. Seroprevalence of toxoplasma antibodies among the antenatal population in Bangladesh. *Journal of Obstetrics and Gynaecology Research* 1998; **24**(2): 115-9.

22. Asthana SP, Macpherson CNL, Weiss SH, et al. Seroprevalence of Toxoplasma gondii in pregnant women and cats in Grenada, West Indies. *Journal of Parasitology* 2006; **92**(3): 644-5.

23. Avelar MV, Martinez VO, de Moura DL, et al. Association between seroprevalence of IgG anti-Toxoplasma gondii and risk factors for infection among pregnant women in climério de oliveira maternity, Salvador, Bahia, Brazil. *Revista do Instituto de Medicina Tropical de Sao Paulo* 2017; **59**.

24. Awoke K, Nibret E, Munshea A. Sero-prevalence and associated risk factors of Toxoplasma gondii infection among pregnant women attending antenatal care at Felege Hiwot Referral Hospital, northwest Ethiopia. *Asian Pacific Journal of Tropical Medicine* 2015; **8**(7): 547-52.

25. Ayi I, Sowah AOK, Blay EA, Suzuki T, Ohta N, Ayeh-Kumi PF. Toxoplasma gondii infections among pregnant women, children and HIV-seropositive persons in Accra, Ghana. *Tropical Medicine and Health* 2016; **44**(1).

26. Aynioglu A, Aynioglu O, Altunok ES. Seroprevalence of Toxoplasma gondii, rubella and Cytomegalovirus among pregnant females in north-western Turkey. *Acta clinica Belgica* 2015; **70**(5): 321-4.

27. Karabulut A, Polat Y, Turk M, Balci YI. Evaluation of rubella, Toxoplasma gondii, and cytomegalovirus seroprevalences among pregnant women in Denizli province. *Turkish Journal of Medical Sciences* 2011; **41**(1): 159-64.

28. Babaie J, Amiri S, Mostafavi E, et al. Seroprevalence and risk factors for toxoplasma gondii infection among pregnant women in northeast Iran. *Clinical and Vaccine Immunology* 2013; **20**(11): 1771-3.

29. Bamba S, Cissé M, Sangaré I, Zida A, Ouattara S, Guiguemdé RT. Seroprevalence and risk factors of Toxoplasma gondii infection in pregnant women from Bobo Dioulasso, Burkina Faso. *BMC Infectious Diseases* 2017; **17**(1).

30. Bari A, Khan QA. Toxoplasmosis among pregnant women in northern parts of Pakistan. *JPMA The Journal of the Pakistan Medical Association* 1990; **40**(12): 288-9.

31. Barrera AM, Castiblanco P, GÓMez M JE, et al. Toxoplasmosis Adquirida Durante el Embarazo, en el Instituto Materno Infantil en Bogotá. *Revista de Salud Pública* 2002; **4**(3): 286-93.

32. Beach PG. Prevalence of antibodies to Toxoplasma gondii in pregnant women in Oregon. *Journal of Infectious Diseases* 1979; **140**(5): 780-3.

33. Berredjem H, Aouras H, Benlaifa M, Becheker I, Djebar MR. Contribution of IgG avidity and PCR for the early diagnosis of toxoplasmosis in pregnant women from the North-Eastern region of Algeria. *African Health Sciences* 2017; **17**(3): 647-56.

34. Beytur L, Iraz M, Karadan M, et al. Toxoplasma seropositivity in a state hospital for a year. *Marmara Medical Journal* 2010; **23**(3): 347-52.

35. Bin Dajem SM, Almushait MA. Detection of toxoplasma gondii DNA by PCR in blood samples collected from pregnant saudi women from the aseer region, Saudi Arabia. *Annals of Saudi Medicine* 2012; **32**(5): 507-12.

36. Bittencourt LHFB, Lopes-Mori FMR, Mitsuka-Breganó R, et al. Seroepidemiology of toxoplasmosis in pregnant women since the implementation of the surveillance program of toxoplasmosis acquired in pregnancy and congenital in the western region of Paraná, Brazil. *Revista Brasileira de Ginecologia e Obstetricia* 2012; **34**(2): 63-8.

37. Boa-Sorte N, Purificação A, Amorim T, Assunção L, Reis A, Galvão-Castro B. Dried blood spot testing for the antenatal screening of HTLV, HIV, syphilis, toxoplasmosis and hepatitis B and C: prevalence, accuracy and operational aspects. *Braz J Infect Dis* 2014; **18**(6): 618-24.

38. Borkakoty B, Borthakur A, Gohain M. Prevalence of Toxoplasma gondii infection amongst pregnant women in Assam, India [8]. *Indian Journal of Medical Microbiology* 2007; **25**(4): 431-2.

39. Borkakoty B, Biswas D, Jakharia A, Mahanta J. Seroprevalence of Toxoplasma gondii Among Pregnant Women in Northeast India. *The Journal of the Association of Physicians of India* 2016; **64**(10): 24-8.

40. Boronina LG, Blinova SM, Samatova EV. The serological diagnostic of toxoplasmosis in children and pregnant women using immune chemical technique by test-systems of various manufacturers. *Klinicheskaia laboratornaia diagnostika* 2018; **63**(1): 41-4.

41. Buchy P, Follézou JY, Lien TX, et al. Serological study of toxoplasmosis in Vietnam in a population of drug users (Ho Chi Minh city) and pregnant women (Nha Trang). *Bulletin de la Société de pathologie exotique (1990)* 2003; **96**(1): 46-7.

42. Capretti MG, De Angelis M, Tridapalli E, et al. Toxoplasmosis in pregnancy in an area with low seroprevalence: Is prenatal screening still worthwhile? *Pediatric Infectious Disease Journal* 2014; **33**(1): 5-10.

43. Cárdenas D, Lozano C, Castillo Z, et al. Frecuencia de anticuerpos anti Toxoplasma gondii en gestantes de Cúcuta, Colombia. *Revista Medica Herediana* 2015; **26**(4): 230-7.

44. Çekin Y, Kizilateş F, Gür N, Senol Y. [Investigation of Toxoplasma gondii seropositivity in pregnant women attending the Antalya Training and Research Hospital for the last four years]. *Türkiye parazitolojii dergisi / Türkiye Parazitoloji Derneği = Acta parasitologica Turcica / Turkish Society for Parasitology* 2011; **35**(4): 181-4.

45. Çetin M, Çetin Ş. Age-related prevalence of toxoplasmosis among pregnant women in Hatay: Estimation depending on model. *Mikrobiyoloji bulteni* 2017; **51**(4): 361-9.

46. Chandrasena N, Herath R, Rupasinghe N, et al. Toxoplasmosis awareness, seroprevalence and risk behavior among pregnant women in the Gampaha district, Sri Lanka. *Pathogens and Global Health* 2016; **110**(2): 62-7.

47. Chen YK, Yang YX, Wei SJ, Song RH. Impact of Toxoplasma gondii infection on pregnancy outcomes in early pregnant women. *Chinese Journal of Schistosomiasis Control* 2014; **26**(3): 308-10.

48. Chintana T, Sukthana Y, Bunyakai B, Lekkla A. Toxoplasma gondii antibody in pregnant women with and without HIV infection. *The Southeast Asian journal of tropical medicine and public health* 1998; **29**(2): 383-6.

49. Chou CS, Lin LY, Chen KM, Lai SC. FlowCytomix analysis for Toxoplasma gondii infection in pregnant women in central Taiwan. *Journal of Obstetrics and Gynaecology* 2011; **31**(5): 375-9.

50. Brandao de Mattos CdC, Junqueira Franco Spegiorin LC, Meira CdS, et al. Anti-Toxoplasma gondii antibodies in pregnant women and their newborn infants in the region of Sao Jose do Rio Preto, Sao Paulo, Brazil. *Sao Paulo Medical Journal* 2011; **129**(4): 261-6.

51. Cong W, Dong XY, Meng QF, et al. Toxoplasma gondii Infection in Pregnant Women: A Seroprevalence and Case-Control Study in Eastern China. *BioMed Research International* 2015; **2015**.

52. Contreras MC, Escaff V, Salinas P, Saavedra T, Suárez M. Parasitic and viral marker detection in pregnant adolescents and their newborn infants at risk. *Revista chilena de obstetricia y ginecología* 1995; **60**(2): 85-9.

53. Costa GB, de Oliveira MC, Gadelha SR, et al. Infectious diseases during pregnancy in Brazil: Seroprevalence and risk factors. *Journal of Infection in Developing Countries* 2018; **12**(8): 657-65.

54. da Rocha É M, Lopes CWG, Ramos RAN, Alves LC. Risk factors for Toxoplasma gondii infection among pregnant women from the State of Tocantins, Northern Brazil. *Revista da Sociedade Brasileira de Medicina Tropical* 2015; **48**(6): 773-5.

55. Da Silva MG, Câmara JT, Vinaud MC, De Castro AM. Epidemiological factors associated with seropositivity for toxoplasmosis in pregnant women from gurupi, State of tocantins, Brazil. *Revista da Sociedade Brasileira de Medicina Tropical* 2014; **47**(4): 469-75.

56. de Moura FL, Amendoeira MRR, Bastos OMP, et al. Prevalence and risk factors for Toxoplasma gondii infection among pregnant and postpartum women attended at public healthcare facilities in the City of Niterói, State of Rio de Janeiro, Brazil. *Revista da Sociedade Brasileira de Medicina Tropical* 2013; **46**(2).

57. Alarcón de Noya B, Romero J, Sánchez E, et al. Despistaje de toxoplasmosis y enfermedad de Chagas en la consulta prenatal del Hospital Universitario de Caracas. *Rev Obstet Ginecol Venez* 2010; **70**(2): 75-81.

58. De Paschale M, Ceriani C, Cerulli T, et al. Antenatal screening for Toxoplasma gondii, Cytomegalovirus, rubella and Treponema pallidum infections in northern Benin. *Tropical Medicine & International Health* 2014; **19**(6): 743-6.

59. De Quadros RM, Da Rocha GC, Romagna G, De Oliveira JP, Ribeiro DM, Marques SMT. Toxoplasma gondii seropositivity and risk factors in pregnant women followed up by the Family Health Strategy. *Revista da Sociedade Brasileira de Medicina Tropical* 2015; **48**(3): 338-42.

60. Dentico P, Volpe A, Putoto G, et al. Toxoplasmosis in Kosovo pregnant women. *New Microbiologica* 2011; **34**(2): 203-7.

61. De Paschale M, Agrappi C, Clerici P, et al. Seroprevalence and incidence of Toxoplasma gondii infection in the Legnano area of Italy. *Clinical Microbiology and Infection* 2008; **14**(2): 186-9.

62. Dias RCF, Lopes-Mori FMR, Mitsuka-Breganó R, et al. Factors associated to infection by Toxoplasma gondii in pregnant women attended in basic health units in the city of Rolândia, Paraná, Brazil. *Revista do Instituto de Medicina Tropical de Sao Paulo* 2011; **53**(4): 185-91.

63. Diaz-SuáRez O, Estevez J. Seroepidemiol of toxoplasmosis in women of childbearing age from a marginal community of Maracaibo, Venezuela. *Revista do Instituto de Medicina Tropical de Sao Paulo* 2009; **51**(1): 13-7.

64. Doehring E, Reiter-Owona I, Bauer O, et al. Toxoplasma gondii antibodies in pregnant women and their newborns in Dar es Salaam, Tanzania. *American Journal of Tropical Medicine and Hygiene* 1995; **52**(6): 546-8.

65. Doğan K, Güraslan H, Özel G, Aydan Z, Yaşar L. Seroprevalence rates of Toxoplasma gondii, rubella, cytomegalovirus, syphilis, and hepatitis B, seroprevalences rate in the pregnant population in İstanbul. *Türkiye parazitolojii dergisi / Türkiye Parazitoloji Derneği = Acta parasitologica Turcica / Turkish Society for Parasitology* 2014; **38**(4): 228-33.

66. Doudou Y, Renaud P, Coralie L, et al. Toxoplasmosis among pregnant women: High seroprevalence and risk factors in Kinshasa, Democratic Republic of Congo. *Asian Pacific Journal of Tropical Biomedicine* 2014; **4**(1): 69-74.

67. Durdu B, Mutlu M. The seroprevalence of toxoplasma in healthy pregnants and evaluation of IgG aviditiy values. *Medical Journal of Bakirkoy* 2017; **13**(3): 140-4.

68. El Deeb HK, Salah-Eldin H, Khodeer S, Allah AA. Prevalence of Toxoplasma gondii infection in antenatal population in Menoufia governorate, Egypt. *Acta Tropica* 2012; **124**(3): 185-91.

69. Paul E, Kiwelu I, Mmbaga B, et al. Toxoplasma gondii seroprevalence among pregnant women attending antenatal clinic in Northern Tanzania. *Tropical Medicine and Health* 2018; **46**(1).

70. Elnahas A, Gerais AS, Elbashir MI, Eldien ES, Adam I. Toxoplasmosis in pregnant Sudanese women. *Saudi Medical Journal* 2003; **24**(8): 868-70.

71. El-Nawawy A, Soliman AT, El Azzouni O, et al. Maternal and neonatal prevalence of toxoplasma and cytomegalovirus (CMV) antibodies and hepatitis-B antigens in an Egyptian rural area. *Journal of Tropical Pediatrics* 1996; **42**(3): 154-7.

72. Elsafi SH, Al-Mutairi WF, Al-Jubran KM, Abu Hassan MM, Al Zahrani EM. Toxoplasmosis seroprevalence in relation to knowledge and practice among pregnant women in Dhahran, Saudi Arabia. *Pathogens and Global Health* 2015; **109**(8): 377-82.

73. Emelia O, Rahana AR, Mohamad Firdaus A, et al. IgG avidity assay: a tool for excluding acute toxoplasmosis in prolonged IgM titer sera from pregnant women. *Tropical biomedicine* 2014; **31**(4): 633-40.

74. Endris M, Belyhun Y, Moges F, et al. Seroprevalence and associated risk factors of Toxoplasma gondii in pregnant women attending in Northwest Ethiopia. *Iranian Journal of Parasitology* 2014; **9**(3): 407-14.

75. Ertug S, Okyay P, Turkmen M, Yuksel H. Seroprevalence and risk factors for toxoplasma infection among pregnant women in Aydin province, Turkey. *BMC Public Health* 2005; **5**.

76. Bahman Rahimi E, Aref T, Parisa M, Mahbubeh T. [Sero-prevalence of toxoplasma gondii infection among women in first trimester of pregnancy of women in Razavi- Khorasan Province]. *J Neyshabur Univ Med Sci* 2015; **3**(4): 10-8.

77. Evengård B, Lilja G, Capraru T, et al. A retrospective study of seroconversion against Toxoplasma gondii during 3000 pregnancies in Stockholm. *Scandinavian Journal of Infectious Diseases* 1999; **31**(2): 127-9.

78. Ferezin RI, Bertolini DA, Demarchi IG. [Prevalence of positive sorology for HIV, hepatitis B, toxoplasmosis and rubella in pregnant women from the northwestern region of the state of Paraná]. *Revista brasileira de ginecologia e obstetrícia : revista da Federação Brasileira das Sociedades de Ginecologia e Obstetrícia* 2013; **35**(2): 66-70.

79. Figueiró-Filho EA, Senefonte FRDA, Lopes AHA, et al. Frequency of HIV-1, rubella, syphilis, toxoplasmosis, cytomegalovirus, simple herpes virus, hepatitis B, hepatitis C, Chagas' disease and HTLV I/II infection in pregnant women of State of Mato Grosso do Sul. *Revista da Sociedade Brasileira de Medicina Tropical* 2007; **40**(2): 181-7.

80. Flatt A, Shetty N. Seroprevalence and risk factors for toxoplasmosis among antenatal women in London: a re-examination of risk in an ethnically diverse population. *European journal of public health* 2013; **23**(4): 648-52.

81. Fochi MML, Baring S, Spegiorin LCJF, et al. Prematurity and low birth weight did not correlate with anti-Toxoplasma gondii maternal serum profiles - A Brazilian report. *PLoS ONE* 2015; **10**(7).

82. Fonseca AL, Silva RA, Fux B, Madureira AP, de Sousa FF, Margonari C. Epidemiologic aspects of toxoplasmosis and evaluation of its seroprevalence in pregnant women. *Revista da Sociedade Brasileira de Medicina Tropical* 2012; **45**(3): 357-64.

83. Franck J, Garin YJF, Dumon H. LDBio-Toxo II immunoglobulin G Western blot confirmatory test for anti-Toxoplasma antibody detection. *Journal of Clinical Microbiology* 2008; **46**(7): 2334-8.

84. Franklin DM, Dror Z, Nishri Z. The prevalence and incidence of Toxoplasma antibodies in pregnant women. *Israel Journal of Medical Sciences* 1993; **29**(5): 285-6.

85. Friese K, Schroeder G, Beichert M, Melchert F. Prevalence of antibodies against Toxoplasma gondii in pregnant women and their newborns. *International Journal of Feto-Maternal Medicine* 1993; **6**(1): 21-5.

86. Freitas LC, Marques MRdV, Leite RBdCH, Holanda CMdCX, Barbosa VSdA. Seroprevalence of toxoplasmosis in pregnant women in a city in Rio Grande do Norte state, Brazil. *Rev patol trop* 2017; **46**(2): 147-58.

87. Frimpong C, Makasa M, Sitali L, Michelo C. Seroprevalence and determinants of toxoplasmosis in pregnant women attending antenatal clinic at the university teaching hospital, Lusaka, Zambia. *BMC Infectious Diseases* 2017; **17**(1).

88. Varol FG, Sayin NC, Soysüren S. Seroprevalance of toxoplasma gondii antibodies in antenatal population of Trakya region. *Turk Jinekoloji ve Obstetrik Dernegi Dergisi* 2011; **8**(2): 93-9.

89. Gamba EP, Nambei WS, Kamandji L. Integrated screening for HIV, syphilis, and toxoplasmosis among pregnant women in the Central African Republic. *Médecine et santé tropicales* 2013; **23**(4): 421-6.

90. Gao DL, Meng XJ, Zhang X, Qian YH, Lu B. Survey on toxoplasma gondii infection among key populations in Wuxi City. *Chinese Journal of Schistosomiasis Control* 2017; **29**(3): 352-4.

91. Garedaghi Y, Firozivand Y. Assessment of pregnant women toxoplasmosis by ELISA method in Miandoab city, Iran. *International Journal of Women's Health and Reproduction Sciences* 2017; **5**(1): 72-5.

92. Gebremedhin EZ, Abebe AH, Tessema TS, et al. Seroepidemiology of Toxoplasma gondii infection in women of child-bearing age in central Ethiopia. *BMC Infectious Diseases* 2013; **13**(1).

93. Gencer M, Cevizci S, Saçar S, et al. [Evaluation of anti-Toxoplasma gondii antibody distribution and risk factors among pregnant women admitted to obstetrics polyclinic of Canakkale Onsekiz Mart University Hospital]. *Türkiye parazitolojii dergisi / Türkiye Parazitoloji Derneği = Acta parasitologica Turcica / Turkish Society for Parasitology* 2014; **38**(2): 76-80.

94. Gilbert RE, Tookey PA, Cubitt WD, Ades AE, Masters J, Peckham CS. Prevalence of toxoplasma IgG among pregnant women in west London according to country of birth and ethnic group. *British Medical Journal* 1992; **306**(6871): 185.

95. Gonçalves MADS, De Matos CDCB, Spegiorin LCJF, Vaz-Oliani DCM, Oliani AH, De Mattos LC. Seropositivity rates for toxoplasmosis, rubella, syphilis, cytomegalovirus, hepatitis and HIV among pregnant women receiving care at a Public Health Service, São Paulo State, Brazil. *Brazilian Journal of Infectious Diseases* 2010; **14**(6): 601-5.

96. Guerra-Sanches F, Norberg AN, Covarrubias-Loayza EA, Aguillar-Uriarte MA, Madeira-Oliveira JT, Serra-Freire NM. Toxoplasmosis aguda en embarazadas asintomáticas de Rio de Janeiro, Brasil. *Revista Medica Herediana* 2014; **25**(4): 204-7.

97. Guerra García C, Fernández Sampedro J. Seroprevalence of Toxoplasma gondii in pregnant women. *Atencion primaria / Sociedad Española de Medicina de Familia y Comunitaria* 1995; **16**(3): 151-3.

98. Gündem NS, Ağir MÇ. Investigation of seroprevalences of rubella and toxoplasma gondii among pregnant women. *Anatolian Journal of Clinical Investigation* 2014; **8**(4): 152-7.

99. Kwon G-C, 고영현, 이민아, et al. Seroprevalence of Toxoplasmosis in Pregnant Women in Daejeon, Korea. *Laboratory Medicine Online* 2011; **1**(4): 190-4.

100. Hajsoleimani F, Ataeian A, Nourian AA, Mazloomzadeh S. Seroprevalence of toxoplasma gondii in pregnant women and bioassay of IgM positive cases in Zanjan, Northwest of Iran. *Iranian Journal of Parasitology* 2012; **7**(2): 82-6.

101. Han K, Shin DW, Lee TY, Lee YH. Seroprevalence of Toxoplasma gondii infection and risk factors associated with seropositivity of pregnant women in Korea. *Journal of Parasitology* 2008; **94**(4): 963-5.

102. Harma M, Harma M, Gungen N, Demir N. Toxoplasmosis in pregnant women in Sanliurfa, Southeastern Anatolia City, Turkey. *Journal of the Egyptian Society of Parasitology* 2004; **34**(2): 519-25.

103. Nahouli H, El Arnaout N, Chalhoub E, Anastadiadis E, El Hajj H. Seroprevalence of Anti-Toxoplasma gondii Antibodies among Lebanese Pregnant Women. *Vector-Borne and Zoonotic Diseases* 2017; **17**(12): 785-90.

104. Hershey DW, McGregor JA. Low prevalence of toxoplasma infection in a Rocky Mountain prenatal population. *Obstetrics and Gynecology* 1987; **70**(6): 900-2.

105. Hosseini SA, Dehgani N, Sharif M, et al. Serological survey of toxoplasmosis in pregnant women. *Journal of Mazandaran University of Medical Sciences* 2014; **24**(114): 146-50.

106. Hua HY, Tang F, Liu YX, et al. Survey of Toxoplasma gondii infection among pregnant women in Jiangsu Province, China. *Chinese Journal of Schistosomiasis Control* 2013; **25**(1): 56-8+79.

107. Hung CS, Su HW, Lee YL, et al. Seroprevalence, seroconversion, and risk factors for toxoplasmosis among pregnant women in Taipei, Taiwan. *Japanese Journal of Infectious Diseases* 2015; **68**(4): 312-7.

108. Iddawela D, Vithana SMP, Ratnayake C. Seroprevalence of toxoplasmosis and risk factors of Toxoplasma gondii infection among pregnant women in Sri Lanka: a cross sectional study. *BMC public health* 2017; **17**(1): 930.

109. Imam NFA, Azzam EAA, Attia AA. Seroprevalence of Toxoplasma gondii among pregnant women in Almadinah Almunawwarah KSA. *Journal of Taibah University Medical Sciences* 2016; **11**(3): 255-9.

110. Inagaki ADDM, De Oliveira LAR, De Oliveira MFB, et al. Seroprevalence of antibodies for toxoplasmosis, rubella, cytomegalovirus, syphilis and HIV among pregnant women in Sergipe. *Revista da Sociedade Brasileira de Medicina Tropical* 2009; **42**(5): 532-6.

111. Iqbal J, Khalid N. Detection of acute Toxoplasma gondii infection in early pregnancy by IgG avidity and PCR analysis. *Journal of Medical Microbiology* 2007; **56**(11): 1495-9.

112. Ahmad QI, Choh SA, Charoo BA, Ahmad SM, Ali W, Chaudhary J. Clinico-epidemiological profile of maternal and congenital toxoplasmosis in Kashmir valley: A hospital based study. *Journal of Pediatric Infectious Diseases* 2010; **5**(4): 333-7.

113. Barbosa IR, de Carvalho Xavier Holanda CM, de Andrade-Neto VF. Toxoplasmosis screening and risk factors amongst pregnant females in Natal, northeastern Brazil. *Transactions of the Royal Society of Tropical Medicine and Hygiene* 2009; **103**(4): 377-82.

114. El-Shahawy IS, Khalil MI, Bahnass MM. Seroprevalence of Toxoplasma gondii in women in Najran City, Saudi Arabia. *Saudi Medical Journal* 2014; **35**(9): 1143-6.

115. Jacquier P, Hohlfeld P, Vorkauf H, Zuber P. EPIDEMIOLOGY OF TOXOPLASMOSIS IN SWITZERLAND - NATIONAL STUDY OF PREVALENCE IN PREGNANT-WOMEN 1990-1991. *Schweizerische Medizinische Wochenschrift* 1995; **125**(8): S29-S38.

116. Jaqueti J, Hernández-García R, Nicolás D, Martínez-Hernández D, Navarro-Gallar F, García-Esteban RJ. Serology against Toxoplasma gondii in pregnant women. Development of prevalence rates in the course of 4 years. *Revista clínica española* 1991; **188**(6): 278-80.

117. Alghamdi J, Elamin MH, Alhabib S. Prevalence and genotyping of Toxoplasma gondii among Saudi pregnant women in Saudi Arabia. *Saudi Pharmaceutical Journal* 2016; **24**(6): 645-51.

118. Jula J, Girones G, Edao B, et al. Seroprevalence of Toxoplasma gondii infection in pregnant women attending antenatal care in southern Ethiopia. *Revista Espanola de Quimioterapia* 2018; **31**(4): 363-6.

119. Jenum PA, Stray-Pedersen B, Melby KK, et al. Incidence of Toxoplasma gondii infection in 35,940 pregnant women in Norway and pregnancy outcome for infected women. *Journal of Clinical Microbiology* 1998; **36**(10): 2900-6.

120. Jiang RL, Ma LH, Ma ZR, Hou G, Zhao Q, Wu X. Seroprevalence and associated risk factors of Toxoplasma gondii among Manchu pregnant women in northeastern China. *Microbial Pathogenesis* 2018; **123**: 398-401.

121. Josheghani SB, Moniri R, Taheri FB, Sadat S, Heidarzadeh Z. The prevalence of serum antibodies in TORCH infections during the first trimester of pregnancy in Kashan, Iran. *Iranian Journal of Neonatology* 2015; **6**(1): 8-12.

122. Jumaian NF. Seroprevalence and risk factors for Toxoplasma infection in pregnant women in Jordan. *Eastern Mediterranean Health Journal* 2005; **11**(1-2): 45-51.

123. Kamal AM, Ahmed AK, Abdellatif MZM, Tawfik M, Hassan EE. Seropositivity of toxoplasmosis in pregnant women by ELISA at Minia university hospital, Egypt. *Korean Journal of Parasitology* 2015; **53**(5): 605-10.

124. Karacan M, Batukan M, Çebi Z, et al. Screening cytomegalovirus, rubella and toxoplasma infections in pregnant women with unknown pre-pregnancy serological status. *Archives of Gynecology and Obstetrics* 2014.

125. Karunajeewa H, Siebert D, Hammond R, Garland S, Kelly H. Seroprevalence of varicella zoster virus, parvovirus B19 and Toxoplasma gondii in a Melbourne obstetric population: Implications for management. *Australian and New Zealand Journal of Obstetrics and Gynaecology* 2001; **41**(1): 23-8.

126. Kaur R, Gupta N, Nair D, Kakkar M, Mathur MD. Screening for TORCH infections in pregnant women: a report from Delhi. *The Southeast Asian journal of tropical medicine and public health* 1999; **30**(2): 284-6.

127. Khurana S, Bagga R, Aggarwal A, et al. Serological screening for antenatal toxoplasma infection in India. *Indian Journal of Medical Microbiology* 2010; **28**(2): 143-6.

128. Klufio CA, Delamare O, Amoa AB, Kariwiga G. The prevalence of toxoplasma antibodies in pregnant patients attending the Port Moresby General Hospital antenatal clinic: a seroepidemiological survey. *Papua and New Guinea medical journal* 1993; **36**(1): 4-9.

129. Laboudi M, Sadak A. Serodiagnosis of Toxoplasmosis: The effect of measurement of IgG avidity in pregnant women in Rabat in Morocco. *Acta Tropica* 2017; **172**: 139-42.

130. Lebech M, Larsen SO, Petersen E. Prevalence, incidence and geographical distribution of toxoplasma gondii antibodies in pregnant women in Denmark. *Scandinavian Journal of Infectious Diseases* 1993; **25**(6): 751-6.

131. Leone F, Allori B, Antognoli A, et al. Toxoplasmosis in pregnancy: Research on 2295 women in Rome and its province. *European Review for Medical and Pharmacological Sciences* 1996; **18**(5-6): 191-5.

132. Lin YL, Liao YS, Liao LR, Chen FN, Kuo HM, He S. Seroprevalence and sources of Toxoplasma infection among indigenous and immigrant pregnant women in Taiwan. *Parasitology Research* 2008; **103**(1): 67-74.

133. Linguissi LSG, Nagalo BM, Bisseye C, et al. Seroprevalence of toxoplasmosis and rubella in pregnant women attending antenatal private clinic at Ouagadougou, Burkina Faso. *Asian Pacific Journal of Tropical Medicine* 2012; **5**(10): 810-3.

134. Lito D, Francisco T, Salva I, Tavares MN, Oliveira R, Neto MT. TORCH serology and group B Streptococcus screening analysis in the population of a maternity. *Acta Medica Portuguesa* 2013; **26**(5): 549-54.

135. Liu Q, Wei F, Gao S, et al. Toxoplasma gondii infection in pregnant women in China. *Transactions of the Royal Society of Tropical Medicine and Hygiene* 2009; **103**(2): 162-6.

136. Lobo ML, Patrocinio G, Sevivas T, De Sousa B, Matos O. Portugal and Angola: Similarities and differences in Toxoplasma gondii seroprevalence and risk factors in pregnant women. *Epidemiology and Infection* 2017; **145**(1): 30-40.

137. Lopes-Mori FMR, Mitsuka-Breganó R, Bittencourt LHFDB, et al. Gestational toxoplasmosis in Paraná State, Brazil: Prevalence of IgG antibodies and associated risk factors. *Brazilian Journal of Infectious Diseases* 2013; **17**(4): 405-9.

138. Lopes FMR, Mitsuka-Breganó R, Gonçalves DD, et al. Factors associated with seropositivity for anti-Toxoplasma gondii antibodies in pregnant women of Londrina, Paraná, Brazil. *Memorias do Instituto Oswaldo Cruz* 2009; **104**(2): 378-82.

139. López-Fabal F, Gómez-Garcés JL. Serological markers of Spanish and immigrant pregnant women in the south of Madrid during the period 2007-2010. *Revista Espanola de Quimioterapia* 2013; **26**(2): 108-11.

140. Liu H, Xin KS, Jiang YH, Jiang ZY. Seroprevalence of Toxoplasma gondii infection among pregnant women in Shandong Province, China. *Research Journal of Medical Sciences* 2014; **8**(1): 42-4.

141. Luyasu V, Schroeder G, Wacquez M, Bohy E. Follow-up of pregnant women for Toxoplasma antibodies with the IMX analyzer in the brabant walloon region of Belgium. *International Journal of Feto-Maternal Medicine* 1993; **6**(1): 26-32.

142. Maggi P, Volpe A, Carito V, et al. Surveillance of toxoplasmosis in pregnant women in Albania. *New Microbiologica* 2009; **32**(1): 89-92.

143. Elamin MH, Al-Olayan EM, Omer SA, Alagaili AN, Mohammed OB. Molecular detection and prevalence of Toxoplasma gondii in pregnant women in Sudan. *African Journal of Microbiology Research* 2012; **6**(2): 308-11.

144. Mahdy MAK, Alareqi LMQ, Abdul-Ghani R, et al. A community-based survey of Toxoplasma gondii infection among pregnant women in rural areas of Taiz governorate, Yemen: The risk of waterborne transmission. *Infectious Diseases of Poverty* 2017; **6**(1).

145. Lappalainen M, Koskela P, Hedman K, et al. Incidence of primary toxoplasma infections during pregnancy in Southern Finland: A prospective cohort study. *Scandinavian Journal of Infectious Diseases* 1992; **24**(1): 97-104.

146. Majid A, Khan S, Jan AH, et al. Chronic toxoplasmosis and possible risk factors associated with pregnant women in Khyber Pakhtunkhwa. *Biotechnology & Biotechnological Equipment* 2016; **30**(4): 733-6.

147. El Mansouri B, Rhajaoui M, Sebti F, et al. Seroprevalence of toxoplasmosis in pregnant women in Rabat, Morocco. *Bulletin de la Societe de Pathologie Exotique* 2007; **100**(4): 289-90.

148. Marcinek P, Nowakowska D, Szaflik K, Spiewak E, Małafiej E, Wilczyński J. Analysis of complications during pregnancy in women with serological features of acute toxoplasmosis or acute parvovirosis. *Ginekologia polska* 2008; **79**(3): 186-91.

149. Liassides M, Christodoulou V, Moschandreas J, et al. Toxoplasmosis in female high school students, pregnant women and ruminants in Cyprus. *Transactions of the Royal Society of Tropical Medicine and Hygiene* 2016; **110**(6): 359-66.

150. Martínez Méndez D, Martínez Leal E, Oberto Perdigón L, Navas Yamarte P. Seroprevalencia de la toxoplasmosis en mujeres que asistieron al Hospital “Dr. Rafael Gallardo”. Coro, estado Falcón. *Revista de la Sociedad Venezolana de Microbiología* 2009; **29**(1): 49-51.

151. Mioranza SDL, Meireles LR, Mioranza EL, De Andrade Jr HF. Serological evidence of acute Toxoplasma gondii infection in pregnant women in Cascavel, Paraná. *Revista da Sociedade Brasileira de Medicina Tropical* 2008; **41**(6): 628-34.

152. Mohamed K, Bahathiq A, Degnah N, et al. Detection of Toxoplasma gondii infection and associated risk factors among pregnant women in Makkah Al Mukarramah, Saudi Arabia. *Asian Pacific Journal of Tropical Disease* 2016; **6**(2): 113-9.

153. Mohammad Rostami N, Zeynab F, Ehsan Nazemalhosseini M, et al. [Prevalence of celiac disease and toxoplasmosis during pregnancy]. *Med Sci J Islam Azad Univ* 2013; **22**(4): 288-93.

154. Mohammad HIA, Amin TT, Balaha MH, Moghannum MSA. Toxoplasmosis among the pregnant women attending a Saudi maternity hospital: Seroprevalence and possible risk factors. *Annals of Tropical Medicine and Parasitology* 2010; **104**(6): 493-504.

155. Morris AJ, Croxson MC. Serological evidence of Toxoplasma gondii infection among pregnant women in Auckland. *New Zealand Medical Journal* 2004; **117**(1189).

156. Pegha Moukandja I, Ngoungou EB, Lemamy GJ, et al. Non-malarial infectious diseases of antenatal care in pregnant women in Franceville, Gabon. *BMC Pregnancy and Childbirth* 2017; **17**(1).

157. Mousavi P, Mirhendi H, Mohebali M, et al. Detection of Toxoplasma gondi in acute and chronic phases of infection in immunocompromised patients and pregnant women with real-time PCR assay using TaqMan fluorescent probe. *Iranian Journal of Parasitology* 2018; **13**(3): 373-81.

158. Mpiga Mickoto R, Akue JP, Bisvigou U, Mayi Tsonga S, Nkoghe D. [Serological study on toxoplasmosis among pregnant women from Franceville, Gabon]. *Bulletin de la Société de pathologie exotique (1990)* 2010; **103**(1): 41-3.

159. Mumcuoglu I, Toyran A, Cetin F, et al. [Evaluation of the toxoplasmosis seroprevalence in pregnant women and creating a diagnostic algorithm]. *Mikrobiyoloji bülteni* 2014; **48**(2): 283-91.

160. Sirin MC, Agus N, Yilmaz N, et al. Seroprevalence of Toxoplasma gondii, Rubella virus and Cytomegalovirus among pregnant women and the importance of avidity assays. *Saudi Medical Journal* 2017; **38**(7): 727-32.

161. Murebwayire E, Njanaake K, Ngabonziza JCS, Njunwa KJ, Jaoko W. Seroprevalence and risk factors of Toxoplasma gondii infection among pregnant women attending antenatal care in Kigali, Rwanda. *Tanzania Journal of Health Research* 2017; **19**(1).

162. Mwambe B, Mshana SE, Kidenya BR, et al. Sero-prevalence and factors associated with Toxoplasma gondii infection among pregnant women attending antenatal care in Mwanza, Tanzania. *Parasites and Vectors* 2013; **6**(1).

163. Nabias R, Ngouamizokou A, Migot-Nabias F, Mbou-Moutsimbi RA, Lansoud-Soukate J. Serological investigation of toxoplasmosis in patients of the M.I.P. center of Franceville (Gabon). *Bulletin de la Société de pathologie exotique (1990)* 1998; **91**(4): 318-20.

164. Naghili B, Abbasalizadeh S, Tabrizi S, et al. Comparison of IIF, ELISA and IgG avidity tests for the detection of anti-toxoplasma antibodies in single serum sample from pregnant women. *Infezioni in Medicina* 2017; **25**(1): 50-6.

165. Nash JQ, Chissel S, Jones J, Warburton F, Verlander NQ. Risk factors for toxoplasmosis in pregnant women in Kent, United Kingdom. *Epidemiology and Infection* 2005; **133**(3): 475-83.

166. Nasir IA, Aderinsayo AH, Mele HU, Aliyu MM. Prevalence and associated risk factors of toxoplasma gondii antibodies among pregnant women attending maiduguri teaching hospital, Nigeria. *Journal of Medical Sciences (Faisalabad)* 2015; **15**(3): 147-54.

167. Ndiaye D, Sène PD, Ndiaye M, Faye B, Ndiaye JL, Ndir O. [Update on toxoplasmosis prevalence based on serological tests in pregnant women in Dakar, Senegal from 2002 to 2006]. *Médecine tropicale : revue du Corps de santé colonial* 2011; **71**(1): 101-2.

168. Ndumbe PM, Andela A, Nkemnkeng-Asong J, Watonsi E, Nyambi P. Prevalence of infections affecting the child among pregnant women in Yaounde, Cameroon. *Medical Microbiology and Immunology* 1992; **181**(3): 127-30.

169. Niemiec KT, Raczyński P, Markiewicz K, Leibschang J, Ceran A. The prevalence of Toxoplasma gondii infection among 2016 pregnant women and their children in the Institute of Mother and Child in Warsaw. *Wiadomości parazytologiczne* 2002; **48**(3): 293-9.

170. Nijem KI, Al-Amleh S. Seroprevalence and associated risk factors of toxoplasmosis in pregnant women in Hebron district, Palestine. *Eastern Mediterranean Health Journal* 2009; **15**(5): 1278-84.

171. Nissapatorn V, Noor Azmi MA, Cho SM, et al. Toxoplasmosis: Prevalence and risk factors. *Journal of Obstetrics and Gynaecology* 2003; **23**(6): 618-24.

172. Nissapatorn V, Suwanrath C, Sawangjaroen N, Ling LY, Chandeying V. Toxoplasmosis-serological evidence and associated risk factors among pregnant women in southern Thailand. *American Journal of Tropical Medicine and Hygiene* 2011; **85**(2): 243-7.

173. Njunda AL, Assob JCN, Nsagha DS, Kamga HL, Nde PF, Yugah VC. Seroprevalence of toxoplasma gondii infection among pregnant women in Cameroon. *Journal of Public Health in Africa* 2011; **2**(2): 98-101.

174. Njunda AL, Nsagha DS, Assob JCN, Kamga HFF, Tafili RT, Achidi EA. Seroepidemiology of toxoplasmosis in pregnant women attending the University Teaching Hospital in Yaounde, Cameroon. *International Journal of Health Research* 2011; **4**(1): 1-9.

175. Nourollahpour Shiadeh M, Rostami A, Pearce BD, et al. The correlation between Toxoplasma gondii infection and prenatal depression in pregnant women. *European Journal of Clinical Microbiology and Infectious Diseases* 2016; **35**(11): 1829-35.

176. Nowakowska D, Slaska M, Kostrzewska E, Wilczyński J. Anti - T. gondii antibody concentration in sera of pregnant women in the sample of Lódź population. *Wiadomości parazytologiczne* 2001; **47 Suppl 1**: 83-9.

177. Nowakowska D, Wujcicka W, Sobala W, piewak E, Gaj Z, Wilczyński J. Age-associated prevalence of Toxoplasma gondii in 8281 pregnant women in Poland between 2004 and 2012. *Epidemiology and Infection* 2013; **142**(3): 656-61.

178. Ocak S, Zeteroglu S, Ozer C, Dolapcioglu K, Gungoren A. Seroprevalence of Toxoplasma gondii, rubella and cytomegalovirus among pregnant women in southern Turkey. *Scandinavian Journal of Infectious Diseases* 2007; **39**(3): 231-4.

179. Okyay AG, Karateke A, Yula E, Inci M, Şilfeler DB, Motor VK. Seroprevalance of Toxoplasma IgG among pregnant women in the province of Hatay and contribution of avidity test to the diagnose. *Turk Jinekoloji ve Obstetrik Dernegi Dergisi* 2013; **10**(3): 160-4.

180. Ouermi D, Simpore J, Belem AMG, et al. Co-Infection of Toxoplasma gondii with HBV in HIV-Infected and Uninfected Pregnant Women in Burkina Faso. *Pakistan Journal of Biological Sciences* 2009; **12**(17): 1188-93.

181. Ozekinci T, Suay A, Karasahin O, Akpolat N, Mete M, Atmaca S. Assessment of toxoplasma IgG avidity test results in pregnant women. *Saudi Medical Journal* 2005; **26**(4): 682-3.

182. Parlak M, Çim N, Erdin BN, Güven A, Bayram Y, Yıldızhan R. Seroprevalence of Toxoplasma, Rubella, and Cytomegalovirus among pregnant women in Van. *Turk Jinekoloji ve Obstetrik Dernegi Dergisi* 2015; **12**(2): 79-82.

183. Puccio G, Cajozzo C, Canduscio LA, et al. Epidemiology of Toxoplasma and CMV serology and of GBS colonization in pregnancy and neonatal outcome in a Sicilian population. *Italian Journal of Pediatrics* 2014; **40**(1).

184. Punda-Polić V, Tonkić M, Čapkun V. Prevalence of antibodies to Toxoplasma gondii in the female population of the County of Split Dalmatia, Croatia. *European Journal of Epidemiology* 2000; **16**(9): 875-7.

185. Qin W, Hu DC, Pang HL. [Detection the serum IgM and IgG specific for TORCH in 1307 women in the period of pre-pregnancy and pregnancy]. *Zhonghua shi yan he lin chuang bing du xue za zhi = Zhonghua shiyan he linchuang bingduxue zazhi = Chinese journal of experimental and clinical virology* 2011; **25**(4): 292-4.

186. Radoń-Pokracka M, Piasecki M, Lachowska A, et al. Assessment of the implementation of the infectious diseases screening programmes among pregnant women in the Lesser Poland region and comparison with similar programmes conducted in other European Union countries. *Ginekologia polska* 2017; **88**(3): 151-5.

187. Rajaii M, Pourhassan A, Asle-Rahnamaie-Akbari N, et al. Seroepidemiology of toxoplasmosis in childbearing women of Northwest Iran. *Infezioni in Medicina* 2013; **21**(3): 194-200.

188. Ramos JM, Milla A, Rodríguez JC, Padilla S, Masiá M, Gutiérrez F. Seroprevalence of Toxoplasma gondii infection among immigrant and native pregnant women in Eastern Spain. *Parasitology Research* 2011; **109**(5): 1447-52.

189. Ramsewak S, Gooding R, Ganta K, Seepersadsingh N, Adesiyun AA. Seroprevalence and risk factors of Toxoplasma gondii infection among pregnant women in Trinidad and Tobago. *Rev Panam Salud Publica* 2008; **23**(3): 164-70.

190. Ravindranath C, Kumari BA, Golia S, Venkatesha D. Toxoplasmosis in pregnancy: A silent threat. *Journal of Pure and Applied Microbiology* 2012; **6**(3): 1199-202.

191. Rebouças EC, Dos Santos EL, Carmo MLSD, Cavalcante Z, Favali C. Seroprevalence of Toxoplasma infection among pregnant women in Bahia, Brazil. *Transactions of the Royal Society of Tropical Medicine and Hygiene* 2011; **105**(11): 670-1.

192. Reiche EM, Morimoto HK, Farias GN, et al. Prevalence of American trypanosomiasis, syphilis, toxoplasmosis, rubella, hepatitis B, hepatitis C, human immunodeficiency virus infection, assayed through serological tests among pregnant patients, from 1996 to 1998, at the Regional University Hospital Norte do Paraná. *Revista da Sociedade Brasileira de Medicina Tropical* 2000; **33**(6): 519-27.

193. Reis MM, Tessaro MM, Azevedo PA. Perfil sorológico para toxoplasmose em gestantes de um hospital público de Porto Alegre. *Rev Bras Ginecol Obstet* 2006; **28**(3): 158-64.

194. Ribeiro AC, Mutis MS, Fernandes O. Association of the presence of residual anti-Toxoplasma gondii IgM in pregnant women and their respective family groups in Miracema, Northwest Rio de Janeiro, Brazil. *Memorias do Instituto Oswaldo Cruz* 2008; **103**(6): 591-4.

195. Ribes Bautista A, Saniger Herrera JM, Reche Navarro C, Segovia Martínez A, Peis Redondo JI, Cruz Ríos MC. Serologic study of vertically transmitted infections in pregnant women attending 3 health centers in Jaén. *Revista española de salud pública* 1996; **70**(3): 313-8.

196. Rodier MH, Berthonneau J, Bourgoin A, et al. Seroprevalences of toxoplasma, malaria, rubella, cytomegalovirus, HIV and treponemal infections among pregnant women in Cotonou, Republic of Benin. *Acta Tropica* 1995; **59**(4): 271-7.

197. Rosso F, Les JT, Agudelo A, et al. Prevalence of infection with Toxoplasma gondii among pregnant women in Cali, Colombia, South America. *American Journal of Tropical Medicine and Hygiene* 2008; **78**(3): 504-8.

198. Ruffini E, Compagnoni L, Tubaldi L, et al. Le infezioni congenite e perinatali nella regione Marche (Italia). Studio Epidemiologico. *Infezioni in Medicina* 2014; **22**(3): 213-21.

199. Sagel U, Mikolajczyk RT, Kraemer A. Seasonal trends in acute toxoplasmosis in pregnancy in the federal state of Upper Austria. *Clinical Microbiology and Infection* 2010; **16**(5): 516-7.

200. Saki J, Shafieenia S, Foroutan-Rad M. Seroprevalence of toxoplasmosis in diabetic pregnant women in southwestern of Iran. *Journal of Parasitic Diseases* 2016; **40**(4): 1586-9.

201. Sakikawa M, Noda S, Hanaoka M, et al. Anti-Toxoplasma antibody prevalence, primary infection rate, and risk factors in a study of toxoplasmosis in 4,466 pregnant women in Japan. *Clinical and Vaccine Immunology* 2012; **19**(3): 365-7.

202. Câmara JT, Silva MG, Castro AM. Prevalence of toxoplasmosis in pregnant women in two reference centers in a city in Northeast Brazil. *Revista brasileira de ginecologia e obstetricia : revista da Federacao Brasileira das Sociedades de Ginecologia e Obstetricia* 2015; **37**(2): 64-70.

203. dos Santos JI, Lopes MA, Deliège-Vasconcelos E, et al. Seroprevalence of HIV, HTLV-I/II and other perinatally-transmitted pathogens in Salvador, Bahia. *Revista do Instituto de Medicina Tropical de São Paulo* 1995; **37**(4): 343-8.

204. Sartori AL, Minamisava R, Avelino MM, Martins CA. Prenatal screening for toxoplasmosis and factors associated with seropositivity of pregnant women in Goiânia, Goiás. *Revista Brasileira de Ginecologia e Obstetricia* 2011; **33**(2): 93-8.

205. Sekla L, Stackiw W, Rodgers S. A serosurvey of toxoplasmosis in Manitoba. *Canadian Journal of Public Health* 1981; **72**(2): 111-7.

206. Selek MB, Bektöre B, Baylan O, Özyurt M. Serological Investigation of Toxoplasma gondii on Pregnant Women and Toxoplasmosis Suspected Patients Between 2012-2014 Years on a Tertiary Training Hospital. *Türkiye parazitolojii dergisi / Türkiye Parazitoloji Derneği = Acta parasitologica Turcica / Turkish Society for Parasitology* 2015; **39**(3): 200-4.

207. Stephen S, Anitharaj V, Ghose S, Pradeep J. Seroprevalence of Toxoplasma gondii in healthy pregnant women of Puducherry. *Journal of Krishna Institute of Medical Sciences University* 2017; **6**(4): 134-6.

208. Shamakhteh K, Javad Nouri M, Jafarideh Y, Saki A. The relationship between premature rupture of membrane and toxoplasma gondii infection in pregnant women. *Iranian Journal of Obstetrics, Gynecology and Infertility* 2016; **19**(23): 7-14.

209. Sharbatkhori M, Moghaddam YD, Pagheh AS, Mohammadi R, Mofidi HH, Shojaee S. Seroprevalence of Toxoplasma gondii infections in pregnant women in Gorgan City, Golestan Province, Northern Iran-2012. *Iranian Journal of Parasitology* 2014; **9**(2): 181-7.

210. Shieh M, Didehdar M, Hajihossein R, Ahmadi F, Eslamirad Z. Toxoplasmosis: Seroprevalence in pregnant women, and serological and molecular screening in neonatal umbilical cord blood. *Acta Tropica* 2017; **174**: 38-44.

211. Shirdel S, Sharbatkhori M, Pagheh AS, Dadimoghadam Y, Soosaraie M, Gholami S. *Journal of Mazandaran University of Medical Sciences* 2017; **27**(152): 63-71.

212. Simpore J, Savadogo A, Ilboudo D, et al. Toxoplasma gondii, HCV, and HBV seroprevalence and co-infection among HIV-positive and -negative pregnant women in Burkina Faso. *Journal of Medical Virology* 2006; **78**(6): 730-3.

213. Singh S, Pandit AJ. Incidence and prevalence of toxoplasmosis in Indian pregnant women: A prospective study. *American Journal of Reproductive Immunology* 2004; **52**(4): 276-83.

214. Singh M, Ranjan R, Pradeep Y, Quereshi S, Sahu M. Seroprevalence of toxoplasmosis in pregnant females attending a tertiary care hospital in Uttar Pradesh, India and its effect on perinatal morbidity and mortality. *Acta Medica International* 2016; **3**(1): 50-5.

215. Sitoe SPBL, Rafael B, Meireles LR, de Andrade Jr HF, Thompson R. Preliminary report of HIV and Toxoplasma gondii occurrence in pregnant women from Mozambique. *Revista do Instituto de Medicina Tropical de Sao Paulo* 2010; **52**(6): 291-5.

216. Song KJ, Shin JC, Shin HJ, Nam HW. Seroprevalence of toxoplasmosis in Korean pregnant women. *The Korean journal of parasitology* 2005; **43**(2): 69-71.

217. Spalding SM, Reis Annendoeira MR, Klein CH, Ribeiro LC. Serological screening and toxoplasmosis exposure factors among pregnant women in South of Brazil. *Revista da Sociedade Brasileira de Medicina Tropical* 2005; **38**(2): 173-7.

218. Sroka S, Bartelheimer N, Winter A, et al. Prevalence and risk factors of toxoplasmosis among pregnant women in Fortaleza, Northeastern Brazil. *American Journal of Tropical Medicine and Hygiene* 2010; **83**(3): 528-33.

219. Sukthana Y. Difference of Toxoplasma gondii antibodies between Thai and Austrian pregnant women. *The Southeast Asian journal of tropical medicine and public health* 1999; **30**(1): 38-41.

220. Tabatabaie F, Mafi M, Mafi H, et al. Seroprevalence of and risk factors for Toxoplasma gondii among pregnant women in abyek township of Qazvin province, Iran (2013). *Asian Journal of Pharmaceutical and Clinical Research* 2015; **8**(1): 1-3.

221. Tamer GS, Dundar D, Caliskan E. Seroprevalence of Toxoplasma gondii, rubella and cytomegalovirus among pregnant women in western region of Turkey. *Clinical and Investigative Medicine* 2009; **32**(1): E43-E7.

222. Tlamcani Z, Yahyaoui G, Mahmoud M. Prevalence of immunity to toxoplasmosis among pregnant women in UniversityHospital Center Hassan II of FEZ city (Morocco). *Acta Medica International* 2017; **4**(1): 43-5.

223. Toklu GD. Antibodies frequency against toxoplasmosis, Rubella virus and cytomegalovirus in pregnant women. *Journal of Clinical and Analytical Medicine* 2013; **4**(1): 38-40.

224. De Tové YSS, Hounto AO, Vodouhe MV, et al. Seroprevalence and factors associated with toxoplasmosis in pregnant women living in rural areas in Benin. *Pan African Medical Journal* 2018; **29**.

225. Ustaçelebi S, Köksal I, Cantürk H, Saify SJ, Ersöz D, Sellioglu B. Detection of antibodies against TORCH agents during pregnancy. *Mikrobiyoloji bülteni* 1986; **20**(1): 1-8.

226. Yad Yad MJ, Jomehzadeh N, Sameri MJ, Noorshahi N. Seroprevalence of anti-toxoplasma gondii antibodies among pregnant woman in south Khuzestan, Iran. *Jundishapur Journal of Microbiology* 2014; **7**(5).

227. Yang WL, Wang Y. Investigation on Toxoplasma gondii infection in childbearing age and pregnant women in Langfang City, Hebei Province. *Chinese Journal of Schistosomiasis Control* 2016; **28**(2): 210-1.

228. Yanping Z, Renhao S. Investigation on pregnancy outcomes and risk factors in pregnant women in-fected with Toxoplasma gondii. *Chinese Journal of Schistosomiasis Control* 2014; (2): 221-3.

229. Yasmeen A, Prasad SR, Sheela SR, Krishnappa J. Screening of pregnant women for anti-Toxoplasma antibodies and their newborn for vertical transmission. *Journal of Clinical and Diagnostic Research* 2017; **11**(10): DC04-DC7.

230. Zemene E, Yewhalaw D, Abera S, Belay T, Samuel A, Zeynudin A. Seroprevalence of Toxoplasma gondii and associated risk factors among pregnant women in Jimma town, Southwestern Ethiopia. *BMC Infectious Diseases* 2012; **12**.

231. Zhang AM, Zhang T, Hao ZY. A seroepidemic survey on the infection of toxoplasma in pregnant women and its significance to better child-bearing. *Zhonghua liu xing bing xue za zhi = Zhonghua liuxingbingxue zazhi* 1996; **17**(5): 278-80.

232. Zhang QQ, Cheng JZ, Wang FH. Correlation between TORCH infections and abnormal pregnant outcomes. *Chinese Journal of Schistosomiasis Control* 2013; **25**(2): 209-10.

233. Zhou JJ, Tao LL. Seroprevalence and risk factors of Toxoplasma gondii infection among pregnant women in Wuxi region. *Chinese Journal of Schistosomiasis Control* 2015; **27**(6): 604-7.

234. Xia W, Zhang XJ, Chen XW. Investigation of different pregnant results of pregnant women infected with Toxoplasma gondii in Nanjing region. *Chinese Journal of Schistosomiasis Control* 2011; **23**(2): 183-6.

235. Wang Y, Yang WL. Prevalence and awareness of Toxoplasma gondii of pregnant women in Bazhou City, Hebei Province. *Chinese Journal of Schistosomiasis Control* 2016; **28**(3): 340-2.

236. Wanachiwanawin D, Sutthent R, Chokephaibulkit K, Mahakittikun V, Ongrotchanakun J, Monkong N. Toxoplasma gondii antibodies in HIV and non-HIV infected Thai pregnant women. *Asian Pacific Journal of Allergy and Immunology* 2001; **19**(4): 291-3.

237. Wam EC, Sama LF, Ali IM, Ebile WA, Aghangu LA, Tume CB. Seroprevalence of Toxoplasma gondii IgG and IgM antibodies and associated risk factors in women of child-bearing age in Njinikom, NW Cameroon. *BMC research notes* 2016; **9**(1): 406.

238. Völker F, Cooper P, Bader O, et al. Prevalence of pregnancy-relevant infections in a rural setting of Ghana. *BMC Pregnancy and Childbirth* 2017; **17**(1).

239. Vilibic-Cavlek T, Ljubin-Sternak S, Ban M, Kolaric B, Sviben M, Mlinaric-Galinovic G. Seroprevalence of TORCH infections in women of childbearing age in Croatia. *Journal of Maternal-Fetal and Neonatal Medicine* 2011; **24**(2): 280-3.

240. Vial P, Torres-Pereyra J, Stagno S, et al. Serological study of cytomegalovirus, herpes simplex and rubella virus, hepatitis B and Toxoplasma gondii in 2 populations of pregnant women in Santiago, Chile. *Boletín de la Oficina Sanitaria Panamericana Pan American Sanitary Bureau* 1985; **99**(5): 528-38.

241. Vaz AJ, Guerra EM, Ferratto LC, de Toledo LA, Azevedo Neto RS. Positive serology of syphilis, toxoplasmosis and Chagas' disease in pregnant women in their first visit to health centers in a metropolitan area, Brazil. *Revista de saúde pública* 1990; **24**(5): 373-9.

242. Vaz RS, Thomaz-Soccol V, Sumikawa E, Guimarães ATB. Serological prevalence of Toxoplasma gondii antibodies in pregnant women from Southern Brazil. *Parasitology Research* 2010; **106**(3): 661-5.

243. Varella IS, Canti ICT, Santos BR, et al. Prevalence of acute toxoplasmosis infection among 41,112 pregnant women and the mother-to-child transmission rate in a public hospital in South Brazil. *Memorias do Instituto Oswaldo Cruz* 2009; **104**(2): 383-8.

244. Varella IS, Wagner MB, Darela AC, Nunes LM, Müller RW. Seroprevalence of toxoplasmosis in pregnant women. *Jornal de pediatria* 2003; **79**(1): 69-74.

245. Varaghchi JR, Shadmehri AA, Shadmehri AA. Seroepidemiology of toxoplasmosis in women referred to Birjand milad genetic counseling center during 2011-2013. *Journal of Kerman University of Medical Sciences* 2015; **22**(5): 524-32.

246. Van Enter BJD, Lau YL, Ling CL, et al. Seroprevalence of toxoplasma gondii infection in refugee and migrant pregnant women along the Thailand-myanmar border. *American Journal of Tropical Medicine and Hygiene* 2017; **97**(1): 232-5.

247. Valcavi PP, Natali A, Soliani L, Montali S, Dettori G, Cheezi C. Prevalence of anti-Toxoplasma gondii antibodies in the population of the area of Parma (Italy). *European Journal of Epidemiology* 1995; **11**(3): 333-7.

248. Uysal A, Cüce M, Taner CE, et al. Prevalence of congenital toxoplasmosis among a series of Turkish women. Prevalencia de toxoplasmosis congénita en una serie de mujeres en Turquía; 2013. p. 471-6.
